# Studying Soft Interfaces with Shear Waves: Principles and Applications of the Quartz Crystal Microbalance (QCM)

**DOI:** 10.3390/s21103490

**Published:** 2021-05-17

**Authors:** Diethelm Johannsmann, Arne Langhoff, Christian Leppin

**Affiliations:** Institute of Physical Chemistry, Clausthal University of Technology, Arnold-Sommerfeld-Straße 4, 38678 Clausthal-Zellerfeld, Germany; arne.langhoff@tu-clausthal.de (A.L.); christian.leppin@tu-clausthal.de (C.L.)

**Keywords:** quartz crystal microbalance, QCM-D, EQCM, label-free biosensing, high-frequency rheology, high-frequency contact mechanics

## Abstract

The response of the quartz crystal microbalance (QCM, also: QCM-D for “QCM with Dissipation monitoring”) to loading with a diverse set of samples is reviewed in a consistent frame. After a brief introduction to the advanced QCMs, the governing equation (the small-load approximation) is derived. Planar films and adsorbates are modeled based on the acoustic multilayer formalism. In liquid environments, viscoelastic spectroscopy and high-frequency rheology are possible, even on layers with a thickness in the monolayer range. For particulate samples, the contact stiffness can be derived. Because the stress at the contact is large, the force is not always proportional to the displacement. Nonlinear effects are observed, leading to a dependence of the resonance frequency and the resonance bandwidth on the amplitude of oscillation. Partial slip, in particular, can be studied in detail. Advanced topics include structured samples and the extension of the small-load approximation to its tensorial version.

## Table of Contents

1.Introduction22.Forced Vibrations, Complex Resonance Frequencies63.Techniques of Read-Out83.1.Oscillator Circuits93.2.Impedance Analysis93.3.Ring-Down103.4.Multi-Frequency Lock-In Amplification103.5.Fast Measurements, Modulation Experiments113.6.Noise and Drift134.The Acoustic Multilayer Formalism and its Consequences144.1.Qualitative Data inspection144.2.The Small-Load Approximation in 1D (Parallel-Plate Model)144.3.Inertial Loading174.4.Semi-Infinite Viscoelastic Media174.5.Films in Air214.5.1.Very Thin Films (Sauerbrey Limit)234.5.2.Infinite Thickness234.5.3.Thin Viscoelastic Films234.5.4.The Film Resonance254.6.Layers Adsorbed from a Liquid Phase274.6.1.General274.6.2.Thin Adsorbates284.6.3.Thick Layers324.7.Viscoelastic Dispersion and High-Frequency Rheology334.8.Slip345.Non-Planar Samples355.1.Point Contacts with Large Objects Clamped in Space by Inertia355.2.Large Amplitudes, Partial Slip365.3.Structured Samples, Numerical Calculations405.4.Roughness426.Coupled Resonances436.1.The Sphere with Moderate Mass436.2.Influence of Rotation on the Frequency Shift466.3.Other Types of Coupled Resonances497.Piezoelectric Stiffening508.Beyond the Parallel-Plate Model518.1.Energy Trapping, Compressional Waves518.2.Anharmonic Sidebands548.3.Towards 3D-Modelling: The Small-Load Approximation in Tensor Form558.4.The 4-Element Circuit and the Electromechanical Analogy588.5.Amplitude of Oscillation, Effective Area608.6.Modal Mass, Sauerbrey Equation for Plates with Energy Trapping619.Combined Instruments629.1.The Electrochemical QCM (EQCM)639.2.Combination with Optical Reflectometry63References
71

## 1. Introduction

The use of the quartz crystal microbalance (QCM) in diverse areas keeps growing. Among its advantages is its simplicity. It is not difficult to mount the resonator plate in one way or another. Electrical interrogation of the resonance parameters is not difficult either. Simplicity entails versatility. The QCM can easily be combined with electrochemistry, optical reflectometry, scanning force microscopy, and other instruments of interface analysis. 

Reviews on the QCM can be found in [1,2,3,4,5,6]. The QCM becomes slightly more complicated on second glance. That concerns intricacies in the operation as well as pitfalls in interpretation. This text is meant to give practitioners a quick start, still going to beyond the simple Sauerbrey picture. The concepts underlying the more advanced models are important when carrying the QCM to non-standard applications (such as the freezing of droplets or the impact of spheres). 

The following list summarizes a few applications. (The list anticipates the later text in so far, as the topics and problems addressed have not been explained yet, but will be.) The numbers of citations are returns from a search in the Web of Science, where the dates were limited to January 2019–April 2021. A total of 1067 entries contain the keyword “QCM”. “QCM-D” returns 419 entries. “QCM-D” here denotes all instruments reporting frequency as well as bandwidth (or, equivalently, the dissipation factor) on a number of different overtones. In the majority of these publications, the QCM is one out of a few different instruments employed to study the respective samples. The QCM in these studies is a routine device. 

Topics of particular relevance with regard to modeling are:

- Numerous publications discuss the mass uptake of nanoporous and other rigid layers when exposed to a vapor of the analyte [7]. The porous layer takes the role of the receptor. The limit of detection of the QCM easily suffices for sensing building on this principle. (It does not easily suffice for similar sensors, building on adsorption to a planar surface.) These rigid structures swell and soften less than the polymer films, which took a similar role in the past [8]. While the emphasis in these works is on gravimetry, an analysis taking viscoelasticity into account (Equation (46)) will provide for more in-depth information. Also, it will yield a more accurate value for the mass uptake than the Sauerbrey equation.
- The search term “EQCM” returns 137 citations. These are increasingly concerned with an analysis beyond gravimetry. The non-gravimetric effects in this context mostly originate from roughness (Equation (77)), from the viscoelasticity of the double layer (Equation (59)), and from the softness of an active polymer layer (if present, Equation (46)).
- The keyword “QCM-D and brush” returns 37 entries. The brushes often undergo swelling/deswelling transitions or show electroresponsivity. Brushes should be modeled taking viscoelastic effects into account. The shear modulus varies between the bottom and the top, which necessitates the use of a viscoelastic profile (Equation (60)).
- 114 publications are returned for “QCM and particles”. The interpretation of such QCM data is a topic of ongoing research (Section 6.1 and Section 6.3). For instance, the amount of liquid mass contributing to the gravimetric signal (the “trapped mass”) usually is not known, quantitatively. A few publications explicitly refer to the positive frequency shift induced by sufficiently large particles (Equation (70)).
- 69 publications mention bacteria, which often implies bacterial adsorption (reviewed in [9]). In these cases (and also for cell cultures and biofilms) the shear wave often does not reach to the top of the layer. The QCM then cannot measure the thickness. If such a thick sample is homogeneous in viscoelastic terms, the QCM reports the shear modulus of this medium (Equation (31)). For reviews of applications in the life sciences, in general, see [10,11].
- Interestingly, 247 hits are returned when asking for “QCM and protein”. Protein adsorption is also routinely and successfully probed with optical techniques such as surface plasmon resonance (SPR) spectroscopy. The added information contained in the layer’s viscoelasticity (Equation (52)) is a distinctive advantage of the QCM.
- On the conceptual side, high-frequency rheology on polymers receives considerable attention. Some of these publications are returned when the keyword is “tribology” or “viscoelasticity”. A recent review is contained in [12]. The equations applied for analysis mostly are similar to what is described in Section 4.5.3 and Section 4.6.2. For thick films (microns), Equation (41) is a suitable fit function. Because the frequency shifts are large, temperature effects are irrelevant. For thin films (tens of nanometers), Equation (46) is more suitable than Equation (41). One can hope for data from more than 10 overtones available for analysis. However, the frequency shifts are smaller, which makes the analysis more susceptible to artifacts, for instance caused by changes of temperature.
- Rather few (<10) publications mention large amplitudes and nonlinear behavior. While this is an interesting field in the authors’ opinion, it has not been explored much, yet.

Some recent reviews (such as [11]) cover acoustic sensors other than the QCM (SAW devices, FBARs, nanoresonators, …). These devices operate at higher frequency than the QCM, which implies improved sensitivity in gravimetry. Most of them are smaller than the QCM. Some models of the QCM can be transferred to the smaller devices, but vibration in a sufficiently clean thickness-shear mode is often in question.

**Figure 1:**
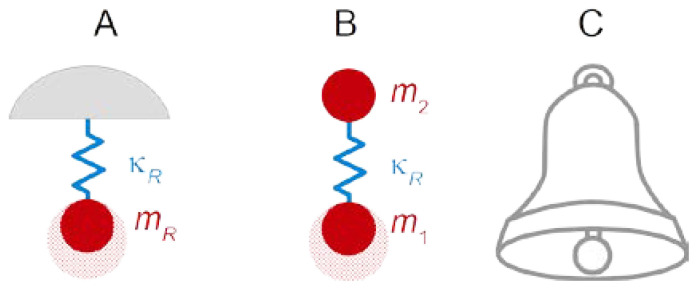
(**A**) The simplest possible harmonic resonator. A mass is linked to a rigid wall across a spring. The resonance frequency is $\omega_{0}\;\approx \;{\left(\kappa_{R}{/m}_{R}\right)}^{1/2}$. If $m_{R}$ increases, the resonance frequency decreases in consequence. (**B**) A resonator abandoning the rigid wall. Two masses are linked across a spring. The resonance frequency is given as $\omega_{0}\;\approx \;{\left(\kappa_{R}/µ\right)}^{1/2}$, where µ is the reduced mass (µ = $m_{1}m_{2}$ /($m_{1}$ + $m_{2}$)). Again, increasing one of the two masses will lower the resonance frequency. The diagram in (**B**) contains discrete elements, similar to the equivalent lumped-element circuits discussed in Section 8.4. (**C**) Contrasting to the resonators in (**A**,**B**), the bell does not consist of discrete masses and springs. It is an elastic body with a certain shape, made from materials with a certain density and stiffness. Finding its resonance frequencies (plural) is a classical problem of acoustics. For any given resonance, one may construct an equivalent lumped-element model containing discrete elements (as in (**A**) or (**B**)), which reproduces this one resonance. In principle, one might tune the bell by gluing weights to its rim. The common practice rather is to remove metal in annular rings, usually from the inside [14]. That changes both the effective mass and the effective spring constant. The focus then is not usually on the absolute frequency of the fundamental mode, but rather on the ratios between the overtone frequencies and the fundamental frequency. These ratios govern the perception of the bell’s sound.

The quartz crystal microbalance is about 60 years old. At that time, people working with quartz resonators knew that one can lower the frequency by scribbling onto the plate with a pencil. Günter Sauerbrey turned this practice into an equation and an instrument [13]. The frequency decreases, following the relation $\omega_{0}\;\approx \;{\left(\kappa_{R}{/m}_{R}\right)}^{1/2}$ with $\kappa_{R}$ some effective stiffness and $m_{R}$ some effective mass. By making the crystal heavier, one slows down its resonant vibration (Figure 1). 

Sauerbrey realized that the relation between mass and frequency shift is particularly simple if, firstly, the resonator is a plate oscillating in the thickness-shear mode and if, secondly, the sample consists of a thin film. Most resonators at this time indeed were thickness-shear resonators. Thin films were routinely coated onto these as electrodes. This insight allowed to develop film-thickness monitors for deposition processes of various kinds. 

A side remark: Plates of α-quartz may resonate in the thickness-shear mode if the crystallographic *x*-axis is in the surface plane. There are certain angles between the crystallographic *y*-axis and the surface normal, at which the temperature-frequency coupling at room temperature almost vanishes. One of these temperature-compensated cuts is the widely used AT cut.

Sauerbrey’s famous formula is:

(1) $$\frac{\Delta f}{f_{\mathrm{ref}}}=-\frac{m_{f}}{m_{q}}\;\;$$

Δ*f*/$f_{\mathrm{ref}}$ is the fractional frequency shift. $m_{f}$ and $m_{q}$ are the mass per unit area of the film and the resonator, respectively. One might also talk about “mass” rather than “mass per unit area”, but the latter term is more practical. For instance, the mass per unit area is easily converted to thickness, if the density is known. The resonator’s mass per unit area, $m_{q}$, may be replaced by $Z_{q}$/(2*n*$f_{0}$), where $Z_{q}$ = 8.8 × 10^6^ kg/(m^2^s) is the resonator’s shear-wave impedance, $f_{0}$ is the frequency of the fundamental (often 5 MHz), and *n* is the overtone order. These relations inserted into Equation (1) lead to:

(2) $$\Delta f=-\frac{{2nf}_{0}^{2}}{Z_{q}}m_{f}\;.$$

**Figure 2:**
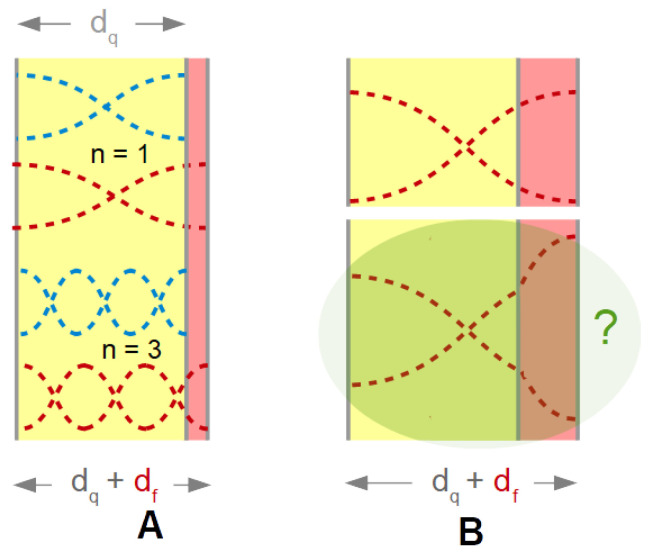
(**A**) A film increases the wavelength of the standing wave, thereby decreasing the resonance frequency. (**B**) If the film is softer than the plate, the displacement pattern has a kink at the interface. In this case, the fractional frequency shift is proportional to the fractional increase in mass (rather than thickness).

The reasoning behind the Sauerbrey equation is sketched in Figure 2. The interesting vibration modes are standing transverse waves with antinodes at the surfaces. Figure 2 shows the displacement patterns of the fundamental mode and the 3rd overtone as dashed lines. (An “overtone” here is an eigenmode, that is, a solution to the boundary value problem, not to be confused with second-harmonic generation or third-harmonic generation. The latter terminology is also common in acoustics.) The wavelength is 2$d_{q}$/*n* with $d_{q}$ the thickness of the plate and *n* the number of nodal planes. Only odd overtones (*n* = 1, 3, 5, …) can be excited piezoelectrically, because the even overtones lead to a surface charge with the same sign on both sides. It is customary to label the overtones (the “overtone order”) with indices equal to the number of nodal planes. The frequency is $f_{\mathrm{res}}$ = *n*$c_{q}$/(2$d_{q}$) with $c_{q}$ the speed of sound. 

The Sauerbrey mass, $m_{f}$, is often quoted in units of µg/cm^2^. More intuitive would be a thickness (the “Sauerbrey thickness”), the calculation of which, however, requires knowledge of the density. With 5 MHz crystals and a density of 1 g/cm^3^, a 1 nm film shifts the frequency by Δ*f*/*n* = −5.7 Hz. With this density, 1 µg/cm^2^ corresponds to 10 nm.

Consider a film with the exact same acoustic properties as the resonator itself (top in Figure 2B). In acoustic terms, the film makes the plate thicker. The wavelength increases correspondingly and the frequency decreases. If the film is much thinner than the plate, one might expect the relation $\Delta f/f_{\mathrm{ref}}\approx {\;-d}_{f}/d_{q}$. This is *not* the Sauerbrey equation. The Sauerbrey equation makes a statement about mass, not about thickness. Above, the film was assumed to have the same acoustic properties as the plate (same density, ρ, same shear modulus, *G*). If this is not the case, the displacement pattern has a kink at the resonator-film interface (bottom in Figure 2B). Sauerbrey went through the mathematics and realized that the fractional thickness must be replaced by the fractional mass in order to let the relation be applicable to arbitrary materials.

Assume a resonator with a fundamental frequency of 5 MHz. Further, assume that the frequency can be determined with a precision of 0.5 Hz. This precision in frequency translates to a precision in mass of about 10 ng/cm^2^. Because the unit has the prefix “nano”, people have advocated the term “quartz crystal nanobalance”. In the end, the “quartz crystal microbalance” became the accepted term. With a density of about 1 g/cm^3^, the thickness resolution of this QCM is about 0.1 nm. The QCM has “submonolayer sensitivity”. Monomolecular layers of typical bio-adsorbates are slightly thicker than 1 nm. The QCM has submonolayer sensitivity, but the sensitivity is not deep in the submonolayer range. In order to study the kinetics of adsorption in detail, one would wish for an even better limit of detection (LOD, Section 3.6). The LOD of the QCM is good, but not strictly fantastic. 

Two competitors are worth a mention. For gas sensing, the surface acoustic wave devices (SAW devices, [15]) have a better LOD. These are used in some electronic noses. For label-free biosensing in liquids, surface plasmon resonance spectroscopy (SPR spectroscopy [16]) also has an LOD better than the QCM. It is a question of both white noise and drift. Among the reasons to use the QCM is simplicity. Other reasons are connected to the depth of information. The QCM gives access to physical parameters beyond the mass per unit area. This is sometimes emphasized by calling the QCM an “instrument of surface analysis”, rather than a “sensor”.

Gravimetry in air or vacuum was the QCM’s main use until the early 1980s [17]. At that time, Nomura in [18,19] and also Bruckenstein and Shay in [20] combined a QCM with an electrochemical cell and measured the mass transfer during electrodeposition. Attempts into that direction were made earlier but were less successful [21]. The first experiments with this “electrochemical QCM” (EQCM) were analyzed with the Sauerbrey equation. As was shown later, the Sauerbrey equation also applies in liquids as long as the layer is rigid (Equation (52), [22]). Of course, the liquid itself also has an influence. This influence is described by what today is called the Gordon-Kanazawa equation [23]. The Gordon-Kanazawa equation is a rediscovery. In slightly implicit form, it is, for instance, mentioned in [24]. Quite generally, a considerable body of experience on acoustic resonators in liquids was gathered in the 1930s to 1950s, using torsional resonators. Part of this knowledge is collected in Mason’s book from 1948 [25]. The concepts, which underlie our current understanding of the liquid-phase QCM, mostly date from that period. 

Later, there were two more additions to the techniques. Firstly, the resonance bandwidth was analyzed in addition to the resonance frequency [26] and the shifts of frequency and bandwidth were compared between overtones. Secondly, the oscillator circuits were largely replaced by passive interrogation (impedance analysis [27,28] and ring-down [29,30,31]). In recent years, the cost of impedance analyzers has come down [32]. These changes lead to the “advanced QCM”. Another term is “QCM-D” for “QCM with Dissipation monitoring”. “QCM-D” here is the name of a technique, not of one particular instrument. In this text, QCM is synonymous to QCM-D.

## 2. Forced Vibrations, Complex Resonance Frequencies

The following section motivates the complex frequency shift, $\Delta \tilde{f}=\Delta f+i\Delta \Gamma$ [33]. The variable Γ denotes the half bandwidth at half height (“bandwidth” for short). The tilde denotes a complex parameter.

Start from the equation of motion of the forced resonator:

(3) $$m_{R}\ddot{x}\left(t\right)=-\xi_{R}\dot{x}\left(t\right)-\kappa_{R}x\left(t\right)+F_{\mathrm{ext}}\left(t\right)$$

$m_{R}$ is the mass. $\xi_{R}$ is the friction coefficient, also called “drag coefficient”. In interfacial sliding, the “friction coefficient” is a ratio of two forces (tangential to normal). In liquid friction, it is a ratio of force to velocity. Renaming the force-velocity ratio as “drag coefficient” avoids this ambiguity. $\kappa_{R}$ in Equation (3) is the spring constant. 

We bring all terms containing *x*(*t*) to the left-hand side. The source term (the external force, $F_{\mathrm{ext}}$) shall be of the form ${\hat{F}}_{\mathrm{ext}}\;\exp\left(i\omega t\right)$. The hat (^) denotes a complex amplitude. Instead of exp(iω*t*), one might have also written exp(−iω*t*). That is a matter of convention, addressed in Box 1. For stationary oscillations of the form $x(t)=\hat{x}\exp(i\omega t)$, the time derivative turns into a multiplication with iω:

(4) $$-\omega^{2}m_{R}\hat{x}\exp(i\omega t)+i\omega \xi_{R}\hat{x}\exp(i\omega t)+\kappa_{R}\hat{x}\exp(i\omega t)={\hat{F}}_{\mathrm{ext}}\exp(i\omega t)$$

We divide by exp(iω*t*), divide by $m_{R}$, rename $\xi_{R}$/$m_{R}$ as 2γ, and rename $\kappa_{R}$/$m_{R}$ as ${\;\omega }_{0}^{2}$:

(5) $$-\omega^{2}\hat{x}+2i\omega \gamma \hat{x}+\omega_{0}^{2}\hat{x}=\frac{{\hat{F}}_{\mathrm{ext}}}{m_{R}}$$

γ is the damping coefficient and $\omega_{0}$ is the natural frequency. Both have units of inverse seconds. The amplitude of displacement depends on ω as:

(6) $$\hat{x}\;=\;\frac{1}{\omega_{0}^{2}\;-{\;\omega }^{2}\;+\;2i\gamma \omega }\cdot \frac{{\hat{F}}_{\mathrm{ext}}}{m_{R}}\;$$

Box 1 **Sign conventions.**

When describing oscillations with complex numbers, one exploits Euler’s relations, which imply that cos(ω*t*) = 1/2(exp(iω*t*) + exp(−iω*t*)). In principle, all calculations containing the cosine should be carried out on the sum of exp(iω*t*) and exp(−iω*t*). However, the two calculations with +iω*t* and with –iω*t* run in similar ways. One therefore carries out the calculation just once and eventually computes the (real) outcome of the calculation as Re($\tilde{y}$) = 1/2 ($\tilde{y}$ + $\tilde{y}$*) where $\tilde{y}$ is the outcome of the calculation for exp(iω*t*) and the asterisk denotes complex conjugation.

If entropy is supposed to always increase, the imaginary parts of certain complex response functions must have certain signs. The sign depends on whether the calculation is carried out with exp(iω*t*) or with exp(−iω*t*). If exp(iω*t*) is chosen, the signs are:
$\tilde{G}=G^{\prime }+iG^{\prime \prime }$${\hat{\sigma }}_{\mathrm{shear}\;}=\tilde{G}{\hat{\gamma }}_{\mathrm{shear}\;}$shear modulus σ_shear_: stress γ_shear_: strain$\tilde{\eta }=\eta^{\prime }-i\eta^{\prime \prime }$$\tilde{G}=i\omega \tilde{\eta }$viscosity$\tilde{J}=J^{\prime }-\dot{1}J^{\prime \prime }$$\tilde{J}=1/\tilde{G}$shear compliance$\tilde{c}=c^{\prime }+ic^{\prime \prime }$$\tilde{c}={\left(\tilde{G}/\rho \right)}^{1/2}={\left(i\omega \tilde{n}/\rho \right)}^{1/2}$speed of shear sound$\tilde{k}=k^{\prime }-ik^{\prime \prime }$$\tilde{k}=\omega /\tilde{c}$wave number, wave travels towards +*z*$\tilde{Z}=Z+iZ^{\prime \prime }$$\tilde{Z}=\rho \tilde{c}={\left(\rho \tilde{G}\right)}^{1/2}={\left(i\omega \rho \tilde{\eta }\right)}^{1/2}$wave impedance$\begin{array}{cc} {\tilde{\omega }}_{\mathrm{res}} & =\omega_{0}+i\gamma =2\pi \left(f_{\mathrm{res}}+i\Gamma \right)\\ & =2\pi \left(f_{\mathrm{res}}+if_{\mathrm{res}}D/2\right) \end{array}$
resonance frequency
ω is real

A wave propagating towards +*z* is written as exp(i(ω*t* – *k*$\tilde{z}$)) = exp(iω*t*) exp(−i*k*′*z*) exp(−*k*′′*z*).

Because the resonances of the QCM are extraordinarily sharp, the frequency of excitation, ω, is close to the natural frequency, $\omega_{0}$.

A side remark: For sharp resonances, the frequency of maximum displacement is the natural frequency. The natural frequency is called the “resonance frequency”, here. For broad resonances, there is a slight difference between the natural frequency and the resonance frequency. The latter then is also called the “ringing frequency”, equal to ω_0_(1 − 2γ^2^/ω_0_^2^)^1/2^. One can always compute the ringing frequency from the natural frequency and the bandwidth. The difference is not of practical importance for the QCM.

If ${\omega \approx \omega }_{0}$, the denominator can be simplified following $\left(\omega_{0}^{2}\;-\omega^{2}\right){\approx (\omega }_{0}+\omega )\left(\omega_{0}\;-\;\omega \right)\approx {\;2\omega }_{0}\left(\omega_{0}\;-\;\omega \right).$ Equation (6) simplifies to:

(7) $$\hat{x}(\omega )=\frac{1}{\omega_{0}^{2}-\omega^{2}+i2\gamma \omega }\cdot \frac{{\hat{F}}_{\mathrm{ext}}}{m_{R}}\approx \frac{1}{\left(\omega_{0}-\omega \right)+i\gamma }\cdot \frac{{\hat{F}}_{\mathrm{ext}}}{2\omega_{0}m_{R}}$$

A complex resonance frequency can be defined as:

(8) $${\tilde{f}}_{\mathrm{res}\;}=\frac{\omega_{0}+i\gamma }{2\pi }=f_{\mathrm{res}\;}+i\Gamma$$

where Γ = γ/(2π) is the half bandwidth at half height (the complex resonance frequency makes the algebra easier *if* the resonances are sharp and if ω + ω_0_ ≈ 2ω_0_. Otherwise, it can cause confusion).

Expressed in terms of the complex resonance frequency, Equation (7) turns into:

(9) $$\hat{x}(f)\approx \frac{{\hat{F}}_{\mathrm{ext}\;}}{8\pi^{2}f_{\mathrm{res}\;}m_{R}}\frac{1}{{\tilde{f}}_{\mathrm{res}\;}-f}$$

The prefactor is often multiplied with an i and then hidden behind some normalization constant. Proceeding this way and separating the real and the imaginary part leads to:

(10) $$\hat{x}\left(f\right)\;\propto \;\frac{\Gamma }{{\left({f\;-\;f}_{\mathrm{res}}\right)}^{2}{\;+\;\Gamma }^{2}}\;+\;i\frac{{f\;-\;f}_{\mathrm{res}}}{{\left(f\;-{\;f}_{\mathrm{res}}\right)}^{2}{\;+\;\Gamma }^{2}}\frac{{f\;-\;f}_{\mathrm{res}}}{{\left(f\;-{\;f}_{\mathrm{res}}\right)}^{2}{\;+\;\Gamma }^{2}}\;$$

**Figure 3:**
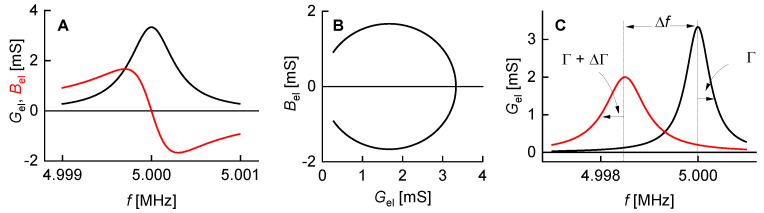
A typical output from impedance analysis. Panel (**A**) shows the conductance $G_{\mathrm{el}}$ (black) and the susceptance, $B_{\mathrm{el}}$ (red). Together, they form the complex electrical admittance, ${\tilde{Y}}_{\mathrm{el}}$ = $G_{\mathrm{el}}$ + i$B_{\mathrm{el}}$, which is equal to ${\tilde{Z}}_{\mathrm{el}}^{-1}$ with ${\tilde{Z}}_{\mathrm{el}}$ the impedance. The real part of the admittance forms the well-known, symmetric resonance curve (assuming perfect calibration). This is different for the real part of ${\tilde{Z}}_{\mathrm{el}}$ because of the parallel electrical capacitance, $C_{0}$. *G*_el_(*f*) peaks at the series resonance frequency, $f_{\mathrm{res}}$. Panel (**B**) shows the polar diagram. Of interest in sensing are the *shifts* in frequency and bandwidth, Δ*f* and ΔΓ (**C**).

The first and the second term are shown as a black and a red line in Figure 3.

The complex resonance frequency plays out its strength when it comes to shifts thereof, called Δ$\tilde{f}\;$ in the following (Δ$\tilde{f}\;$ = Δ*f* + iΔΓ). The complex shift was proposed by Eggers and Funk [33]. Just about all equations predicting frequency and bandwidth can be formulated in terms of Δ$\tilde{f}\;$. These equations cover Δ*f* and ΔΓ at the same time. 

The half bandwidth, Γ, is related to the energy dissipated per unit time, $\dot{E}$, as:

(11) $$\Gamma =\frac{\dot{E}}{4\pi E}$$

*E* is the energy contained in the oscillation. 

In the authors’ opinion, Γ is the best parameter for quantification of dissipative processes at the QCM surface. Γ puts frequency and bandwidth on equal grounds. For instance, the noise on Δ*f* and ΔΓ is similar. Other parameters are in use. Some researchers use the full bandwidth, *w* = 2Γ, others use the Q-factor *Q* = $f_{\mathrm{res}}$/(2Γ), and still others use the inverse Q-factor *Q*^−1^ = 2Γ/$f_{\mathrm{res}}$ and give it a new name and a new letter, namely “dissipation factor”, *D*. Sometimes the “dissipation factor” is called “dissipation”, for short. $\Delta \tilde{f}$ may also be expressed in terms of the dissipation factor. The conversion is simplest for the overtone-normalized frequency shift:

(12) $$\frac{\Delta \tilde{f}}{n}=\frac{\Delta f}{n}+i\frac{\Delta \Gamma }{n}=\frac{\Delta f}{n}+i\frac{f_{0}}{2}\Delta D$$

If Δ*D* is expressed in units of 10^−6^ and if $f_{0}$ is 5 MHz, the conversion from Δ*D* [10^−6^] to ΔΓ/*n* [Hz] amounts to a multiplication with 2.5. 

## 3. Techniques of Read-Out

The methods of interrogation all rely on piezoelectricity and the plate’s electrical impedance, ${\tilde{Z}}_{\mathrm{el}}$(ω), in the respective frequency range. ${\tilde{Z}}_{\mathrm{el}}$(ω) (and, also, the admittance, ${\tilde{Y}}_{\mathrm{el}}$(ω) = 1/${\tilde{Z}}_{\mathrm{el}}$(ω)) form a resonance curve. The interrogation methods are sometimes grouped into “active” and “passive”. In the active schemes, the resonator is part of an oscillator circuit. The amplifier contained in this circuit takes a certain influence on the oscillation frequency. The other schemes are passive. Passive, however, does not mean that the apparatus would not take an influence on the resonance parameters, at all. (Even for the “grandfather clock” [34], the way of driving slightly affects the frequency.) For piezoelectric resonators, this influence is mediated by piezoelectric stiffening (Section 7). The stiffness of a piezoelectric plate (and hence its resonance frequency) depends on whether the two electrodes are open, short-circuited, or connected across some electrical impedance, ${\tilde{Z}}_{\mathrm{ext}}$. The latter situation is realized in all electrical instrumentation controlling the resonator. Again, $f_{\mathrm{res}}$ depends on ${\tilde{Z}}_{\mathrm{ext}}$. Calculating the impedance, which the crystal “sees”, is nontrivial. 

The different modes of interrogation differ from each other in cost, speed, and susceptibly to artifacts. They do not, actually, differ much from each other in precision (Section 3.6). References [35,36,37] cover the interface electronics in more detail. 

### 3.1. Oscillator Circuits

An oscillator circuit is an amplifier with a resonator in the feedback loop. Because the resonator’s impedance is small on the resonance frequency, the circuit spontaneously oscillates at this frequency. Oscillator circuits are the method of choice for clocks [38]. QCMs based on oscillators can be cheap, even after the frequency counter is included in the total cost. For advanced sensing, oscillators are problematic because the frequency of oscillation is not strictly equal to the frequency at the peak of the conductance curve. The latter frequency (the acoustic resonance frequency, also “series resonance frequency”) is the frequency of relevance for interpretation. The parallel electrical capacitance ($C_{0}$, Section 8.4) takes an influence on the oscillation frequency. There are more intricacies in the details. These would not be a problem, if the small difference between the oscillation frequency and the series resonance frequency was constant, but this difference depends on damping and on details of the electronics. Oscillator circuits are available, which output bandwidth in addition to frequency (bandwidth being often converted to the dissipation factor) [39,40]. Oscillator circuits usually run on one harmonic only, often the fundamental.

### 3.2. Impedance Analysis

Impedance analysis [27] avoids the complications inherent to oscillator circuits. An impedance analyzer (synonymous to “vector network analyzer”, “VNA”) sweeps the frequency of excitation across the resonance. The resonance parameters are obtained from a fit of a resonance curve to the admittance trace. A suitable fit function is the phase-shifted Lorentzian, which is:

(13) $$G_{\mathrm{fit}}{=G}_{\max}\Gamma \left(\frac{\Gamma }{{\left(f_{\mathrm{res}}-\;f\right)}^{2}{+\Gamma }^{2}}\cos\varphi +\frac{f_{\mathrm{res}}-\;f}{{\left(f_{\mathrm{res}}-\;f\right)}^{2}{+\Gamma }^{2}}\sin\varphi \right){+G}_{\mathrm{off}}\;B_{\mathrm{fit}}{=G}_{\max}\Gamma \left(\frac{\Gamma }{{\left(f_{\mathrm{res}}-\;f\right)}^{2}{+\Gamma }^{2}}\sin\varphi +\frac{f_{\mathrm{res}}-\;f}{{\left(f_{\mathrm{res}}-\;f\right)}^{2}{+\Gamma }^{2}}\cos\varphi \right){+B}_{\mathrm{off}}\;$$

The phase shift in Equation (13), φ, accounts for an asymmetry of the resonance curve. Imperfect calibration causes such an asymmetry. The asymmetry can be small, but it rarely vanishes. $G_{\max}$ is an amplitude. The parameter $G_{\max}$ does not contribute much to sensing. The product $G_{\max}\Gamma$ is proportional to the effective area of the plate (Equation (113)). $G_{\max}\Gamma$ sometimes varies slightly during experiment. How these variations depend on the sample’s properties, is poorly understood

Impedance analysis is among the passive techniques. “Passive”, however, does not imply that the impedance analyzer would not affect the resonance frequency, at all. The analyzer’s output resistance, its input resistance, and the length of the cables all take an influence on frequency and bandwidth because of piezoelectric stiffening. A second caveat: The resonance frequency as determined from the admittance trace depends on the sweep rate. Impedance analysis is not quite as reliable as one would wish. Still: impedance analysis is rather transparent. The problems are noticed and their consequences can be quantified with moderate effort.

**Figure 4:**
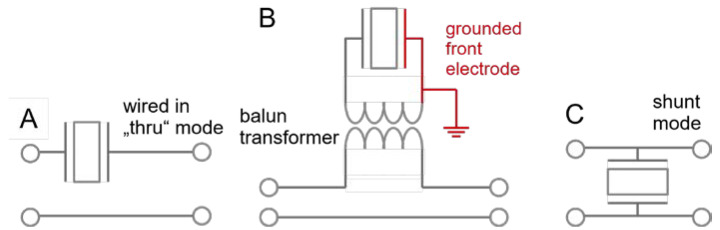
When working in liquids, wiring the resonator in the thru configuration (**A**) lowers the noise. A balun transformer ((**B**), such as the unit ADT1-1 from Minicircuits) can be used to ground the front electrode. The shunt configuration (**C**) is not recommended for use in liquids.

For measurements in liquids, the through (“thru”) configuration is advantageous because it leads to a small current into the impedance analyzer. The small current is measured against zero background and may by amplified. The background is nonzero in the “shunt” configuration, which is also common and works well for experiments in air. In the shunt configuration (depicted in Figure 4C), a large impedance of the device under test lets the voltage from the output go straight to the input of the VNA. If the resonator’s impedance is much larger than 50 Ω, it causes small changes to this input against a large background. Because the background is amplified as well, amplification lets the detector run into overload. A resonator immersed in a liquid has a large impedance on resonance and should be wired in thru configuration. If grounding the front electrode is an issue, a transformer as shown in Figure 4B can be employed. Grounding the front electrode is advisable because the electrical properties of the sample may otherwise affect the resonance via piezoelectric stiffening.

### 3.3. Ring-Down

**Figure 5:**
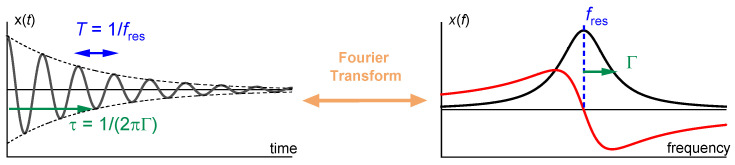
The time trace in ring-down and the resonance curve as a function of frequency are related to each other by a Fourier transform. The resonance parameters (mostly $f_{\mathrm{res}}$ and Γ or *D*) can be obtained from both sets of data. The precision is similar.

Resonant phenomena can always be probed in either the frequency domain or the time domain. As long as the dynamical equations are linear, the two modes of interrogation yield equivalent information (Figure 5). One may either sweep the frequency of an AC excitation across the resonance (as in impedance analysis) or abruptly shut off the driving signal and watch the decay as a current trace on an oscilloscope (as in ring-down). The latter principle is implemented in the instrument marketed by Biolin Scientific (Västra Frölunda, Sweden). 

### 3.4. Multi-Frequency Lock-In Amplification

**Figure 6:**
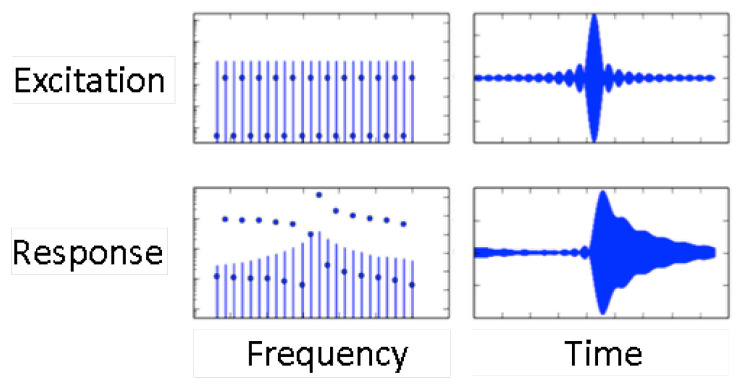
Excitation and response of a resonator in the frequency domain and the time domain as determined with the MLA. The instrument applies a comb of up to 32 frequencies. The current response shows the resonance curve (**bottom left**). Transformed to the time domain (**right**), the excitation amounts to a sequence of pulses with a spacing in time of 1/(Δ*f*_comb_). The response is similar to a ring-down process (**lower right**). Downloaded from www.intermodulation-products.com/applications/lock-in, accessed on 13 February 2019.

The multi-frequency lock-in amplifier (MLA) stands between ring-down and impedance analysis. The MLA applies a comb of frequencies to the resonator. The resonance curve can be reconstructed from the signals returned to the MLA at these frequencies. The raw data are time-domain data, which are Fourier-transformed on the instrument’s main board. In the time domain, the excitation amounts to a series of pulses. The current as displayed on an oscilloscope (lower right in Figure 6) visualizes the ring-down. The left-hand side and the right-hand side in Figure 6 describe the same process in the frequency domain and the time domain, respectively.

The time between two pulses, Δ*t*_comb_, sets the time resolution. Δ*t*_comb_ is equal to the inverse frequency spacing between two members of the comb, Δ*f*_comb_. The frequency spacing, in turn, must be smaller than the bandwidth of the resonance. Otherwise, the comb will miss the resonance. Using 32 frequencies, which are evenly distributed over a resonance with a width of about 3 kHz, one achieves a time resolution of 10 ms. However, one may also let the comb consist of only five frequencies and space those 500 Hz apart. The time resolution then improves to 2 ms. 

Should a comb covering one particular resonance contain only, for example, 5 frequencies, the other 27 frequencies can be invested in the other overtones. The MLA can interrogate multiple overtones at the same time. In principle, one might worry about crosstalk between the different overtones excited in parallel, but this does not appear to be a problem, in practice [41]. 

### 3.5. Fast Measurements, Modulation Experiments

Data acquisition rate is critical for the study of transient phenomena. It is particularly important in analytical electrochemistry, which often exploits transients [42]. Most current advanced QCMs are not particularly powerful in terms of time resolution. Typical data acquisition rates are between 1 and 10 data points per second (for experiments in liquids) [43]. While the MLA in the comb mode is faster than most other instruments, one would still wish for more. 

**Figure 7:**
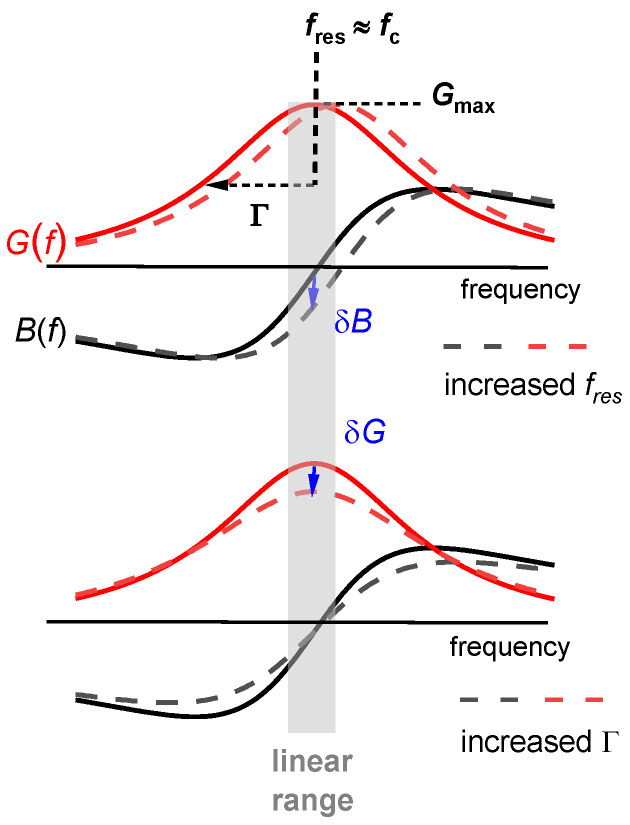
Impedance analysis requires a determination of the entire admittance trace (full and dashed lines), which takes time. Fast measurements can build on the conductance, $G_{\mathrm{el}}$, and the susceptance, $B_{\mathrm{el}}$, at one single frequency close to the center of the resonance, $f_{c}$. Shifts in $G_{\mathrm{el}}$ and $B_{\mathrm{el}}$ are converted to shifts in $f_{\mathrm{res}}$ and Γ (blue arrows). As the top panel shows, a shift in resonance frequency changes $B_{\mathrm{el}}$, but leaves $G_{\mathrm{el}}$ unchanged. Conversely, an increase in bandwidth lowers $G_{\mathrm{el}}$, but leaves $B_{\mathrm{el}}$ unchanged (bottom).

The data acquisition rate can be further improved, if the analysis is based on the electrical admittance at one, fixed frequency [44,45,46,47,48]. Following [47], we call this mode the “fixed-frequency-drive” (FFD) mode. As sketched in Figure 7, there is a one-to-one correspondence between the electrical admittance at this one frequency and the complex resonance frequency, $f_{\mathrm{res}}$ + iΓ. This mode of data acquisition suffers from electrical artifacts, though. The conversion from $G_{\mathrm{el}}$ + i$B_{\mathrm{el}}$ to $f_{\mathrm{res}}$ + iΓ assumes that the other parameters of the fit function in Equation (13) (given as $G_{\max}$, φ, $G_{\mathrm{off}}$, and $B_{\mathrm{off}}$) are constant, which is not always the case. 

Even in the fixed-frequency-drive mode, it is difficult to achieve data acquisition rates beyond 2πΓ because the resonator remembers previous resonance conditions on a time scale of ${\left(2\pi \Gamma \right)}^{-1}$. The memory is related to ringing up and ringing down after the resonator is turned on or off. The details are complicated. They can be studied with a varicap diode wired in series with the resonator. Switching the capacitance of the diode changes the stiffness of the quartz plate (piezoelectric stiffening, Section 7), thereby rapidly switching the resonator’s natural frequency. The resonance frequency as determined from impedance analysis follows with delay. Deconvolution of experimental data with a memory kernel should be possible but has not been done so far. Without deconvolution, the time per data point cannot be less than about 100 µs (for experiments in water, where Γ is a few kHz [47,48]).

Fast measurements may be combined with accumulation and averaging. This requires an experimental setting, where the sample responds to a periodic stimulus of some kind. Among others, a suitable parameter for modulation is the electrical DC potential of the front electrode when this electrode at the same time is the working electrode of an electrochemical setup. The instrument then operates as an electrochemical QCM (an EQCM, Section 9.1). 

**Figure 8:**
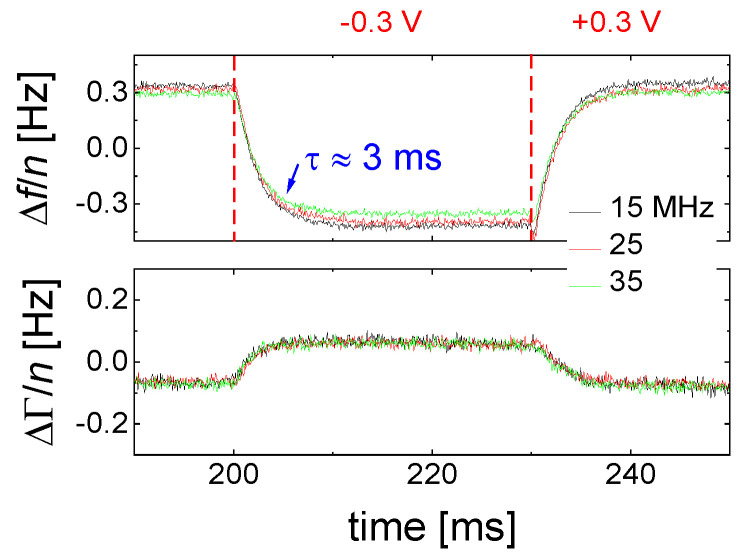
Shifts in frequency and bandwidth obtained in a modulation experiment, using the fixed-frequency-drive mode (FFD mode). The stimulus was the electrical potential of the front electrode, varied between +0.3 V and −0.3 V vs. a platinum pseudo-reference electrode. The sample was an electrochemically inert electrolyte. After accumulation overnight, the noise was around 20 mHz, based on a time interval of 100 µs per data point.

Figure 8 shows an example. The sample is an aqueous electrolyte. When the voltage of the front electrode is switched, Δ*f* and ΔΓ respond, but they do so with a delay. The delay is linked to the kinetics of double layer recharging [48].

Modulation and accumulation avoid a critical problem of the QCM, which is drift. Typical QCMs drift by about 1 Hz/h when the crystal was mounted carefully and when all static stresses have relaxed. Otherwise, the drift can be much larger. The drift is mostly caused by migration of crystal defects, possibly also by insufficient control of temperature. The drift can hardly be prevented, but it can be circumvented by choosing the target of research suitably. The study of fast, repetitive processes does not suffer from drift because the average (taken over the period of the repetitive process) can be subtracted from the time traces. The average will drift, but the difference from the average can be accumulated over extended periods of time. The data shown in Figure 8 have been accumulated overnight. As a side remark: Oscillators also allow for fast data acquisition, as demonstrated in [49].

### 3.6. Noise and Drift

According to the conventions in sensing, the limit of detection, LOD, is three times the rms noise. Noise is one of the reasons, why surface plasmon resonance spectroscopy (SPR spectroscopy) is more widespread in label-free biosensing than the QCM. A second reason is drift. Noise has in depth been studied for clocks [50], but not to the same extent for the liquid-phase QCM.

To the best of the authors’ knowledge, the different techniques and instruments driving the liquid-phase QCM reach a frequency noise in a similar range. A convenient way to calculate a drift-corrected noise from any data set builds on the Hadamard variance, which is:

(14) $${\delta f}_{\mathrm{Hadamard}}^{\;2}=\frac{1}{6}\langle {\left(f_{i-1}-{\;2f}_{i}{+f}_{i+1}\right)}^{2}\rangle {}_{i}\;$$

The Hadamard variance is zero for a straight, sloped line. After a linear fit is subtracted from a sloped line with added white noise, the root-mean-square noise (rms noise) of this data set is equal to the square root of the Hadamard variance of the original data set. Basing the definition of the noise on the Hadamard variance avoids the linear fit. The drift-corrected rms noise is ${\left({\delta f}_{\mathrm{Hadamard}}^{\;2}\right)}^{1/2}$.

In the authors’ laboratory, measurements in water lead to an rms noise on Δ*f*/*n* of about 30 mHz/Hz^1/2^. The noise depends on the time interval of data acquisition, hence the Hz^1/2^ in the denominator. One can always lower the noise by averaging over longer times (assuming white noise). 30 mHz is the noise, if the instrument outputs one data point per second. Similar noise is seen in most figures in the published literature (which is a rough estimate, evidently). Reference [51] reports similar noise for resonators in liquids driven with an oscillator circuit (as opposed to impedance analysis or ring-down). Using a density of 1 g/cm^3^, an LOD in frequency of 90 mHz (which is 3 × the rms-noise) corresponds to an LOD in adsorbate thickness of ~0.05 nm.

In time and frequency control, the Allan variance is employed more commonly than the Hadamard variance. When frequencies are determined in time intervals of τ = 1 s, the Allan variance is:

(15) $$\sigma_{y}^{\;2}\left(\tau =1\;s\right)=\frac{1}{2}\langle {\left(y_{i+1}-{\;y}_{i}\right)}^{2}\rangle$$

*y* is the fractional frequency shift. Good quartz clocks achieve $\sigma y(\tau =1\;s)\;\approx \;10^{-11}$ [52]. For the QCM in water, the noise is larger by a factor of about 1000. With a noise of 30 mHz on a 5 MHz signal, σ_y_(1 s) is 6 × 10^−9^. The Q-factor, on the other hand, decreases by only a factor of about 30. The noise is not proportional to *Q*^−1^, as one might expect.

**Figure 9:**
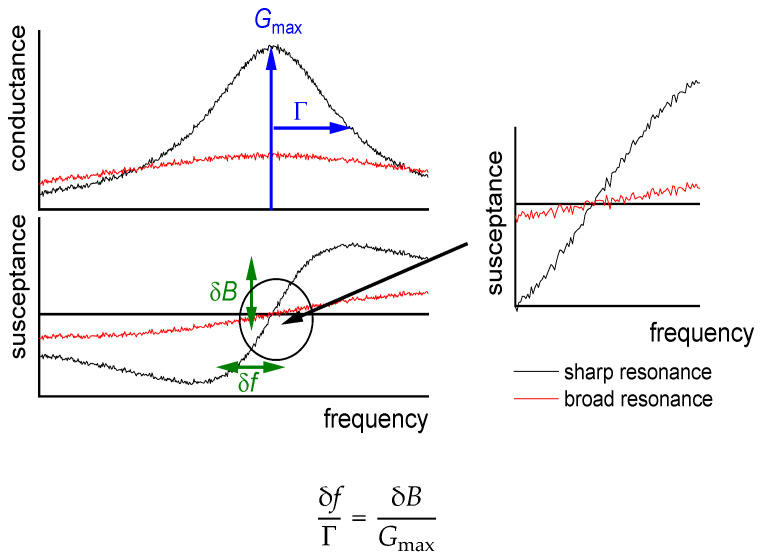
When a resonance becomes broader, this lowers the precision in the determination of the frequency shift twice. Γ increases and $G_{\max}$ decreases (both in proportion to the inverse Q-factor). The noise on δ*B* is assumed to be independent of *Q* in this argument.

One can understand that the frequency noise increases stronger than *Q*^−1^ on a qualitative level. The discussion can build on the fixed-frequency-drive mode (Section 3.5). In a liquid environment, the large damping increases Γ and it also decreases *G*_max_ (Equation (13), Figure 9). More generally, the noise has a white component, which scales as the ratio of the thermal energy, *k*_B_*T*, to the power going into the device. When immersing a resonator into a liquid, the power into the device decreases because the resistance ($R_{1}$) increases. This amounts to a first factor of about *Q*^−1^. A second factor of *Q*^−1^ enters, when a noise in ${\tilde{Y}}_{\mathrm{el}}$ is translated to a noise in frequency. With *Q* decreasing by about a factor of 30, the noise (following this rough argument) increases by about a factor of $30^{2}$.

There may be other sources of noise. For instance, the liquid-phase QCM is susceptible to acoustic vibrations. Slamming the door leaves a trace in Δ*f*(*t*). The coupling is mediated by bending of the plate as described in [53]. More generally: There probably is room for improvements on the frequency noise of the liquid-phase QCM.

## 4. The Acoustic Multilayer Formalism and Its Consequences

The following sections describe the quantitative analysis of QCM data acquired on planar samples in detail. As long as the samples are homogeneous in the surface plane, the acoustic multilayer formalism achieves the modeling. 

### 4.1. Qualitative Data Inspection

Before starting a fit, some qualitative considerations are worthwhile:

- Is −Δ*f* ≫ ΔΓ and is −Δ*f*/*n* ≈ const.? Did the experiment occur in air? If so, the response is probably dominated by inertia in the sense of the Sauerbrey equation (“inertial loading”). With a density of 1 g/cm^3^ and 5 MHz crystals, a layer thickness of 1 nm leads to −Δ*f*/*n* = 5.7 Hz. Did the experiment occur in liquid? If so, the response is probably dominated by the formation of a thin layer. However, −Δ*f*/*n* may be smaller than 5.7 Hz per nanometer in case the film is soft (Equation (52)).
- Is −Δ*f* ≈ ΔΓ, is −Δ*f*/*n*^1/2^ ≈ const., and was the resonator immersed in a liquid? If so, the response is probably dominated by changes in viscosity (Equation (29), “viscous loading”). With 5 MHz crystals, −Δ*f*/*n*^1/2^ = 716 Hz corresponds to a viscosity of 1 mPa s (slightly more than the viscosity of water).
- Is Δ*f* > 0 and is Δ*f*·*n* ≈ const.? If so, the response may be dominated by point contacts (“elastic loading”, Section 5.1).
- Do Δ*f* and ΔΓ show unexpected patterns? If plots of ΔΓ versus Δ*f* show circles or spirals, the data may originate from a coupled resonance (Equation (79), Section 6).

### 4.2. The Small-Load Approximation in 1D (Parallel-Plate Model) 

In the following, we go beyond the equation $\omega_{0}\approx {\left(\kappa_{R}/m_{R}\right)}^{1/2}$ and formulate a continuum model. The lumped-element description from Figure 1B is abandoned (no discrete springs, no discrete masses). We treat the resonator as a vibrating body, similar to the bell shown in Figure 1C. Piezoelectric stiffening is ignored, for now. Piezoelectricity at this level simply is a convenience, which allows to probe acoustic resonances by electrical means. 

In the continuum picture, a resonance amounts to a displacement pattern, which occurs time-harmonically and which is easily excited to a large amplitude. The deformation pattern *u*(*x*,*y*,*z*,*t*) is:

(16) $$u(x,y,z)=\hat{u}(x,y,z)\exp(i\omega t)$$

The amplitude $\hat{u}(x,y,z)$ is the mode of vibration. Because the displacement always occurs along *x*, $\hat{u}$ may be viewed as a scalar (rather than a vector). Further simplifying the problem, we let all gradients in the plane vanish. The “parallel plate” can be viewed as an “infinite parallel plate”. It can also be a plate with finite area, $A_{\mathrm{eff}}$, but the edges must not affect the mode of vibration (which is unrealistic for AT-cut quartz because of its anisotropic elasticity). Within the parallel-plate model, the amplitude of displacement, $\hat{u}\equiv {\hat{u}}_{x}$, is a function of *z*, only.

The resonant modes of vibration are solutions of a boundary value problem. The boundary condition here are surfaces, which are free of stress. Because the shear stress is proportional to the shear strain, the shear strain must vanish at the surface (at *z* = 0 and *z* = $d_{q}$):

(17) $$\hat{\sigma }\left(z=0,d_{q}\right)={G_{q}\frac{d\hat{u}}{dz}|}_{z=0,d_{q}}=0$$

The origin of the *z*-axis in Equation (17) is at the back of the plate. For the parallel plate, the modes of vibration are standing waves:

(18) $$u_{n}(z,t)={\hat{u}}_{S}\cos\left(k_{n}z\right)\exp(i\omega t)$$

${\hat{u}}_{S}$ is the displacement amplitude at the surface. 

The boundary condition fixes the wavenumber to discrete values, which are:

(19) $$k_{n}=\frac{n\pi }{d_{q}}\;$$

where *n* is the overtone order. Expressed differently, the wavelength, λ, must be an integer fraction of twice the plate’s thickness. Such discrete sets of solutions are characteristic of boundary value problems. Small deviations of the overtone frequencies from the integer multiples of the fundamental frequency are discussed in Section 8.1.

Critical to the above argument was the fact that the resonator surface was stress-free. The surface must coincide with an antinode. When a sample exerts a periodic stress onto the surface, the resonance condition changes. Within linear acoustics, the stress, ${\hat{\sigma }}_{S}$, is proportional to the displacement, ${\hat{u}}_{S}$. In acoustics, is customary to not discuss the stress-displacement ratio, but rather the stress-velocity ratio, which is the impedance. Velocity, ${\hat{v}}_{S}$, and displacement, ${\hat{u}}_{S}$, are related as ${\hat{v}}_{S}=i\omega {\hat{u}}_{S}$. The stress-velocity ratio at the resonator surface is the load impedance, ${\tilde{Z}}_{L}$. The load impedance is a key variable in the physics of the QCM. The displacement and the stress at $z=d_{q}$ are ${\hat{u}}_{S}\cos\left({\tilde{k}}_{q}d_{q}\right)$ and ${\hat{u}}_{S}G_{q}{\tilde{k}}_{q}\left(-\sin\left({\tilde{k}}_{q}d_{q}\right)\right)\sin\left({\tilde{k}}_{q}d_{q}\right))$, respectively. The stress-velocity ratio follows as:

(20) $$\begin{array}{cc} {\tilde{Z}}_{L} & =\frac{-{\hat{\sigma }}_{S}}{{\hat{v}}_{S}}=\frac{-G_{q}d/{dz(\hat{u}(z))|}_{z=d_{q}}}{{i{\tilde{\omega }}_{\mathrm{res}\;}^{m}\hat{u}(z)|}_{z=d_{q}}}=\frac{{\hat{u}}_{S}G_{q}{\tilde{k}}_{q}\sin\left({\tilde{k}}_{q}d_{q}\right)}{i{\tilde{\omega }}_{\mathrm{res}\;}{\hat{u}}_{S}\cos\left({\tilde{k}}_{q}d_{q}\right)}\\ & =\frac{G_{q}{\tilde{k}}_{q}}{i{\tilde{\omega }}_{\mathrm{res}}}\tan\left({\tilde{k}}_{q}d_{q}\right)=-iZ_{q}\tan\left({\tilde{k}}_{q}d_{q}\right) \end{array}$$

The first minus sign occurs because the stress is exerted by the sample onto the resonator surface (in the direction of −*z*). It follows that:

(21) $${\tilde{Z}}_{L}=-iZ_{q}\tan\left({\tilde{k}}_{q}d_{q}\right)=-iZ_{q}\tan\left(2\pi \left(f_{\mathrm{ref}}+\Delta \tilde{f}\right)\frac{d_{q}}{c_{q}}\right)=-iZ_{q}\tan\left(2\pi \Delta \tilde{f}\frac{d_{q}}{c_{q}}\right)$$

The relations $c_{q}={\left(G_{q}/\rho_{q}\right)}^{1/2}$ and $Z_{q}{=(G}_{q}\rho_{q}{)}^{1/2}$ were used. $Z_{q}$, $c_{q}$, and $f_{\mathrm{ref}}$ are complex, in principle. However, the resonator’s intrinsic losses are not of interest in sensing. Writing them as real parameters certainly affects the absolute value of the bandwidth, but not its shift induced by the sample. The use of an effective complex ${\tilde{k}}_{q}$ (rather than $k_{q}=\omega /c_{q}$) is justified in Appendix D.

The relation $d_{q}{=c}_{q}{/(2f}_{0})$ leads to:

(22) $$-iZ_{q}\tan\left(\pi \frac{\Delta \tilde{f}}{f_{0}}\right)={\tilde{Z}}_{L}$$

Equation (22) is an implicit equation in $\Delta \tilde{f}$, which can be solved numerically. It can also be turned into an explicit equation in $\Delta \tilde{f}=\Delta f+i\Delta \Gamma$ by:

– linearizing the tangent as $\tan\left(\pi \Delta \tilde{f}/f_{0}\right)\approx \pi \Delta \tilde{f}/f_{0}$
– evaluating the load impedance ${\tilde{Z}}_{L}(f)$ at the frequency of the unloaded crystal, rather than the resonance frequency in the presence of the load.

This explicit equation is:

(23) $$\frac{\Delta \tilde{f}}{f_{0}}=\frac{i}{\pi Z_{q}}{\tilde{Z}}_{L}=\frac{i}{\pi Z_{q}}\frac{-{\hat{\sigma }}_{S}}{{\hat{v}}_{S}}$$

Equation (23) is the small-load approximation applied to the parallel plate. Given its importance, it is written down in slightly different form one more time:

(24) $$\frac{\Delta f+i\Delta \Gamma }{nf_{0}}=\frac{\Delta f+i\Delta \Gamma }{f_{\mathrm{ref}\;}}=\frac{\Delta f}{f_{\mathrm{ref}\;}}+i\frac{\Delta D}{2}=\frac{1}{n}\frac{i}{\pi Z_{q}}{\tilde{Z}}_{L}$$

All terms have been normalized to overtone order, as is common in gravimetry. Also, the shift in the dissipation factor, Δ*D*, was used in step 3, replacing 2${\Delta \Gamma /f}_{\mathrm{ref}}$. 

**Figure 10:**
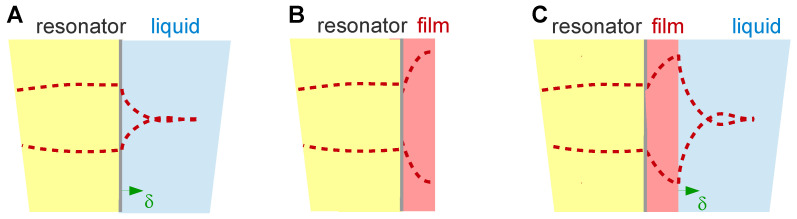
Three simple experimental configurations, which allow for an analytical prediction of Δ*f* + iΔΓ. The dashed lines show the displacement pattern of a shear wave in water (**A**), in a thin film (**B**), and in a sample containing a thin film in water (**C**). The graph is not to scale. The penetration depth of the shear wave in water, δ, is about 200 nm for a 5 MHz crystal. Films with a displacement pattern as shown in (**C**) are a few tens of nanometers thick.

This section deals with stratified layer systems. For those, the stress-velocity ratio follows from how the shear wave bounces back and forth inside the sample. Three simple cases are sketched in Figure 10. However, Equation (23) is more general. Should the sample be structured, laterally, the load impedance may be replaced by its area average:

(25) $$\frac{\Delta \tilde{f}}{f_{0}}=\frac{i}{\pi Z_{q}}{\langle {\tilde{Z}}_{L}\rangle }_{\mathrm{area}\;}=\frac{i}{\pi Z_{q}}{\langle \frac{-{\hat{\sigma }}_{S}}{{\hat{v}}_{S}}\rangle }_{\mathrm{area}\;}$$

Area averaging is possible, because Equation (23) is linear in the load impedance. Within the parallel-plate model, ${\langle \ldots \rangle }_{\mathrm{area}}$ is an unweighted area average. For more realistic resonators, the square of the local amplitude, ${\left|{\hat{u}}_{S}\left(r_{S}\right)\right|}^{2}$, must be included as a weight function (Section 8.1):

(26) $$\frac{\Delta \tilde{f}}{f_{0}}=\frac{i}{\pi Z_{q}}\langle {\tilde{Z}}_{L}\rangle {}_{\mathrm{area},\;\mathrm{weighted}\;}=\frac{i}{\pi Z_{q}}\frac{\iint^{\;}{\tilde{Z}}_{L}\left(r_{S}\right){\left|{\hat{u}}_{S}\left(r_{S}\right)\right|}^{2}d^{2}r_{S}}{\iint^{\;}{\left|{\hat{u}}_{S}\left(r_{S}\right)\right|}^{2}d^{2}r_{S}}$$

$r_{S}$ is a point on the resonator surface. 

The calculation of the stress-velocity ratio is rather simple for thin rigid films and it is also simple for semi-infinite media. For layered systems, there are analytical equations (not all equally simple) [54,55,56]. Some of them are discussed below. For single contacts with small contact area, the stress can be replaced by the ratio of the restoring force, *F*, and the acoustically active area, $A_{\mathrm{eff}}$. Should the sample have a more complicated structure, the stress-velocity ratio needs to be calculated numerically, solving the equations of continuum viscoelasticity for the given geometry (Section 5.3). 

### 4.3. Inertial Loading

**Figure 11:**
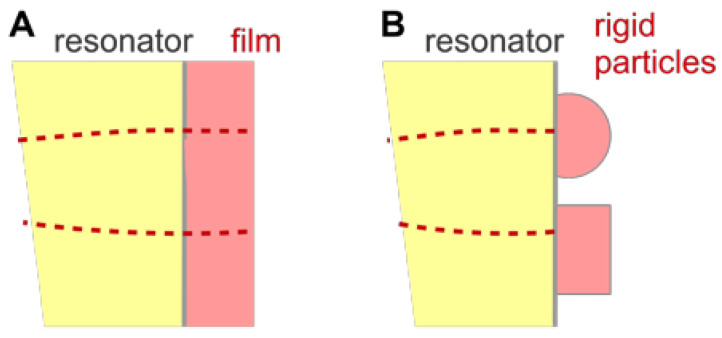
The Sauerbrey equation applies to thin rigid films (**A**) and other rigid samples (**B**). In the latter case, the mass per unit area must be area-averaged.

For a thin rigid film as shown in Figure 11, the stress at the resonator surface is governed by inertia. From Newton’s third law (force = mass × acceleration) it follows that the stress exerted onto the surface is ${i\omega m}_{f}{\hat{v}}_{S}$ with $m_{f}$ the mass per unit area. The load impedance is ${\tilde{Z}}_{L}{=i\omega m}_{f}$. This leads to the Sauerbrey equation:

(27) $$\frac{\Delta \tilde{f}}{f_{0}}\;=\;\frac{i}{{\pi Z}_{q}}{i\omega m}_{f}\;=\;\frac{-{2nf}_{0}}{Z_{q}}m_{f}\;=\;\frac{-{2nf}_{0}}{Z_{q}}{\langle m_{f}\left(r_{S}\right)\rangle }_{\mathrm{area}}\;$$

Angle brackets denote area averaging as before.

### 4.4. Semi-Infinite Viscoelastic Media

For semi-infinite, homogeneous viscoelastic media, the load impedance is equal to the shear-wave impedance, ${\tilde{Z}}_{\mathrm{bulk}}$:

(28) $$\frac{\Delta \tilde{f}}{f_{0}}\;=\;\frac{i}{{\pi Z}_{q}}{\tilde{Z}}_{\mathrm{bulk}}\;$$

The load impedance and the shear-wave impedance must not be confused. ${\tilde{Z}}_{L}$ is the area-averaged ratio of stress and velocity at the resonator surface. The shear-wave impedance, $\tilde{Z}$ or ${\tilde{Z}}_{\mathrm{bulk}}$, is the stress-velocity ratio of a propagating shear wave. $\tilde{Z}$ is a materials constant, given as $\tilde{Z}\;=\;{\left(\rho \tilde{G}\right)}^{1/2}\;=\;\rho {\left(\tilde{G}/\rho \right)}^{1/2}\;=\;\rho \tilde{c}$. The wave impedance governs the reflectivity at interfaces (Equation (37)). 

The relations ${\tilde{Z}}_{\mathrm{bulk}}\;=\;{\left(\rho \tilde{G}\right)}^{1/2}\;=\;\rho \tilde{c}$ and $\tilde{G}\;=\;i\omega \tilde{\eta }$ inserted into Equation (28) lead to:

(29) $$\frac{\Delta f\;+\;i\Delta \Gamma }{f_{0}}\;=\;\frac{i}{{\pi Z}_{q}}\sqrt{i\omega \rho \tilde{\eta }}\;=\;\frac{-1\;+\;i}{\sqrt{2}}\frac{1}{{\pi Z}_{q}}\sqrt{\omega \rho \tilde{\eta }}\;=\;\frac{(-1\;+\;i)}{\sqrt{\pi }Z_{q}}\sqrt{f_{0}}\sqrt{n}\sqrt{\rho \tilde{\eta }}\;\;$$

Equation (29) is the Gordon-Kanazawa relation [23,24]. If $\tilde{\eta }$ is independent of frequency, Δf and ΔΓ scale as $n^{1/2}$. The Gordon-Kanazawa relation can be inverted for viscosity as:

(30) $$\begin{array}{l} {\rho \eta }^{\prime }=\frac{G^{\prime \prime }}{\omega }=-\frac{{\pi Z}_{q}^{2}}{f_{\mathrm{res}}}\frac{1}{2}\frac{\Delta f\Delta \Gamma }{f_{0}^{\;2}}\\ {\rho \eta }^{\prime \prime }=\frac{G\prime }{\omega }=\frac{{\pi Z}_{q}^{2}}{f_{\mathrm{res}}}\frac{\left({\Delta \Gamma }^{2}\;-{\;\Delta f}^{\;2}\right)}{f_{0}^{\;2}} \end{array}$$

In more compact notation, one may write

(31) $$i\omega \rho \tilde{\eta }=\rho \tilde{G}=-{\left({\pi Z}_{q}\frac{\Delta \tilde{f}}{f_{0}}\right)}^{2}\;$$

The density was moved to the left-hand side in order to emphasize that the QCM measures the viscosity-density product (or, equivalently, the product $\rho \tilde{G}$). The density often is known and it often varies less than the shear modulus. For instance, adding a polymer to a solvent much increases the viscosity but leaves the density unchanged within a few percent. Still: ρ and η cannot be determined separately, using the Gordon-Kanazawa relation. (Such a separate determination can be achieved with porous coatings [57].) 

The reference state must be the resonator in air, if the target of the study is a small change in the viscosity (for instance caused by a change in pH). That is so, because $\rho \tilde{G}$ and $\rho \tilde{\eta }$ depend on the square of $\Delta \tilde{f}$. If a change in pH causes a slight change in viscosity, $\delta \tilde{\eta }$, this causes a change in frequency, $\delta \tilde{f}$, following $\delta \left(\rho \tilde{\eta }\right)\;\approx \;-1/\left(i\omega \right)\;{\left({\pi Z}_{q}{/f}_{0}\right)}^{2}\left[2\Delta \tilde{f}\;\delta \tilde{f}\right]$. The term in square brackets is the mixed term of the binomial. A term of the form $\delta {\tilde{f}}^{2}$ was neglected. Δ and δ have different meanings. Δ denotes the difference from the dry state, δ denotes the small shift induced by the change in pH.

For viscoelastic media, ΔΓ is larger than –Δ*f*. For a purely elastic medium (η${}^{\prime }$ = 0, η${}^{\prime \prime }$ > 0, or *G*${}^{\prime }$ > 0, *G*${}^{\prime \prime }$ = 0) the frequency shift vanishes and ΔΓ is equal to ${\left(\rho G^{\prime }\right)}^{1/2}f_{0}/\left({\pi Z}_{q}\right)$. This result may appear as counterintuitive, given that the bandwidth is usually associated with dissipative processes. However, ΔΓ quantifies all forms of energy withdrawn from the resonator (Equation (11)). The energy may or may not be dissipated inside the medium. (Of course, it is dissipated eventually, somewhere.) For the semi-infinite elastic medium, the energy is radiated away towards *z* = +∞ and ΔΓ is nonzero for that reason.

**Figure 12:**
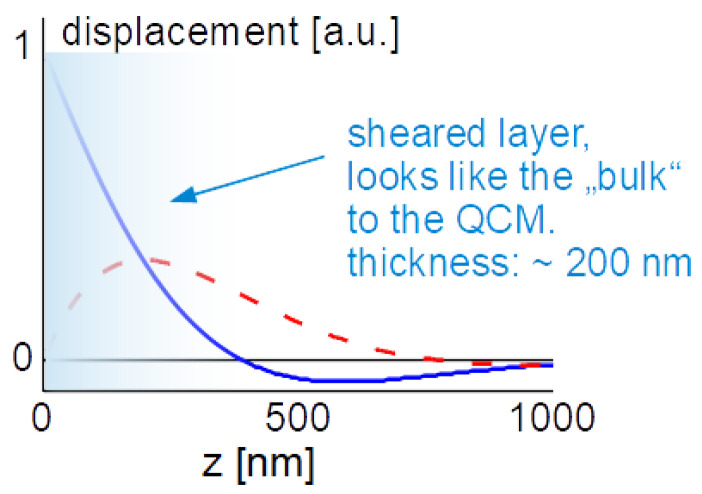
Displacement versus distance for a Newtonian liquid. The blue solid line is the real part, the red dashed line is the imaginary part of the shear wave. The depth of penetration is about 200 nm.

The displacement pattern in a Newtonian liquid is shown in Figure 12. It is of the form:

(32) u^(z,t)=Re(u^Sexp(i(ωt−k˜z)))=u^Scos(ωt−k′z)exp(−k″z)=u^Scos(ωt−zδ)exp(−zδ)

In the last step, a Newtonian liquid was assumed ($\eta^{\prime \prime }$ = 0, $\eta^{\prime }$ independent of frequency). The wave number is then given as $\hat{k}\;=\;(1-i)/\delta$, where δ is the depth of penetration:

(33) $$\delta \;=\;\sqrt{\frac{2\eta }{\rho \omega }}\;$$

With ρ = 10^3^ kg/m^3^, η = 10^−3^ Pa s, and ω = 2π*n* × 5 MHz, the depth of penetration is δ = 252 nm/*n*^1/2^. These values inserted into the Gordon-Kanazawa relation predict −Δ*f* = ΔΓ = 716 Hz/*n*^1/2^. Figure 12 clarifies what “semi-infinite” means for the QCM. The sheared layer seen by the QCM is around 200 nm thick (depending on overtone order and viscosity). 

**Figure 13:**
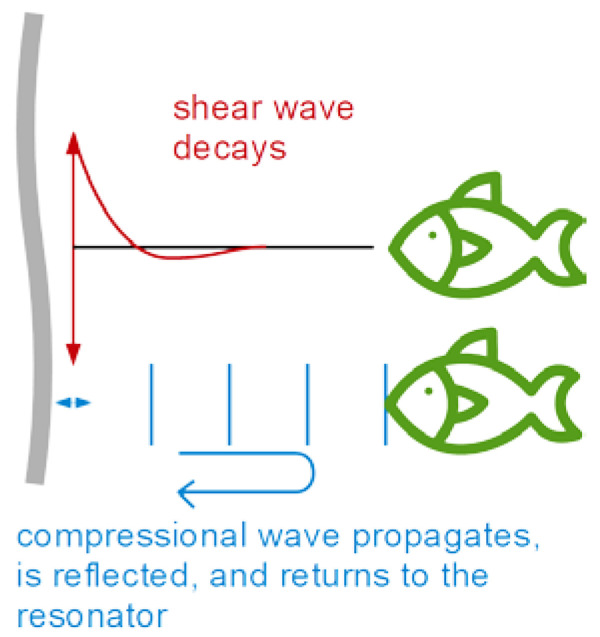
When shear waves dominate the resonator’s response, the QCM response is surface-specific. Flexural admixtures to the mode of vibration and the concomitant compressional waves may spoil surface specificity. Compressional waves may be reflected somewhere in the bulk and return to the crystal.

The finite thickness of the sheared layer turns the liquid-phase QCM into a surface-specific instrument. This is expressed diagrammatically in Figure 13. If exposed to a fluid, the QCM does not see the bulk outside the sheared layer. This is strictly correct to the extent that the QCM indeed vibrates in a pure thickness-shear mode. There are small flexural admixtures to the mode of vibration (Section 8.1). Because of these, Δ*f* and ΔΓ are slightly sensitive to objects outside the sheared layer, whenever these scatter compressional waves.

Can the quartz crystal microbalance be turned into a quartz crystal viscometer? Firstly, there are other simple ways to measure viscosity. Further working against the QCM are artifacts, which are caused by compressional waves (Figure 13) and by the adsorption of debris to the resonator surface, acting as a Sauerbrey load. The Sauerbrey load and the Gordon-Kanazawa load can be separately quantified with the advanced QCMs, but only with these. Problems with mass deposition have a characteristic signature in QCM-based viscometry, which is an apparent negative η${}^{\prime \prime }$. η${}^{\prime \prime }$ is proportional to ${\Delta \Gamma }^{2}\;-{\;\Delta f\;}^{2}$ (Equation (30)). If some adsorbate lowers the frequency following Sauerbrey, this may drive the apparent η${}^{\prime \prime }$ into the negative range when data are analyzed with Equation (30). 

More conceptually, the QCM determines the viscosity at a frequency of a few MHz. For small-molecule liquids, the steady-shear viscosity and oscillatory-shear viscosity at a few MHz are similar. The more interesting fluids, however, often contain soft matter with varying degrees of complexity, which entails relaxation and viscoelastic dispersion. The high-frequency viscosity then may be different from what the engineer cares about. 

Torsional resonators [58,59,60,61] mitigate these problems by virtue of their lower frequency. They are less sensitive to the deposition of mass than the thickness-shear resonators and their frequency (tens of kHz) is closer to the frequencies and time scales of practical relevance. Torsional resonators are commercially available as viscosity sensors [62]. References [63,64,65] report on the use of kHz resonators for an array of other purposes. 

One may envisage a role for the QCM in viscometry, when it comes to small sample volumes. The problem is of much technical relevance and was addressed with other miniaturized sensors, as well [66]. One may deposit small droplets onto the resonator surface. The shifts in frequency and bandwidth are correspondingly small, but they are still well above the noise. One may determine the contact area, *A*_c_, with a camera and attempt to derive the viscosity from the relation [67,68]:

(34) $$\frac{\Delta \tilde{f}}{f_{0}}=\frac{i}{{\pi Z}_{q}}K_{A}\frac{A_{c}}{A_{\mathrm{eff}}}{\tilde{Z}}_{L}\;=\;\frac{i}{{\pi Z}_{q}}K_{A}\frac{A_{c}}{A_{\mathrm{eff}}}\sqrt{i\omega \rho \tilde{\eta }}\;$$

$A_{\mathrm{eff}}$ is the acoustically active area of the plate and $K_{A}$ is a function of the droplet area*,* which takes the amplitude distribution, ${\hat{v}}_{S}{(r}_{S})$, into account (Section 8.1). Typically, this function would be determined by calibration, using liquids with known viscosity. A similar analysis can be applied to a combination of a QCM with a JKR apparatus [68]. The JKR apparatus pushes a lens of a soft material against a substrate and determines the contact radius as a function of the normal force. The JKR apparatus targets the contact-mechanics of soft materials. The substrate may be a QCM, in which case the complex frequency shift reports the material’s high-frequency shear modulus.

From Equation (34), one would expect the prefactors to be the same for frequency and bandwidth. If that was so, the ratio ΔΓ/(−Δ*f*) would be independent of contact area and related to the material’s loss tangent, tan(δ_L_) = $G^{\prime \prime }$/$G^{\prime }$. Experiment shows, however, that the ratio slightly depends on contact area. The problem has to do with the fact that the degree of energy trapping changes when a sample contacts the resonator in the center, only. Energy trapping increases the resonance frequency (see the discussion in in Section 8.1). Its effect on bandwidth is different from its effect on frequency [69]. These problems must be kept in mind when analyzing $\Delta \tilde{f}$ with Equation (34). 

**Figure 14:**
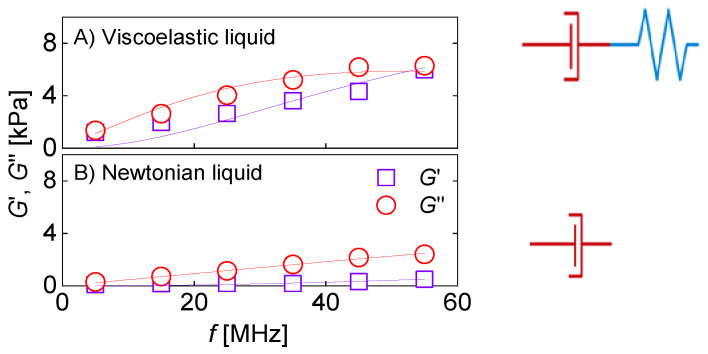
The complex shear modulus of two concentrated antibody solutions as determined with a QCM and Equation (31). The lines are fits with a Maxwell model ($G^{\prime }+{iG}^{\prime \prime }={G_{\infty }}^{\prime }/\left(1-\omega \tau \right)$). The top and the bottom show data obtained on a viscoelastic liquid (**A**) and on a Newtonian liquid (**B**), respectively. Adapted from [70].

QCM-based viscometry amounts to high-frequency rheology on bulk samples. When applied to engine oils, the QCM’s high frequency is a disadvantage. For other complex fluids, the high-frequency viscoelasticity actually is of interest because it depends on the fluid’s internal organization. Pharmaceuticals for parenteral administration are often formulated as concentrated protein solutions, which display viscoelastic relaxation in the MHz range [70]. Protein-protein interactions (PPIs) may turn these solutions into weak gels. High-frequency rheology is among the techniques probing such interactions [70,71,72]. Figure 14 shows an example, taken form a study on pharmaceutical formulations. For the data in Figure 14A, the shear modulus as derived with Equation (31) displays viscoelasticity with a characteristic dependence on frequency. The data could be fitted with the Maxwell model (a lumped-element model consisting of a spring in series with a dashpot). The relaxation time was in the range of a few tens of nanoseconds. The data shown at the bottom did not show this kind of viscoelasticity. This formulation looked like a Newtonian liquid to the QCM. 

Note that time-temperature-superposition (TTS) is not needed to interpret these experiments. For certain types of polymers, one may study the “high-frequency” viscoelasticity with conventional, low-frequencies rheometers by cooling the sample, such that the relaxations of interest slow down and then are accessible to the instrument. Complex liquids often are not thermorheologically simple in this sense. For these, acoustic instrumentation operating at high frequencies (such as the QCM, but not limited to the QCM [73]) is needed.

### 4.5. Films in Air

**Figure 15:**
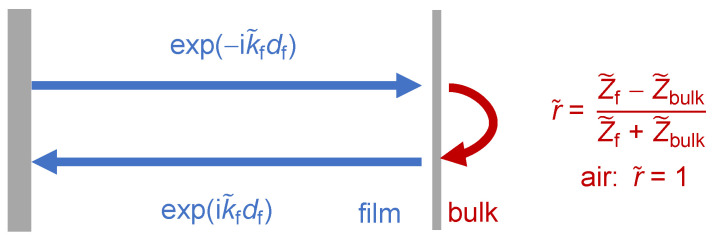
The stress at the surface of a resonator coated with a film contains a contribution from the reflected wave.

If the sample contains interfaces with some impedance contrast, the reflected wave contributes to the periodic stress at the resonator surface in proportion to $-{\tilde{Z}}_{f}{\hat{v}}_{\leftarrow }$, where ${\hat{v}}_{\leftarrow }$ is the amplitude of the reflected wave, evaluated at the resonator surface (Figure 15). ${\tilde{Z}}_{f}$ is the film’s wave impedance. There is a minus sign because the reflected wave travels towards –*z*. Dividing by the total velocity, ${\hat{v}}_{\to }\;+\;{\hat{v}}_{\leftarrow }$, the load impedance is found to be:

(35) $$\;{\tilde{Z}}_{L}={\tilde{Z}}_{f}\frac{{\hat{v}}_{\to }-{\hat{v}}_{\leftarrow }}{{\hat{v}}_{\to }+{\hat{v}}_{\leftarrow }}\frac{{\hat{v}}_{\to }-{\hat{v}}_{\leftarrow }}{{\hat{v}}_{\to }+{\hat{v}}_{\leftarrow }}={\tilde{Z}}_{f}\frac{1-\frac{{\hat{v}}_{\leftarrow }}{{\hat{v}}_{\to }}}{1+\frac{{\hat{v}}_{\leftarrow }}{{\hat{v}}_{\to }}}\frac{1-\frac{{\hat{v}}_{\leftarrow }}{{\hat{v}}_{\to }}}{1+\frac{{\hat{v}}_{\leftarrow }}{{\hat{v}}_{\to }}}={\tilde{Z}}_{f}\frac{1-{\tilde{r}}_{S}}{1+{\tilde{r}}_{S}}\frac{1-{\tilde{r}}_{S}}{1+{\tilde{r}}_{S}}$$

The ratio ${\tilde{r}}_{S}\;=\;{\hat{v}}_{\leftarrow }/{\hat{v}}_{\to }$ is the complex reflectivity evaluated at the resonator surface. The QCM may be viewed as an acoustic reflectometer (and may compete with other reflectometers, for instance described in [74,75]). In particular, there is a close correspondence between the physics of the QCM and optical reflectometry (Section 9.2). The reflectivity of the sample can be inferred from Equation (35), solved for ${\tilde{r}}_{S}$:

(36) $${\tilde{r}}_{S}\;=\;\frac{1\;-\;\tilde{\alpha }}{1\;+\;\tilde{\alpha }},\;\;\;\;\;\;\tilde{\alpha }\;=\;\frac{{\pi Z}_{q}}{i{\tilde{Z}}_{f}}\frac{\left(\Delta f\;+\;i\Delta \Gamma \right)}{f_{0}}\;$$

Equation (23) was used when expressing $\tilde{\alpha }$ as a function of Δ*f* + iΔΓ.

The calculation of ${\tilde{r}}_{S}$ for a film in air is sketched in Figure 15. ${\tilde{r}}_{S}$ is given as $\exp\left(-2i{\tilde{k}}_{f}d_{f}\right)\;\times \;\tilde{r}$ where the exponential covers the propagation through the film (twice, hence the factor of 2) and $\tilde{r}$ is the reflection amplitude at the film-air interface. The reflectivity of a wave at an interface between two media with different wave impedances, ${\tilde{Z}}_{1}$ and ${\tilde{Z}}_{2}$, is:

(37) $${\tilde{r}}_{12}\;=\;\frac{\;{\hat{v}}_{\leftarrow }}{{\hat{v}}_{\to }}\;=\;\frac{{\tilde{Z}}_{1}\;-\;{\tilde{Z}}_{2}}{{\tilde{Z}}_{1}\;+\;{\tilde{Z}}_{2}}\;$$

The proof exploits that the velocity and the stress are continuous at the interface. Equivalently, the reflectivity can be calculated from the conservation of energy and momentum. It is worthwhile to remind oneself of two related situations:

– When an optical wave hits an interface at normal incidence, the reflectivity is $\left(n_{r,1}-n_{r,2}\right)/\left(n_{r,1}{+n}_{r,2}\right)$. While one might think so, the refractive index, *n_r_*, is not strictly the same as the impedance of the optical wave, but it is related to this impedance.
– Upon a central elastic collision of two spheres, the velocity of the first sphere after collision is ${\hat{v}}_{\leftarrow }$ = ${\hat{v}}_{\to }\left(m_{1}-m_{2}\right)/\left(m_{1}{+m}_{2}\right)$. The mass takes the role, which the impedance has for waves.

Because ${\tilde{Z}}_{\mathrm{air}}$ = 0, the reflectivity at the film-air interface is unity. From Equations (35) and (37), the load impedance follows as:

(38) $${\tilde{Z}}_{L}\;=\;{\tilde{Z}}_{f}\frac{1\;-\exp\left(-2i{\tilde{k}}_{f}d_{f}\right)}{1\;+\;\exp\left(-2i{\tilde{k}}_{f}d_{f}\right)}\;$$

Euler’s relation implies:

(39) $$\begin{array}{cc} {\tilde{Z}}_{L} & ={\tilde{Z}}_{f}\frac{\exp\left(+i{\tilde{k}}_{f}d_{f}\right)\;-\;\exp\left(-i{\tilde{k}}_{f}d_{f}\right)}{\exp\left(+i{\tilde{k}}_{f}d_{f}\right)\;+\;\exp\left(-i{\tilde{k}}_{f}d_{f}\right)}\\ & ={\tilde{Z}}_{f}\frac{2\mathrm{isin}\left({\tilde{k}}_{f}d_{f}\right)}{2\cos\left({\tilde{k}}_{f}d_{f}\right)}=i{\tilde{Z}}_{f}\tan\left({\tilde{k}}_{f}d_{f}\right) \end{array}$$

This result inserted into Equation (22) yields:

(40) $${\mathrm{iZ}}_{q}\tan\left(\pi \frac{\Delta f\;+\;i\Delta \Gamma }{f_{0}}\right)\;=\;i{\tilde{Z}}_{f}\tan\left({\tilde{k}}_{f}d_{f}\right)\;$$

Equation (40) was first derived by Lu and Lewis [76]. The Lu-Lewis equation does not invoke the small-load approximation. It is an implicit equation in Δ*f* + iΔΓ, which must be solved numerically. An analysis of frequency shifts based on the Lu-Lewis equation is implemented in some commercial film-thickness monitors. The algorithm is called “Z-match method” [77,78]. Film-thickness monitors often become heavily loaded when crystals are not replaced between deposition runs. 

A side remark on film thickness monitors: The frequency shift in these instruments can be above 1 MHz. Resonators with plane-convex surfaces are used, which tolerate large loading but only work reliably on the fundamental. This is one of the cases, where the small-load approximation does not apply. 

In order to apply the Z-match algorithm to these data (Δ*f* determined on a single overtone), the wave impedance of the layer must be known. Some values for metals are tabulated in [79]. One might also use a numerical solution of the Lu-Lewis equation (Appendix C.2) as part of a fitting process, determining not only the thickness, but also the layer’s viscoelastic parameters [80]. This analysis of course requires experimental values of frequency and bandwidth on a few overtones as input. Otherwise, the problem is underdetermined.

The Lu-Lewis equation does not make use of the small-load approximation. If the small-load approximation is employed (which amounts to inserting Equation (39) into Equation (23)), the following result is found:

(41) $$\frac{\Delta f\;+\;i\Delta \Gamma }{f_{0}}=\frac{i}{{\pi Z}_{q}}i{\tilde{Z}}_{f}\tan\left({\tilde{k}}_{f}d_{f}\right)\;$$

**Figure 16:**
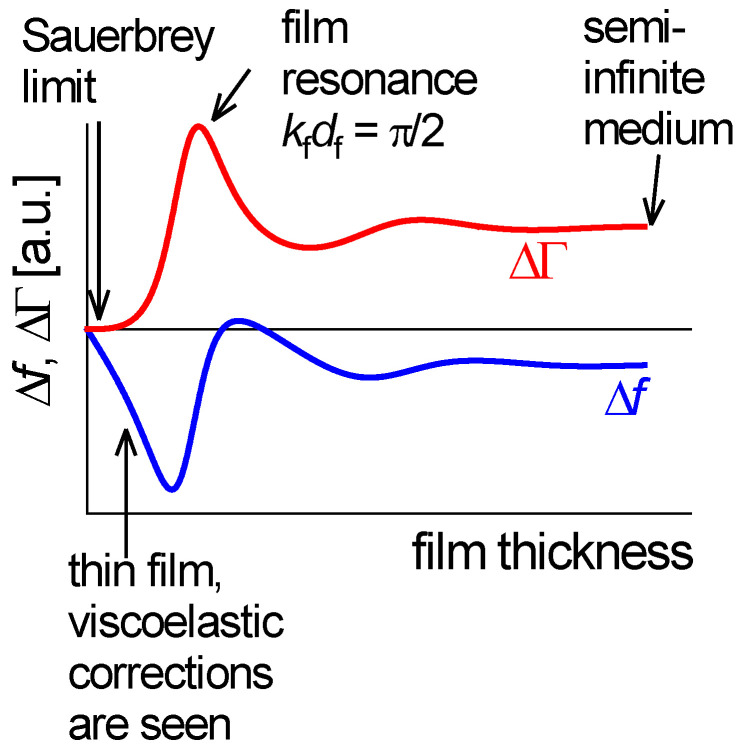
Δ*f* and ΔΓ as a function of thickness, following Equation (41). The medium is assumed to be lossy, hence the broad film resonance. The loss tangent used in the calculation was tan(δ_L_) = *G*’’/*G*’ = 0.84.

Figure 16 shows Δ*f* and ΔΓ as predicted by Equation (41). The following sections address the four different regimes indicated with arrows in Figure 16.

#### 4.5.1. Very Thin Films (Sauerbrey Limit) 

At very low thickness, a Taylor expansion of the tangent in Equation (41) as tan(*x*) ≈ *x* leads to:

(42) $$\frac{\Delta f+i\Delta \Gamma }{f_{0}}=\frac{i}{\pi Z_{q}}i{\tilde{Z}}_{f}\tan\left({\tilde{k}}_{f}d_{f}\right)\approx \frac{i}{\pi Z_{q}}i\rho_{f}{\tilde{c}}_{f}\frac{\omega }{{\tilde{c}}_{f}}d_{f}=-\frac{\omega m_{f}}{\pi Z_{q}}$$

This is the Sauerbrey result. The relations ${\tilde{Z}}_{f}{\;=\;\rho }_{f}{\tilde{c}}_{f}$ and ${\tilde{k}}_{f}=\omega /{\tilde{c}}_{f}$ were used. −Δ*f*/*n* is proportional to the film’s mass per unit area. ΔΓ vanishes because the film does not undergo shear deformation to any appreciable extent under its own inertia. Again, the Sauerbrey result is more general than Equation (42) because area averaging may be applied. 

#### 4.5.2. Infinite Thickness

In the limit of infinite thickness, ${\tilde{Z}}_{f}$ turns into ${\tilde{Z}}_{\mathrm{bulk}}$. The tangent turns into −*i* as long as ${k_{f}}^{\prime \prime }$ > 0:

(43) $$\frac{\Delta f\;+\;i\Delta \Gamma }{f_{0}}=\frac{i}{{\pi Z}_{q}}i{\tilde{Z}}_{\mathrm{bulk}}\underset{df\to \infty }{\lim}\tan\left(\left({k_{f}}^{\prime }-{ik_{f}}^{\prime \prime }\right)d_{f}\right)=\frac{i}{{\pi Z}_{q}}{\tilde{Z}}_{\mathrm{bulk}}\;$$

The Gordon-Kanazawa relation is recovered.

#### 4.5.3. Thin Viscoelastic Films

If the film is thin, but still thick enough to let viscoelasticity be noticeable, the tangent can be expanded to 3rd order as $\tan({\tilde{k}}_{f}d_{f})\;\approx \;{\tilde{k}}_{f}d_{f}\;+\;{\left({\tilde{k}}_{f}d_{f}\right)}^{3}/3$. This regime is of much practical importance. The Taylor expansion leads to:

(44) $$\begin{array}{cc} \frac{\Delta f\;+\;i\Delta \Gamma }{f_{0}} & \approx \;\frac{-1}{{\pi Z}_{q}}{\tilde{Z}}_{f}\left({\tilde{k}}_{f}d_{f}\;+\;\frac{1}{3}{\left({\tilde{k}}_{f}d_{f}\right)}^{3}\right)\\ & =\;\frac{-1}{{\pi Z}_{q}}{\omega m}_{f}\left[1\;+\;\frac{{\left(n\pi \right)}^{2}}{3}\frac{Z_{q}^{2}}{{\tilde{Z}}_{f}^{2}}{\left(\frac{m_{f}}{m_{q}}\right)}^{2}\right]\\ & =\;\frac{-1}{{\pi Z}_{q}}{\omega m}_{f}\left[1\;+\;\frac{{\left(n\pi \right)}^{2}}{3}\frac{{\tilde{J}}_{f}}{\rho_{f}}Z_{q}^{2}{\left(\frac{m_{f}}{m_{q}}\right)}^{2}\right] \end{array}$$

The relations ${\tilde{k}}_{f}=\omega /{\tilde{c}}_{f}=\omega {\left(\rho_{f}{\tilde{J}}_{f}\right)}^{1/2}$, ω = 2π*n*$f_{0}$ = π*n*$Z_{q}$/$m_{q}$, and ${\tilde{Z}}_{f}{\;=\;(\rho }_{f}/{\tilde{J}}_{f}{)}^{1/2}$ were used. $\tilde{J}=1/\tilde{G}$ is the shear compliance. 

It is instructive to express $\Delta \tilde{f}/n$ as a function of $n^{2}$:

(45) $$\frac{\Delta \tilde{f}}{n}\;\approx \;\frac{{2f}_{0}^{\;2}}{Z_{q}}m_{f}\left[{1\;+\;n}^{2}{\tilde{J}}_{f}\left(\frac{\pi^{2}Z_{q}^{2}}{{3\rho }_{f}}\right){\left(\frac{m_{f}}{m_{q}}\right)}^{2}\right]\;\;$$

**Figure 17:**
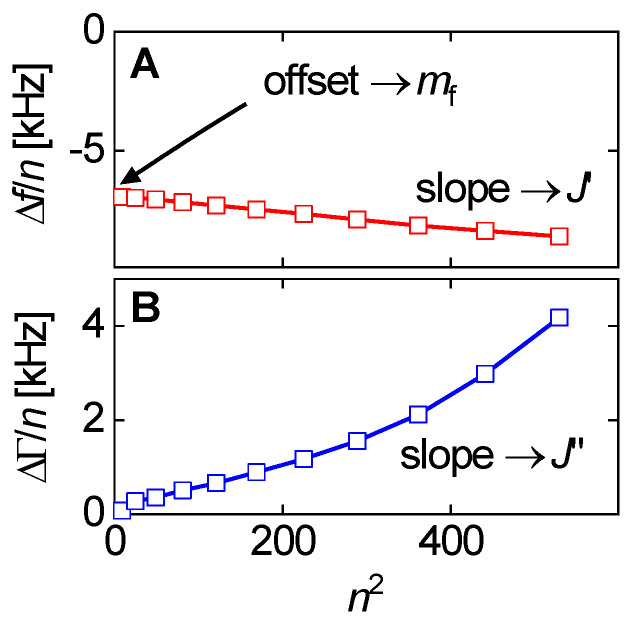
Plots of Δ*f*/*n* and ΔΓ/*n* versus $n^{2}$ as motivated by Equation (46). The film thickness as derived from the offset in panel (**A**) is about 1.6 µm. The slope d(Δ*f*/*n*)/d($n^{2}$) is almost constant, indicating that ${J_{f}}^{\prime }$ only weakly depends on frequency. There *is* such a dependence on frequency in (**B**). The data were taken on a spin-cast film of polyisobutylene. Adapted from [81].

Clearly, both −Δ*f*/*n* and ΔΓ/*n* depend on $n^{2}$ [81]. If (!) ${J_{f}}^{\prime }$ and ${J_{f}}^{\prime \prime }$ themselves do not depend on frequency, Equation (45) describes a linear relation between −Δ*f*/*n* and ΔΓ/*n*, on the one hand, and $n^{2}$, on the other. The slopes then are proportional to the elastic compliance, ${J_{f}}^{\prime }$, and the viscous compliance, ${J_{f}}^{\prime \prime }$ (Figure 17). However, ${J_{f}}^{\prime }$ and ${J_{f}}^{\prime \prime }$ may depend on frequency, in which case the lines in Figure 17 have some curvature.

When the film is not much softer than the crystal, a correction to Equation (45) is needed because the assumptions inherent to the small-load approximation produce a sizeable error. In the derivation of Equation (23), the term tan($\pi \Delta \tilde{f}{/f}_{0}$) was linearized, while the term $\tan\left({\tilde{k}}_{f}d_{f}\right)$ from Equation (41) was expanded to 3rd order. This is an inconsistency, which can be removed with a systematic perturbation calculation [82]. Box 2 addresses the issue in more detail.

Box 2 **3rd-order perturbation applied to the films on a parallel plate.**

On the way to Equation (44)), a tangent contained in the load impedance was Taylor-expanded to 3rd order, while a similar tangent contained in the Lu-Lewis equation (Equation (40))) was linearized. This is an inconsistency, which can be removed [82]. At the same time, one needs to deal with the fact that the load should be evaluated at the resonance frequency of the loaded crystal, rather than the reference frequency. Dealing with these complications, one should also take electrode effects into account, that is, treat the 2-layer system (electrode plus film).

For the 2-layer system, the Lu-Lewis equation turns into

$$-iZ_{q}\tan\left(\pi \frac{\Delta f}{f_{0}}\right)=i\frac{{\tilde{Z}}_{e}\tan\left({\tilde{k}}_{e}d_{e}\right)+{\tilde{Z}}_{f}\tan\left({\tilde{k}}_{f}d_{f}\right)}{1-{\tilde{Z}}_{f}/{\tilde{Z}}_{e}\tan\left({\tilde{k}}_{f}d_{f}\right)\tan\left({\tilde{k}}_{e}d_{e}\right)}$$

The indices *e* and *f* denote the electrode and the film, respectively. The equations become more compact if the following variables are used:

$$\mu_{f}=\frac{m_{f}}{m_{q}},\;\mu_{e}=\frac{m_{e}}{m_{q}},\;{\tilde{\zeta }}_{f}(\omega )=\frac{{\tilde{J}}_{f}(\omega )}{\rho_{f}}Z_{q}^{2}-1,\;{\tilde{\zeta }}_{e}(\omega )=\frac{{\tilde{J}}_{e}(\omega )}{\rho_{e}}Z_{q}^{2}-1$$

3rd-order perturbation leads do

$$\frac{\Delta \tilde{f}}{nf_{0}}\approx -\left(1-2\mu_{e}+3\left(1+\frac{{\left(n\pi \right)}^{2}}{3}{\tilde{\zeta }}_{e}\right)\mu_{e}^{2}\right)\mu_{f}+\left(1-3\left(1+\frac{{\left(n\pi \right)}^{2}}{3}{\tilde{\zeta }}_{e}\right)\mu_{e}\right)\mu_{f}^{2}-\left(1+\frac{{\left(n\pi \right)}^{2}}{3}{\tilde{\zeta }}_{f}\right)\mu_{f}^{3}$$

Importantly, the coefficient to *n*^2^ contains the thickness and the wave impedance of the *electrode*. If the electrode is neglected, the equation simplifies as

$$\frac{\Delta \tilde{f}}{nf_{0}}\approx -\mu_{f}+\mu_{f}^{2}-\left(1+\frac{{\left(n\pi \right)}^{2}}{3}{\tilde{\zeta }}_{f}\right)\mu_{f}^{3}$$

The terms independent of *n* slightly modify the Sauerbrey equation. They are negligible in practice. The *n*-dependent term leads to Equation (46)).

In view of these complications, one might also go back to the Lu-Lewis equation, solve it numerically, and use this solution when fitting a model to experimental data. The Python code in Appendix C.2 solves the Lu-Lewis equation.

The above remarks mostly concern films in air. For films in liquids, Equation (49)) can be trusted. At least, it is not grossly invalidated in quantitative terms by the full numerical solution to the Lu-Lewis equation.

Piezoelectric stiffening is not covered by this formalism. Piezoelectric stiffening does affect the result, in principle, but the changes are small [80].

3rd-order perturbation leads to:

(46) $$\frac{\Delta f+i\Delta \Gamma }{f_{0}}\approx \frac{-\omega m_{f}}{\pi Z_{q}}\left[1+\frac{{\left(n\pi \right)}^{2}}{3}\left(\frac{{\tilde{J}}_{f}}{\rho_{f}}Z_{q}^{2}-1\right){\left(\frac{m_{f}}{m_{q}}\right)}^{2}\right]$$

The difference between Equations (45) and (46) is essential for stiff films (with small $\tilde{J}$). Analyzing data from thin, glassy polymer films with Equation (45) can easily produce a negative apparent shear modulus. The analysis becomes even more complicated if the viscoelastic properties of the electrode are taken into account. For more details see [80]. 

Equation (46) is the basis of quantitative rheometry on thin films [83,84]. The QCM as an instrument is unique in this regard because it does not require a clamp on the other side of the film. Thin layers can be clamped from both sides [85], in principle, but these experiments (using the surface forces apparatus, SFA) are more demanding than the QCM. 

Films in air shear under their own inertia, hence the proportionality to $m_{f}^{2}$ in the viscoelastic correction. A film thickness of a few tens of nanometers is needed to see visco-elasticity. Thinner films would have to be extremely soft to show such effects. If −Δ*f*/*n* does not agree between overtones for such films, this may go back to an overtone-dependent modal mass (Section 8.6). An increase in bandwidth has been seen in experiments on monolayers of noble gases [86]. This increase in damping is not easily explained in the standard framework of molecular interactions. Superlubricity may be an explanation [87].

#### 4.5.4. The Film Resonance

At $d_{f}$ ≈ λ/4, Equation (41) hits the “film resonance” [88,89,90]. The film resonance is an example of a coupled resonance (Section 6) and is therefore labeled with subscript *CR*. In the thickness range of the film resonance, Δ*f* increases with thickness and ΔΓ goes through a maximum. The dependence of Δ*f* + iΔΓ on $d_{f}$ looks like a resonance (cf. Figure 3A) and we briefly convince ourselves that the algebra confirms that. If the imaginary part of ${\tilde{k}}_{f}$ is small, the real part of the tangent at the pole first goes to +∞ and later returns from −∞. Close to the pole, one may write tan(*x*) = 1/cot(*x*) = 1/cot(*y* + π/2). The variable *x* was substituted by *y* = *x* − π/2. Taylor expansion of the cotangent to 1st order in *y* leads to cot(*y* + π/2) = −*y*. The tangent turns into −1/(*x* − π/2) and Equation (41) is approximated by

(47) $$\begin{array}{ll} \frac{\Delta \tilde{f}}{f_{0}} & =\frac{-1}{\pi Z_{q}}{\tilde{Z}}_{f}\tan\left({\tilde{k}}_{f}d_{f}\right)\approx \frac{-{\tilde{Z}}_{f}}{\pi Z_{q}}\frac{1}{\frac{\pi }{2}-{\tilde{k}}_{f}d_{f}}=\frac{-{\tilde{Z}}_{f}}{\pi Z_{q}}\frac{1}{\frac{\pi }{2}-\omega \frac{d_{f}}{{\tilde{c}}_{f}}}=\frac{-{\tilde{Z}}_{f}}{\pi Z_{q}}\frac{\frac{{\tilde{c}}_{f}}{d_{f}}}{\frac{{\tilde{c}}_{f}\pi }{2d_{f}}-\omega }\\ & =\frac{-{\tilde{Z}}_{f}}{\pi Z_{q}}\frac{2}{\pi }\frac{{\tilde{\omega }}_{\mathrm{CR}}}{{\tilde{\omega }}_{\mathrm{CR}}-\omega } \end{array}$$

**Figure 18:**
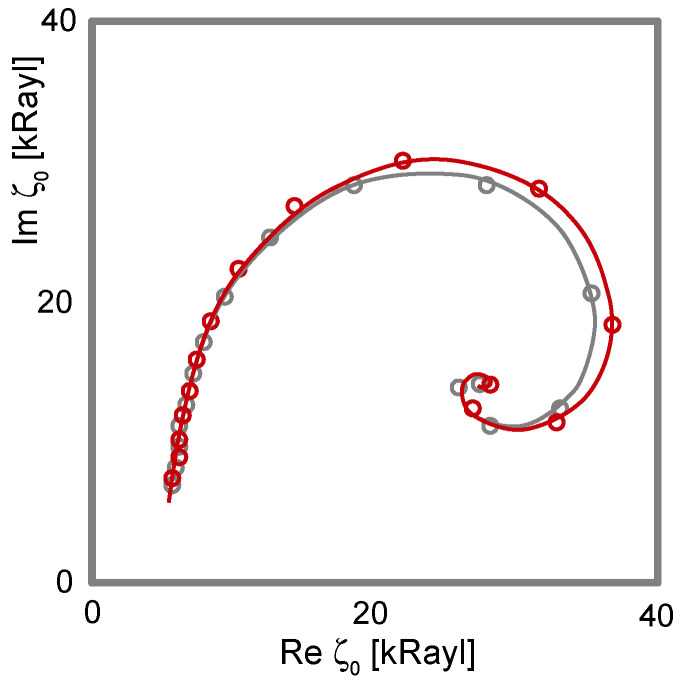
An experimental example of a film resonance. The sample consisted of a polyelectrolyte multilayer, the thickness of which was gradually increased by repeated dipping. When displayed in polar form, the data form a spiral. These authors display the load impedance rather than the complex frequency shift. The load impedance here has the unit “Rayl”, in the honor of John William Strutt, 3rd Baron Rayleigh. 1 MKS-Rayl is equal to 1 kg/(m^2^s). Red and black data denote results obtained with an even number of layers and with an odd number of layers, respectively. The difference occurs because anionic and cationic layers alternate. Adapted from [91].

The resonance frequency of the film, ${\omega^{\prime }}_{\mathrm{CR}}$, is governed by the condition ${k_{f}}^{\prime }d_{f}=\pi /2$. An experimental example of a film resonance is shown in Figure 18. For the study of soft films, the film resonance mostly is a problem. The data can rarely be fitted well by Equation (41). 

**Figure 19:**
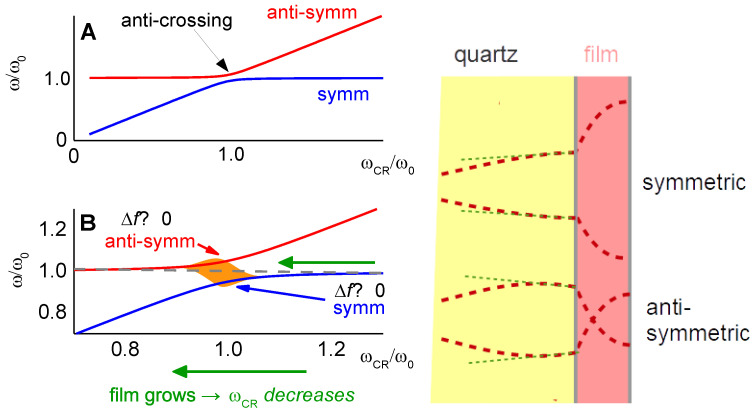
If the small-load approximation is abandoned, a film resonance produces two separate peaks in the conductance trace, $G_{\mathrm{el}}\left(\omega \right)$, corresponding to two separate modes of vibration. The shear gradients inside the film have opposite sign for the two modes, hence the labels “symmetric” and “antisymmetric”. Far away from the coupling condition, $\omega_{0}$ and $\omega_{\mathrm{CR}}$ are not affected by coupling (to the right and to the left in panel (**A**)). If the two frequencies match and if the two modes indeed couple, anticrossing results. For a more quantitative treatment see Chapter 4.63 in [5]. Panel (**B**) shows an enlargement of panel (**A**) in the region of anti-crossing. If the bandwidth is large, the two modes are not actually resolved (sketched in orange in panel (**B**)). When the film becomes thicker, $\omega_{\mathrm{CR}}$ approaches the coupling condition from above (green arrow). Δ*f* is less than zero, following Sauerbrey. Because the two modes are not resolved at the coupling condition, the peak in $G_{\mathrm{el}}\left(\omega \right)$ is broad. The center of the peak gradually moves up, because the antisymmetric mode becomes stronger. Eventually, the peak sharpens again and returns to the original frequency from above. A similar behavior is seen in Figure 16, based on Equation (41).

The film resonance is among the examples, where the small-load approximation is not quite good enough. The problem occurs if the film is not lossy, that is, if ${k_{f}}^{\prime \prime }\ll {k_{f}}^{\prime }$. In the range of the film resonance, the Lu-Lewis equation then has two solutions, corresponding to a “symmetric” and an “antisymmetric” mode (Figure 19). For ${k_{f}}^{\prime }d_{f}\;<\;\pi /2$, the mode with negative Δ*f* has the larger amplitude. When ${k_{f}}^{\prime }d_{f}\approx \pi /2$, the mode with positive Δ*f* grows in amplitude and eventually takes over. This picture emerges in the frame of parallel-plate model (not using the small-load approximation). More specifically, it emerges, when the calculation of the electrical admittance is done with the Mason equivalent circuit, briefly mentioned at the end of Section 8.4. For the details, we refer the reader to the literature [54,92]. 

Modes growing and shrinking in magnitude are seen in experiment when swelling polymer films with $d_{f}\approx \lambda /4$ in solvent vapor. However, an antisymmetric mode as shown in Figure 19 is not easily identified. Most often, one of the anharmonic sidebands grows and eventually becomes the largest peak in the conductance trace. A full understanding of the film resonance would require a realistic model of the resonator in 3D. That is difficult, in the first place. Also, the patterns of the anharmonic sidebands (Section 8.2) show some variability between crystals and batches. Crystal imperfections play a role.

**Figure 20:**
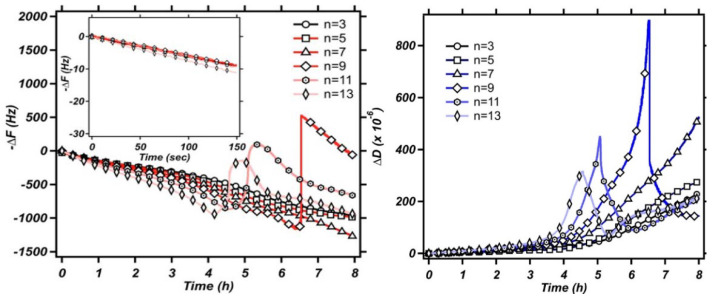
An example of a film resonance. At some point, the frequency jumps, as discussed in the caption to Figure 19. Reprinted with permission from [96]. Copyright 2017 American Chemical Society. These experiments occurred in liquid. Film resonances can occur in liquids, as well, because Equation (49) also contains terms of the form $\tan\left({\tilde{k}}_{f}d_{f}\right)$.

If the details of the film resonance are so difficult, why even bother? Firstly, the film resonance is an instructive example of a coupled resonance. The coupled resonance will concern us further in Section 6. Also, the film resonance is occasionally seen in experiment. Figure 20 shows an example. These authors were interested in the dissolution of polymer films and in the preservation of old paintings. The QCM worked well, basically, but the frequency at some point jumped. Knowing about the film resonance, one understands the jump.

### 4.6. Layers Adsorbed from a Liquid Phase

#### 4.6.1. General

Many adsorbates from the liquid phase do not have a sharp interface with the bulk. Still, the viscoelastic box profile (that is, the homogeneous film with thickness $d_{f}$) is a good starting point. Using the reflectivity at an interface from Equation (37), the frequency shift is [93,94,95]:

(48) $$\frac{\Delta f+i\Delta \Gamma }{f_{0}}=\frac{i}{\pi Z_{q}}{\tilde{Z}}_{f}\frac{1-\exp\left(-2i{\tilde{k}}_{f}d_{f}\right)\frac{{\tilde{Z}}_{f}-{\tilde{Z}}_{\mathrm{bulk}\;}}{{\tilde{Z}}_{f}+{\tilde{Z}}_{\mathrm{bulk}\;}}}{1+\exp\left(-2i{\tilde{k}}_{f}d_{f}\right)\frac{{\tilde{Z}}_{f}-{\tilde{Z}}_{\mathrm{bulk}\;}}{{\tilde{Z}}_{f}+{\tilde{Z}}_{\mathrm{bulk}\;}}}$$

Applying Euler’s relation to the right-hand side (similar to Equation (39)) yields

(49) $$\frac{\Delta f+i\Delta \Gamma }{f_{0}}=\frac{-{\tilde{Z}}_{f}}{\pi Z_{q}}\cdot \frac{{\tilde{Z}}_{f}\tan\left({\tilde{k}}_{f}d_{f}\right)-i{\tilde{Z}}_{\mathrm{bulk}\;}}{{\tilde{Z}}_{f}+i{\tilde{Z}}_{\mathrm{bulk}\;}\tan\left({\tilde{k}}_{f}d_{f}\right)}$$

While not immediately evident, Equation (49) is equivalent to the Voigt-model from [56] and, also, to [54,91].

Equation (49) also leads to a film resonance, but the resonance condition is different from $k_{f}\prime d_{f}=\pi /2$ (which is the resonance condition for dry films). The film resonance can be seen while films grow from a liquid phase or while they dissolve into a liquid phase [96]. It is also observed when surface-attached gels [97] or polymer brushes [89] swell and deswell. In the latter case, however, the layer becomes softer as it swells. ${\tilde{k}}_{f}$ and ${\tilde{Z}}_{f}$ vary strongly. 

For thick gels swelling in a solvent, $\Delta \tilde{f}$ as a function of the swelling degree can also be qualitatively portrayed as a transition from Sauerbrey-type behavior to Gordon-Kanazawa-type behavior. The compact layer obeys the Sauerbrey relation, possibly with a small viscoelastic correction. –Δ*f*/*n* becomes larger as the layer swells. At some point, the layer thickness is comparable to the depth of penetration of the shear wave. Beyond this point, the layer appears as a soft semi-infinite medium. Δ*f* and ΔΓ then report the medium’s complex shear modulus, regardless of its thickness. The shear wave no longer reaches to the top of the film. An example for this behavior is shown in [97].

Cell cultures behave like soft gel layers in this regard. The shear wave usually does not reach to the top. It probes the layer’s shear stiffness at the bottom rather than the layer thickness. Cell cultures have been extensively studied with the QCM [98,99,100]. The interpretation is usually based on certain correlations between Δ*f* and ΔΓ, on the one hand, and the conditions of the experiment, on the other. Quantitative modeling is difficult. −Δ*f*/*n* may certainly be converted to an apparent mass, but the emphasis here is on “apparent”.

#### 4.6.2. Thin Adsorbates

We now turn to thin viscoelastic layers. When the tangent in Equation (49) is expanded to 1st order in $d_{f}$, one arrives at:

(50) $$\frac{\Delta f+i\Delta \Gamma }{f_{0}}\approx \frac{i}{\pi Z_{q}}\left({\tilde{Z}}_{\mathrm{bulk}\;}+i{\tilde{Z}}_{f}{\tilde{k}}_{f}d_{f}\left[1-\frac{{\tilde{Z}}_{\mathrm{bulk}\;}^{2}}{{\tilde{Z}}_{f}^{2}}\right]\right)$$

It is convenient to choose the resonator immersed in the liquid as the reference state, which results in:

(51) $$\begin{array}{cc} \frac{\Delta f+i\Delta \Gamma }{f_{0}} & =\frac{i}{\pi Z_{q}}i{\tilde{Z}}_{f}{\tilde{k}}_{f}d_{f}\left[1-\frac{{\tilde{Z}}_{\mathrm{bulk}}^{2}}{{\tilde{Z}}_{f}^{2}}\right]\\ & =\frac{-\omega m_{f}}{\pi Z_{q}}[1-\frac{{\tilde{J}}_{f}(\omega )}{\rho_{f}}i\omega \left(\rho_{\mathrm{bulk}}\eta_{\mathrm{bulk}}\right]\\ & =\frac{-\omega m_{f}}{\pi Z_{q}}\left[1-2\pi in\frac{{\tilde{J}}_{f}(\omega )}{\rho_{f}}f_{0}\rho_{\mathrm{bulk}}\eta_{\mathrm{bulk}}\right] \end{array}$$

The relation ${\tilde{k}}_{f}{\tilde{Z}}_{f}$${\;=\;\omega \rho }_{f}\;$ was used in line 2. Equation (51) can be rearranged as:

(52) $$\frac{\Delta f}{n}\;+\;i\frac{\Delta \Gamma }{n}\;\approx \;\frac{{-2f}_{0}^{2}}{Z_{q}}m_{f}\left[1-n\;\left({J_{f}}^{\prime }\left(\omega \right)-i{J_{f}}^{\prime \prime }\left(\omega \right)\right)\left({2\pi i\;f}_{0}\frac{\rho_{\mathrm{bulk}}}{\rho_{f}}\eta_{\mathrm{bulk}}\right)\right]\;$$

If the film is much stiffer than the liquid (if $\left|{\tilde{Z}}_{f}\left|\;\gg \;\right|{\tilde{Z}}_{\mathrm{bulk}}\right|$), this relation reduces to the Sauerbrey equation. For rigid layers and even moderately rigid layers, the Sauerbrey contribution and the Gordon-Kanazawa contribution to the complex frequency shift simply are additive [101,102]. This analysis approach is applied to electrogravimetry [103]. Electrodeposition and electroetching can be analyzed with the Sauerbrey equation. (Roughness may take an effect, though [104].)

The second term in the square brackets in Equation (52) is a viscoelastic correction. This term differs characteristically from the viscoelastic correction in Equation (46) (experiments in air). The difference goes back to the fact that a film immersed in a liquid feels a stress from the other side. It is partially clamped by the liquid. In air, films are sheared by their own inertia, only. For films in air, viscoelastic effects are seen after expanding the tangent to 3rd order as ${\tilde{k}}_{f}d_{f}\;+\;{\left({\tilde{k}}_{f}d_{f}\right)}^{3}/3$. In liquids, viscoelastic effects enter the picture in 1st-order Taylor expansion, already. Even molecularly thin films are sheared by the adjacent liquid and can be studied with regard to their softness. 

**Figure 21:**
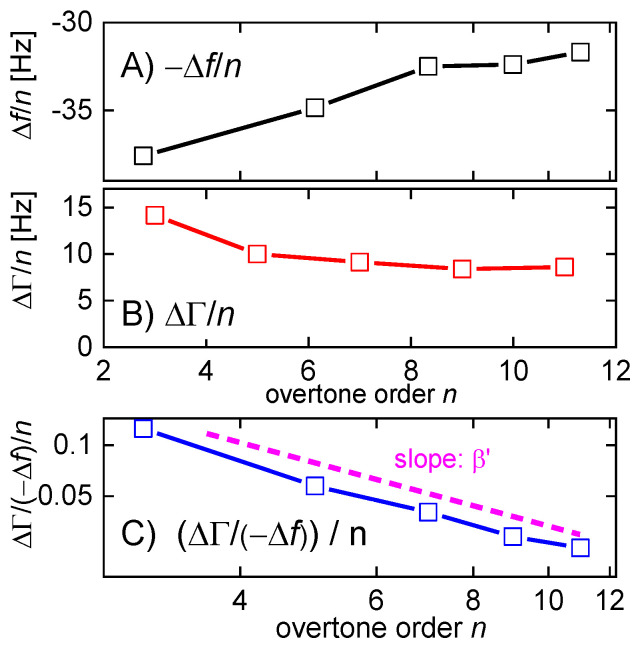
Soft adsorbates from a liquid phase produce sloped lines in plots of Δ*f*/*n* in panel (**A**) and ΔΓ/*n* in panel (**B**) versus *n*. As opposed to the case of the soft film in air (Figure 17), the apparent mass as derived with the Sauerbrey equation is smaller than the true mass. Panel (**C**) shows ΔΓ/(−Δ*f*) normalized to overtone order. ΔΓ/(−Δ*f*)/*n* is proportional to ${J_{f}}^{\prime }$ (Equation (54)). The slope in this log-log plot is the power law exponent $\beta^{\prime }$ (Section 4.7). The sample is a block copolymer adsorbed to the gold surface, where the soluble part forms a brush-like structure. Data kindly shared by Anna M.C. Maan, University of Groningen, FSE-Zernike Institute for Advanced Materials.

For soft films in liquids, the apparent mass as derived with the Sauerbrey equation is smaller than the film’s mass [105]. Voinova et al. call this the “missing-mass effect” [106]. Viscoelastic effects can be recognized by plotting Δ*f*/*n* and ΔΓ/*n* versus *n* (Figure 21). A positive slope indicates a finite ${J_{f}}^{\prime \prime }$. A nonzero ΔΓ/*n* indicates finite ${J_{f}}^{\prime }$.

Why does the slope have positive sign? (It has negative sign in air, Figure 17.) The missing-mass effect is caused by the film being clamped from the other side. The stress exerted by the liquid is proportional to ωη, meaning, increases with overtone order.

Ideally, one would wish to derive ${J_{f}}^{\prime }$ and ${J_{f}}^{\prime \prime }$ (or equivalently, the moduli, ${G_{f}}^{\prime }$ and ${G_{f}}^{\prime \prime }$) on each overtone. This would amount to a rheological spectrum, albeit in a limited frequency range. Unfortunately, the problem is underdetermined as long as the film thickness is not known. The elastic compliance alone, however, *can* be determined on each overtone, at least approximately. In the thin-film limit, $m_{f}$ can be eliminated from Equation (52) by taking the ratio of ΔΓ and –Δ*f*:

(53) $$\frac{\Delta \Gamma }{-\Delta f}\;\approx \;\frac{{J_{f}}^{\prime }{\omega \eta }_{\mathrm{bulk}}}{1\;-\;{J_{f}}^{\prime \prime }{\omega \eta }_{\mathrm{bulk}}}\;$$

It was assumed that $\rho_{\mathrm{bulk}}$ ≈ $\rho_{\mathrm{film}}$. For such thin layers, the ratio ΔΓ/(Δf) is independent of layer thickness. It is a materials parameter. Equation (53) further simplifies if the denominator is about unity. That is often an acceptable approximation because polymer films (even when swollen) are much stiffer than the ambient liquid. (They are stiffer at MHz frequencies than at low frequencies.) The denominator in Equation (53) can be rewritten as $1-{J_{f}}^{\prime \prime }/{J_{\mathrm{bulk}}}^{\prime \prime }$ where ${J_{\mathrm{bulk}}}^{\prime \prime }=\;{\left({\omega \eta }_{\mathrm{bulk}}\right)}^{-1}$ is the liquid’s viscous compliance. If ${J_{f}}^{\prime \prime }\ll {J_{\mathrm{bulk}}}^{\prime \prime }$, the denominator is unity, leading to [107]:

(54) $$\frac{\Delta \Gamma }{\Delta f}\;\approx {J_{f}}^{\prime }{\omega \eta }_{\mathrm{bulk}}\;=\;{J_{f}}^{\prime }{2\pi nf}_{0}\eta_{\mathrm{bulk}}\;$$

${J_{f}}^{\prime }$ can be rather robustly inferred from QCM data. This will be important for the discussion of viscoelastic dispersion at the end of Section 4.7.

In linear rheology, viscoelasticity is commonly expressed in terms of the shear modulus $\tilde{G}=G^{\prime }\;+\;iG^{\prime \prime }$. For the QCM, it is more convenient to instead use the shear compliance, $\tilde{J}=1/\tilde{G}=J^{\prime }-iJ^{\prime \prime }$, because the compliance occurs in the numerator in Equations (51) and (46). The trivial case (Sauerbrey-like behavior) corresponds to zero compliance. The conversion between $\tilde{G}$ and $\tilde{J}$ is nontrivial because they are complex:

(55) $$J^{\prime }=\frac{G^{\prime }}{{G^{\prime }}^{2}+{G^{\prime \prime }}^{2}},\;\;\;\;J^{\prime \prime }=\frac{G^{\prime \prime }}{{G^{\prime }}^{2}+{G^{\prime \prime }}^{2}}\;G^{\prime }=\frac{J^{\prime }}{{J^{\prime }}^{2}+{J^{\prime \prime }}^{2}},\;\;\;\;G^{\prime \prime }=\frac{J^{\prime \prime }}{{J^{\prime }}^{2}+{J^{\prime \prime }}^{2}}\;$$

Writing *J*${}^{\prime }$ ≈ 1/*G*${}^{\prime }$ often is grossly inaccurate. The conversion is easier for the absolute values and the loss tangent:

(56) $$\left|J\right|=\frac{1}{\left|G\right|}\;,\;\;\;\;\;\;\;\;\;\;\tan\left(\delta_{L}\right)=G^{\prime \prime }/G^{\prime }=J^{\prime \prime }/J^{\prime }\;$$

The loss tangent is the same for the modulus and the compliance. Another relation worth remembering is $\tilde{\eta }=\tilde{G}/(i\omega )$ with $\tilde{\eta }=\eta^{\prime }-i\eta^{\prime \prime }=G^{\prime \prime }/\omega -iG^{\prime }/\omega$ the viscosity. 

Because the film thickness enters Equation (51) as a linear term, shifts in frequency and bandwidth resulting from multiple films are additive (assuming $k_{i}^{\prime }d_{i}\ll 1$ for all layers):

(57) $$\frac{\Delta f\;+\;i\Delta \Gamma }{f_{0}}\;\approx \;\frac{\omega }{{\pi Z}_{q}}\underset{i}{\sum }m_{i}\left[1-\frac{{\tilde{J}}_{i}}{\rho_{i}}{i\omega \rho }_{\mathrm{bulk}}\eta_{\mathrm{bulk}}\right]\;\;$$

Equation (57) holds in an integral sense:

(58) $$\begin{array}{cc} \frac{\Delta f\;+\;i\Delta \Gamma }{f_{0}} & \approx \;\frac{-\omega }{{\pi Z}_{q}}\int_{0}^{\infty }\left[1-\frac{\tilde{J}\left(z\right)}{\rho \left(z\right)}{i\omega \rho }_{\mathrm{bulk}}\eta_{\mathrm{bulk}}\right]\rho \left(z\right)\;dz\\ & =\frac{-\omega }{{\pi Z}_{q}}\int_{0}^{\infty }\left[\frac{{\tilde{Z}}^{2}\left(z\right)-{\tilde{Z}}_{\mathrm{bulk}}^{2}}{{\tilde{Z}}^{2}\left(z\right)}\right]\rho \left(z\right)\;dz\\ & \approx \frac{-{\omega \rho }_{\mathrm{bulk}}}{{\pi Z}_{q}}\int_{0}^{\infty }\left[\frac{\tilde{G}\left(z\right)-{\tilde{G}}_{\mathrm{bulk}}}{\tilde{G}\left(z\right)}\right]\;dz\\ & \approx \frac{-{\omega \rho }_{\mathrm{bulk}}}{{\pi Z}_{q}}\int_{0}^{\infty }\left[\frac{\tilde{\eta }\left(z\right)-\eta_{\mathrm{bulk}}}{\tilde{\eta }\left(z\right)}\right]\;dz\; \end{array}$$

ρ(*z*) ≈ $\rho_{\mathrm{bulk}}$ was assumed in lines 3 and 4. Equation (58) may also be expressed in terms of density and viscosity:

(59) $$\frac{\Delta f\;+\;i\Delta \Gamma }{f_{0}}\;\approx \;\frac{\omega }{{\pi Z}_{q}}\rho_{\mathrm{bulk}}\int_{0}^{\infty }\left[\frac{\rho \left(z\right)}{\rho_{\mathrm{bulk}}}-\frac{\eta_{\mathrm{bulk}}}{\tilde{\eta }\left(z\right)}\right]\;dz\;\;$$

**Figure 22:**
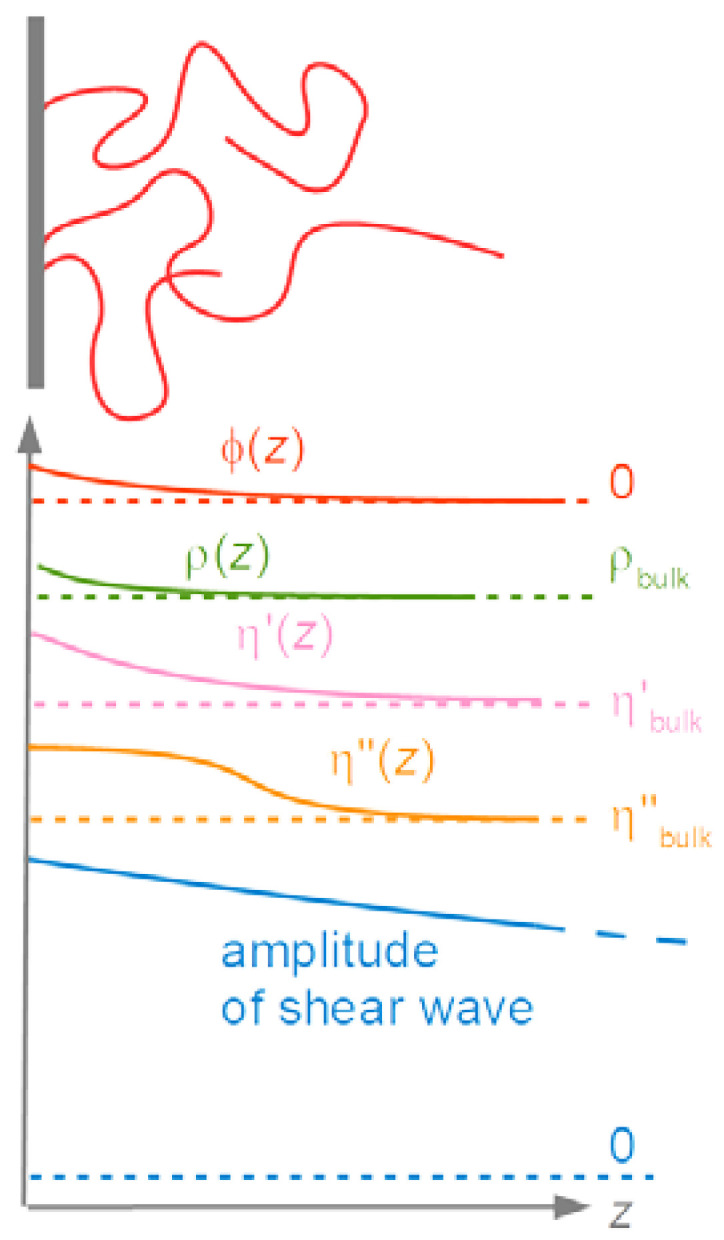
The frequency response induced by thin adsorbates is proportional to an integral of the response function from Equation (59). The *z*-range, in which ρ(*z*) and η(*z*) are significantly different from the corresponding bulk values, must be much below the depth of penetration of the shear wave, δ, in order to let the integral formulation be applicable. The displacement profile is sketched at the bottom. For most polymers, ρ(*z*) is similar to ρ_bulk_. The dependence of the viscosity, $\tilde{\eta }\left(z\right),$ on the polymer volume fraction, ϕ(*z*), is strong and not easily guessed based on simple models.

The term in square brackets is a contrast function. The integral can be viewed as a “shear-wave acoustic moment” of a profile of the polymer volume fraction, ϕ(*z*), as displayed in Figure 22.

Separating real and imaginary parts in Equation (59) leads to:

(60) $$\frac{\Delta f}{f_{0}}\;\approx \;\frac{{\omega \rho }_{\mathrm{bulk}}}{{\pi Z}_{q}}\int_{0}^{\infty }\left(\frac{\rho \left(z\right)}{\rho_{\mathrm{bulk}}}\;-J^{\prime \prime }\left(z\right){\omega \eta }_{\mathrm{bulk}}\right)\;dz\;\frac{\Delta \Gamma }{f_{0}}\;\approx \;\frac{{\omega \rho }_{\mathrm{bulk}}}{{\pi Z}_{q}}\int_{0}^{\infty }\left(J^{\prime }\left(z\right){\omega \eta }_{\mathrm{bulk}}\right)\;dz$$

One can apply the Sauerbrey equation to adsorbates and derive an apparent mass, ${\tilde{m}}_{\mathrm{app}}\left(n\right)$, which is:

(61) $${\tilde{m}}_{\mathrm{app}}\left(n\right)=\frac{Z_{q}}{{2nf}_{0}^{2}}\Delta \tilde{f}\left(n\right)\;\approx \;\int_{0}^{\infty }\rho_{\mathrm{bulk}}\left[\frac{\rho \left(z\right)}{\rho_{\mathrm{bulk}}}-\frac{\eta_{\mathrm{bulk}}}{\tilde{\eta }\left(n,z\right)}\right]dz\;$$

In principle, the parameter ${\tilde{m}}_{\mathrm{app}}$ is a complex function of *n*. Application of the Sauerbrey equation is most meaningful, if the imaginary part of ${\tilde{m}}_{\mathrm{app}}$ is small and if ${\tilde{m}}_{\mathrm{app}}$ weakly depends on *n*. 

For sufficiently rigid layers, the apparent acoustic thickness, $m_{\mathrm{app}}$/ρ, is close to the geometric thickness. It is slightly smaller because of the missing mass effect (Equation (52)). There is, however, another situation which also lets ${\tilde{m}}_{\mathrm{app}}$ be real and independent of *n*. If there is a near-surface layer of a purely Newtonian liquid with increased viscosity (possibly also with an increased density), the contrast function in Equation (59) is again real and independent of *n*. In this case, the apparent acoustic thickness may be much smaller than the range with increased viscosity because the contrast function is less than unity. This situation is encountered in electrochemistry. The diffuse double layer is viscoelastic, in principle, but sometimes modeling it as a layer with increased Newtonian viscosity (no elasticity) matches the experimental data well [48]. This frequency shift should not be misinterpreted as the consequence of adsorption and desorption.

A side remark: If the near-surface viscosity is lower than the bulk viscosity, Equation (59) predicts a negative apparent Sauerbrey thickness. This situation is further discussed in Section 4.8.

Similar to the apparent mass, an apparent elastic shear compliance can be derived following Equation (54) as:

(62) $${J_{\mathrm{app}}}^{\prime }(n)=\frac{\Delta \Gamma }{-\Delta f}\frac{1}{{\omega \eta }_{\mathrm{bulk}}}\;\approx \;\frac{\int_{0}^{\infty }J^{\prime }\left(z\right)\rho \left(z\right)\;dz}{\int_{0}^{\infty }\left({1-\omega \eta }_{\mathrm{bulk}}J^{\prime \prime }\left(z\right)\right)\rho \;\left(z\right)dz}\;\approx \;\frac{\rho_{\mathrm{bulk}}}{{m^{\prime }}_{\mathrm{app}}}\int_{0}^{\infty }J^{\prime }\left(z\right)dz\;$$

${J_{\mathrm{app}}}^{\prime }$ may well depend on *n*. If it does, this may have to do with viscoelastic dispersion (Figure 21C, Section 4.7).

It is instructive to remind oneself of the differences between Equation (52) (thin film in a liquid) and Equation (46) (thin film in air):

- For the thin film in air, the Sauerbrey mass is larger than the true mass. It is smaller for the thin film in a liquid (because of the missing-mass effect).
- The viscoelastic correction scales as *n*^2^ in air, while it scales as *n* in liquids (constant compliance assumed).
- In both cases, ${J_{f}}^{\prime }$ and ${J_{f}}^{\prime \prime }$ are the coefficients to the viscoelastic correction. In air, ${J_{f}}^{\prime }$ enters the correction for −Δ*f*/*n*, while ${J_{f}}^{\prime \prime }$ enters the correction for ΔΓ/*n*. The roles of ${J_{f}}^{\prime }$ and ${J_{f}}^{\prime \prime }$ are reversed in liquids.
- In air, the viscoelastic correction scales as the square of the film’s mass because the film shears under its own inertia. Viscoelastic effects are only seen for films with a thickness of at least a few tens of nanometers. The film in a liquid is clamped from the other side. Viscoelastic effects are seen even for layers with a thickness corresponding to a few molecules.

In air, the 3rd-order perturbation analysis (Box 2) makes significant correction to the small-load approximation. In liquids, this difference exists, in principle, but it is negligible in practice.

#### 4.6.3. Thick Layers

We now turn to adsorbates, which are comparable in thickness to the depth of penetration, δ. One might apply the multilayer formalism and depict the profiles, $\tilde{G}\left(z\right)$ and ρ(z), as a sequence of many thin films with the wave being reflected at the numerous interfaces. It is easier to solve the wave equation directly. If the functions *G*’(*z*), *G*’’(*z*), and ρ(*z*) are given, one may calculate the displacement profile, $\hat{u}\left(z\right)$, with one of the software packages, which numerically solve ordinary differential equations (Scipy being among them). One infers the complex frequency shift from $\hat{u}\left(z=0\right)$ and $d\hat{u}/dz(z=0)$ as:

(63) $$\frac{\Delta f\;+\;i\Gamma }{f_{0}}=\frac{i}{{\pi Z}_{q}}{\tilde{Z}}_{L}=\frac{i}{{\pi Z}_{q}}\frac{-{\hat{\sigma }}_{S}}{{\hat{v}}_{S}}=\frac{i}{{\pi Z}_{q}}\frac{-\rho \tilde{G}{\left(z\;=\;0\right)\frac{d\hat{u}\left(z\right)}{dz}|}_{z\;=\;0}}{i\omega \hat{u}\left(z\;=\;0\right)}$$

The function $\hat{u}\left(z\right)$ is obtained by solving the wave equation, which is

(64) $$-\rho \left(z\right)\omega^{2}\hat{u}\left(z\right)=\frac{d\hat{\sigma }}{dz}=\frac{d}{dz}\left(\tilde{G}\left(z\right)\frac{d\hat{u}\left(z\right)}{dz}\right)=\tilde{G}\left(z\right)\frac{d^{2}\hat{u}\left(z\right)}{{dz}^{2}}+\frac{d\tilde{G}\left(z\right)}{dz}\frac{d\hat{u}\left(z\right)}{dz}\;$$

**Figure 23:**
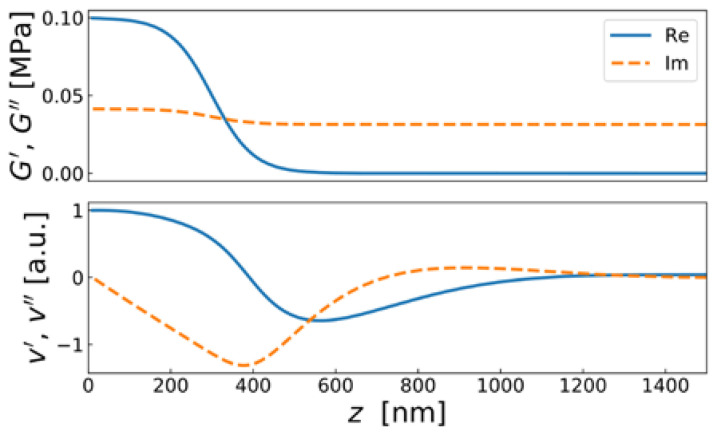
Top: The shear modulus as assumed in the Python code in Appendix C.1. Bottom: Normalized velocity profile resulting from a numerical solution of the wave equation (Equation (64)) with $\tilde{G}\left(z\right)$ as shown at the top. The density was assumed as constant. Δf and ΔΓ are computed from the slope of the velocity profile at *z* = 0.

Note that the shear modulus appears inside the outer derivative because $\tilde{G}\left(z\right)$ itself is a function of *z*. Appendix C.1 contains Python code solving this problem. The bottom of Figure 23 shows a velocity profile, $\hat{v}\left(z\right)$, obtained with this code. The top shows the shear modulus, *G*’(*z*) + i*G*’’(*z*), which entered this calculation. 

### 4.7. Viscoelastic Dispersion and High-Frequency Rheology

Given that the QCM can determine shear stiffness, one wonders whether it can also do viscoelastic spectroscopy. The term “viscoelastic spectroscopy” here is synonymous to “high-frequency rheology”. To cut a longer argument short, the QCM cannot explicitly do viscoelastic spectroscopy on thin films, even if one makes peace with the power laws from Equation (65) or Equation (66). It can, in principle, but the error bars are large.

The viscoelastic constants depend on frequency if the sample undergoes relaxations with rates comparable to the frequencies of excitation. ($\tilde{G}$ and $\tilde{J}$ then are complex for the same reason.) The QCM can yield spectroscopic information, but only to a limited extent. Firstly, the frequency range is only about one decade wide. Also, it is impossible to determine $J^{\prime }$ and $J^{\prime \prime }$ on every overtone separately, because this problem is underdetermined. As long as the thickness is not *a priori* known, 2*n* + 1 parameters would have to be derived from Δ*f* and ΔΓ on *n* overtones. 

**Figure 24:**
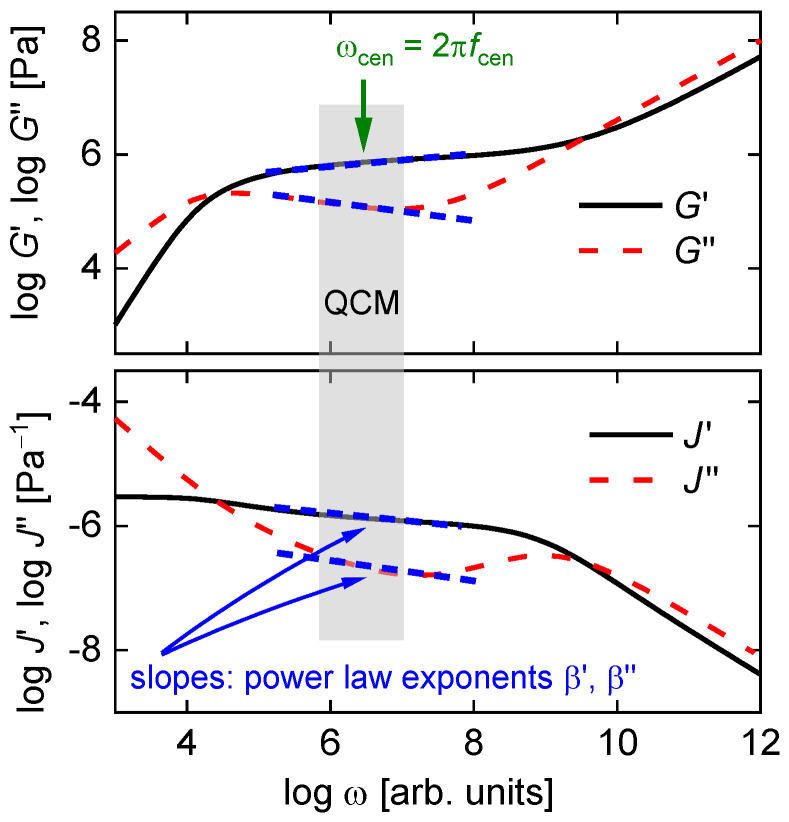
The shear modulus of viscoelastic media depends on frequency. The plot shows a typical rheological spectrum of a solution of a long-chain linear polymer. The frequency scale extends over many decades, while the QCM only covers about one decade. In this limited frequency range, $G^{\prime }(\omega )$ and $G^{\prime \prime }(\omega$) can be approximated by power laws (dashed blue lines). The chosen example displays what is called the rubber plateau. Would the frequency scale extend further to the right, there would be a second maximum in $G^{\prime \prime }(\omega$), caused by segmental relaxations and the glass transition.

At this point, one can exploit the narrow frequency range and the fact that rheological spectra usually are smooth. Rheological spectra are displayed on a logarithmic scale (Figure 24). Over a single decade, the frequency dependence of ${G_{f}}^{\prime }$ and ${G_{f}}^{\prime \prime }$ can be approximated by power laws with power law exponents γ’ and γ’’:

(65) $${G_{f}}^{\prime }\left(f\right)\;\approx {{\;G}_{f}}^{\prime }\left(f_{\mathrm{cen}}\right){\left(\frac{f}{f_{\mathrm{cen}}}\right)}^{\gamma^{\prime }}\;{G_{f}}^{\prime \prime }\left(f\right)\;\approx {{\;G}_{f}}^{\prime \prime }\left(f_{\mathrm{cen}}\right){\left(\frac{f}{f_{\mathrm{cen}}}\right)}^{\gamma^{\prime \prime }}\;$$

The subscript “cen” refers to a frequency in the center of the range accessible to the QCM. Typical is $f_{\mathrm{cen}}$ = 30 MHz. ${J_{f}}^{\prime }\left(\omega \right)$ and ${J_{f}}^{\prime \prime }\left(\omega \right)$ are approximated as:

(66) $${J_{f}}^{\prime }\left(f\right)\;\approx {{\;J}_{f}}^{\prime }\left(f_{\mathrm{cen}}\right){\left(\frac{f}{f_{\mathrm{cen}}}\right)}^{\beta^{\prime }}\;{J_{f}}^{\prime \prime }\left(f\right)\;\approx {{\;J}_{f}}^{\prime \prime }\left(f_{\mathrm{cen}}\right){\left(\frac{f}{f_{\mathrm{cen}}}\right)}^{\beta^{\prime \prime }}\;$$

The power law exponents in Equation (65) and Equation (66) differ. Worse, power laws in ${G_{f}}^{\prime }$ and ${G_{f}}^{\prime \prime }$ do not turn into power laws in ${J_{f}}^{\prime }$ and ${J_{f}}^{\prime \prime }$ after transformation from $\tilde{G}$ to $\tilde{J}$ with Equation (55). A set of power laws in ${G_{f}}^{\prime }$ and ${G_{f}}^{\prime \prime }$ is not strictly equivalent to the corresponding set in ${J_{f}}^{\prime }$ and ${J_{f}}^{\prime \prime }$.

Following from the Kramers-Kronig relations, β’, β’’, γ${}^{\prime }$, and γ${}^{\prime \prime }$ must be in certain ranges. If viscoelasticity is expressed in terms of compliance (${J_{f}}^{\prime }$ and ${J_{f}}^{\prime \prime }$ as in Equation (66)), one has −2 < β${}^{\prime }$ < 0 and −1 < β${}^{\prime \prime }$ < 1. If moduli are used (${G_{f}}^{\prime }$ and ${G_{f}}^{\prime \prime }$), one has 0 < γ${}^{\prime }$ < 2 and −1 < γ${}^{\prime \prime }$ < 1. The software packages supplied by Biolin use the variables “µ” and “η”. µ is equal to ${G_{f}}^{\prime }$ and η is equal to ${G_{f}}^{\prime \prime }$/ω. The power law exponent for η is between −2 and 0.

In the context of the QCM, power laws applied to the compliance, $\tilde{J}$, are closer to experiment because one of the corresponding power law exponents (only one, β${}^{\prime }$) can be determined from experiment with good accuracy. It is often difficult to obtain a robust fit with all five fit parameters free (thickness, $\left|J_{f}\right|$, $\tan\left(\delta_{L}\right)$, β${}^{\prime }$, β${}^{\prime \prime }$). Robust fits are obtained, though, if β${}^{\prime \prime }$ is fixed. β${}^{\prime }$ alone often is fitted with fair accuracy, because the film’s mass, $m_{f}$, enters the imaginary part of Equation (52) as a prefactor, only. (It enters the real part of Equation (52) as a prefactor *and* as an additive term.)

Rather than fixing β${}^{\prime \prime }$, one might also fix the difference between β${}^{\prime }$ and β${}^{\prime \prime }$. For polymers, the Rouse model and the Zimm model both predict β${}^{\prime }$ ≈ β${}^{\prime \prime }$ in the high-frequency regime, see the right-hand side in Figure 24.

A side remark: For experiments in air, the situation is reversed. β${}^{\prime \prime }$ rather than β${}^{\prime }$ can be determined with good confidence from the curvature in Figure 17B. One may read a curvature from Figure 17A, as well, but the accuracy suffers from the unknown offset (proportional to the mass).

Determination of both β${}^{\prime }$ and β${}^{\prime \prime }$ would be attractive because thin films are not easily studied with conventional rheology. Demanding equipment is needed [85] and others. Again: the unknown layer thickness is the problem. If the layer thickness can be determined independently, this will help. The power law exponents give access to spectroscopic information. One of them (β${}^{\prime }$) can be derived from the fits. With a model at hand (Zimm, Rouse, reptation, …) the value of β${}^{\prime }$ can be interpreted. 

### 4.8. Slip

“Slip” here denotes slip of a simple liquid at a solid wall. Slip in that sense is the exception rather than the rule. Often, the “no-slip condition” is a suitable boundary condition for liquid flows. In the following, slip does not denote sliding between solid surfaces and, also, does not denote wall slip of complex liquids, induced by shear thinning under large stress [108]. Slip in simple liquids is associated with a near-surface layer of reduced viscosity. The above statements are phrased in terms of the continuum picture. Arguably, a molecular description would be more appropriate. That would make no difference for the experiment. 

**Figure 25:**
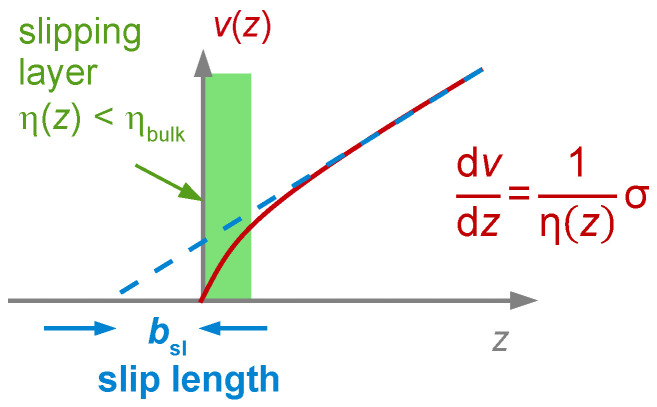
A decreased viscosity close to an interface moves the extrapolated plane of zero shear to negative *z*. The distance between the plane of zero shear and the surface is the slip length, $b_{s1}$.

Given constant shear stress, the shear gradient in the low-viscosity region is larger than the shear gradient in the bulk (Figure 25). When extrapolating the linear portion of the velocity profile to *v*(*z*) = 0, the intercept is negative. The negative intercept is the “slip length”, $b_{sl}$ [109]. The reverse situation with a near-surface viscosity higher than the bulk viscosity causes a positive intercept, which may be viewed as the hydrodynamic thickness of the respective adsorbate. 

More quantitatively, the slip length is defined as:

(67) $$b_{s1}=\int_{0}^{\infty }\left(\frac{\eta_{\mathrm{bulk}}}{\eta \left(z\right)}-1\right)\;dz$$

Slip of this kind might appear as exceptional because the density of a liquid close to a wall tends to be larger than the density of the bulk due to the attractive forces exerted by the wall. The question has caused much debate, but the evidence for slip in special situations has now solidified [110]. These situations include water at hydrophobic surfaces [111] and various flows inside hollow carbon nanotubes [112]. The slip length typically is of the order of a few diameters of the respective molecules, meaning, a few nanometers. Experimentally determining the slip length therefore is a challenge. At this point, the QCM plays out an advantage, which is the small depth of penetration of the shear wave. To the QCM, slip looks like an apparent negative Sauerbrey thickness. A Sauerbrey thickness (positive or negative) is easily determined with an accuracy of 1 nm or better. This being said: The slip length as determined with the QCM is different from $b_{s1}$ as defined in Equation (67). Following Equation (59), the negative Sauerbrey thickness (termed $b_{s1,\mathrm{ac}}$ here, “*ac*” for acoustic) is:

(68) $$b_{s1,\mathrm{ac}}=\int_{0}^{\infty }\left(\frac{\eta_{\mathrm{bulk}}}{\eta \left(z\right)}-\frac{\rho \left(z\right)}{\rho_{\mathrm{bulk}}}\right)\;dz\;$$

For the QCM, the density enters. The density of a slipping layer may be lower than the density of the bulk if slip is caused by nanobubbles or nanopancakes [113]. There are two more complications with nanobubbles:

- Nanobubbles constitute a sample with lateral structure, while Equation (68) assumes lateral homogeneity.
- This discussion ignores the surface energy of air-water interfaces (between the nanobubbles and the bulk liquid). Surface tension does play a role on the nanoscale. Surface tension turns nanobubbles into stiff objects [114,115]. (For macroscopic droplets or bubbles, the surface energy does not affect the resonance frequency because the associated oscillatory capillary pressure is small compared to the viscous stress.)

## 5. Non-Planar Samples

### 5.1. Point Contacts with Large Objects Clamped in Space by Inertia

By touching the resonator with a sharp tip, one increases the resonance frequency. This was first recognized by Dybwad in 1985 [116]. On an elementary level, the increase can be explained with the relation $\omega_{0}{\left(\kappa_{R}{/m}_{R}\right)}^{1/2}$. When operated in the gravimetric mode, $\kappa_{R}$ is about constant and the added mass lets the frequency decrease. When the resonator is touched with a tip (or with a sphere), the restoring force exerted by the contact lets the effective stiffness increase more strongly than the effective mass, thereby increasing the frequency. 

The small-load approximation makes this understanding more quantitative. Within the parallel-plate model, the load impedance of a point contact is:

(69) $${\tilde{Z}}_{L,\mathrm{elas}}=\frac{n_{P}}{A_{\mathrm{eff}}}\frac{\hat{F}}{{\hat{v}}_{S}}=\frac{n_{P}}{A_{\mathrm{eff}}}\frac{{\tilde{\kappa }}_{P}{\hat{u}}_{S}}{i\omega {\hat{u}}_{S}}=\frac{n_{P}}{A_{\mathrm{eff}}}\frac{{\tilde{\kappa }}_{P}}{i\omega }\;$$

$n_{P}$ is the number of contacts and $A_{\mathrm{eff}}$ is the plate’s effective area. The subscript *elas* stands for elastic loading (to be distinguished from inertial loading). The transverse stress is replaced by the transverse force, $\tilde{F}$, multiplied by the number density of the particles, $n_{P}$/$A_{\mathrm{eff}}$. The contact is modeled as a Hookean spring $\left(F={\tilde{\kappa }}_{P}{\hat{u}}_{S}={\tilde{\kappa }}_{P}{\hat{v}}_{S}/(i\omega )\right)$ with ${\hat{u}}_{S}$ the displacement). The spring constant, ${\tilde{\kappa }}_{P}$, can be complex, in which case the contact increases the bandwidth. The term ${\tilde{\kappa }}_{P}/i\omega$ is the mechanical equivalent of a capacitor’s electrical impedance, which is 1/(iω*C*). Within Mindlin-theory, the contact stiffness of a sphere-plate contact is ${\tilde{\kappa }}_{P}=2\tilde{G}*r_{C}$ with $\tilde{G}*$ an effective modulus, similar in magnitude to the shear modulus, and $r_{C}$ the contact radius [117]. 

**Figure 26:**
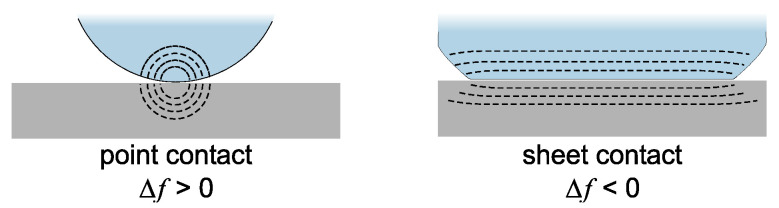
Point contacts and sheet contacts are characterized by spherical waves and planar waves, respectively.

The contact stiffness results from a small-scale deformation in the immediate vicinity of the contact. The deformation involves both the external object and the substrate (close to the contact). The relation ${\tilde{\kappa }}_{P}=2\tilde{G}*r_{C}$ requires a small contact area, that is, a “point contact“. Contacts are small if the displacement pattern has spherical symmetry and decays as about ${1/r}^{2}$ with *r* the distance from the contact. This requires $r_{C}$ to be much smaller than the local radius of curvature of the external object (often called *R*) and it also requires $r_{C}$ to be much smaller than the wavelength of sound, λ. In the opposite limit of $r_{C}\gg$ λ (“sheet contact” in Figure 26) the resonator launches a plane wave into the external object and $\Delta \tilde{f}$ should be analyzed with Equation (34).

Inserting the load from Equation (69) into the small-load approximation leads to:

(70) $$\Delta f+i\Delta \Gamma =\frac{1}{{2n\pi }^{2}Z_{q}}\frac{n_{P}}{A_{\mathrm{eff}}}\left({\kappa_{P}}^{\prime }{{+i\kappa }_{P}}^{\prime \prime }\right)\;$$

where Δ*f* is positive and scales as 1/*n*, which is indeed observed [118]. The contact stiffness as derived with Equation (70) was compared to the expectations resulting from JKR theory in [119].

Equation (70) neglects effects of added mass. It also assumes that the object supporting the contact from the back is fixed in space. This assumption is reasonable for contacts with sufficiently large spheres (*R* ≈ 200 µm). These are clamped by inertia.

### 5.2. Large Amplitudes, Partial Slip

Many piezoelectric devices can act as both sensors and actuators. The piezo effect works in both directions. The QCM, on the contrary, rarely is an actuator. Concerning static actuation, the piezoelectric coefficient of quartz is small compared to the competing materials such as lead zirconate titanate, PZT. One might still hope that the vibration would take some effect. A typical application would be sonolubrication [120,121,122]. Sliding of powders induced by a QCM vibrating at large amplitude has been studied experimentally in [123]. Generally speaking, kHz vibrations are better suited to sonolubrication than MHz waves [120]. 

Amplitude effects have been studied with the QCM on a few occasions. An incomplete list follows:

- Unbinding of virus particles at high amplitudes was studied in [124].
- Adsorption was prevented at high amplitudes in [125] and other publications by the same group.
- Cell adhesion as a function of amplitude was studied in [126]. Cell adhesion was delayed by high amplitudes, but cells, which had already adhered, did not detach when shaken vigorously.
- High amplitudes can induce steady streaming, as shown in [127]. More generally, the Reynolds number at high amplitude can be large enough to let the nonlinear term in the Navier-Stokes equation (of the form ρ(v·∇)v) be significant. This term can cause an oscillatory Bernoulli pressure. There may be a net attractive force onto colloidal particles, mediated by a high-frequency version of the Magnus force [128].

The following text is concerned with a quantitative discussion of nonlinearities in high-frequency contact mechanics as evidenced in QCM experiments. For linear resonators (also: “simple harmonic resonators”, SHOs), the resonance frequency is independent of amplitude. A “linear resonator” is characterized by the restoring force being proportional to displacement. An example for a slightly nonlinear resonator is the pendulum. The restoring force is $m_{R}$*g* sin(θ), where *g* = 9.81 m/s^2^ is the earth’s gravitational acceleration and θ is the angle of swing. If θ ≪ 1, one has sin(θ) ≈ θ and the pendulum operates as a linear resonator. The “grandfather clock” (named that way in Wikipedia) employs a long pendulum in order to ensure small angles. The amplitude is constant. Should the amplitude become large, this will decrease the clock’s frequency. The effect can also be observed, watching children on a swing. 

Speaking of clocks, the current quartz clocks are slightly nonlinear, as well. The stress inside a deformed quartz plate is not strictly proportional to the strain. The deviations are small, but they are noticed when driving quartz clocks with too much electrical power. The problem carries the name “drive level dependence”, DLD [129]. The DLD constrains the maximum voltage when interrogating the crystal’s resonance properties. The nonlinearity is such that the resonance frequency increases quadratically with the driving voltage. The DLD is of considerable concern in time and frequency control. Actually, the nonlinear elasticity of crystalline quartz is *only* of concern in this context because it can only be measured based on the resonance frequency. This is an example, where frequency-based metrology plays out its strength.

A nonlinear force displacement relation may also originate from the sample. This is not usually the case with films, with liquids, or with adsorbates from the liquid phase because the amplitude is too small. Typical amplitudes are a few nanometers at most (Section 8.5). With a penetration depth of 200 nm, the shear angle is less than 1%. Shear gradients of this magnitude typically are in the linear regime (stress proportional to strain). This is good news in some ways (no need to worry) and is a disappointment in others. Nonlinear rheology is of much interest in polymer science, but high-frequency rheology on polymer films using the QCM [12] is bound to be linear rheology. 

**Figure 27:**
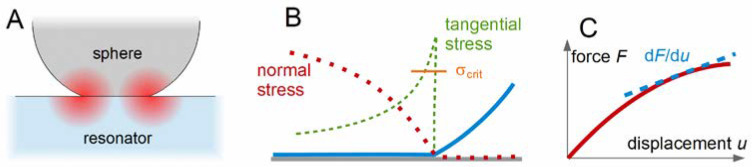
At the edge of a contact under a transverse load (red in (**A**)) one often finds a sharp peak in transverse stress (**B**). If the local stress exceeds a certain critical stress, sliding sets in. This is referred to as partial slip [131]. Panel (**C**) sketches the force-displacement relation of a sphere-plate contact with partial slip as calculated with the Cattaneo-Mindlin model [132]. The differential contact stiffness is indicated as a dashed line. It decreases with increasing displacement.

Nonlinear force displacement relations are commonplace in contact mechanics [118,130]. In contact mechanics, the local stress at the points of contact is large. Also, the contact area can vary in response to the load. Nonlinear high-frequency contact mechanics *can* be studied with the QCM. The experiments are rather simple. Frequency and bandwidth are determined as a function of amplitude. Figure 27 sketches one particular mechanism (partial slip), which softens contacts at large amplitudes. Partial slip lets the resonance frequency decrease.

When rough surfaces make contact, the stress distribution is strongly heterogeneous. The stress is large at the tips of the asperities, giving rise to nonlinear force-displacement relations and even plastic flow, also called “asperity creep” [133]. Less well known is the heterogeneous stress distribution at contacts between smooth spheres and plates (more generally, at the edges of extended contacts between smooth surfaces). When these contacts are sheared or loaded in some other way, a stress singularity develops at the edge. A similar singularity exists at crack tips in fracture mechanics. The stress—under certain conditions—scales as *r*^−1/2^ with *r* the distance from the crack tip. The open wedge outside the sphere-plate contact in Figure 27B can be viewed as a crack.

When the resonator exerts a periodic transverse force onto a sphere-plate contact, the stress at the edge may be so high that the contact starts sliding at the edge. Partial slip is useful when heavy objects hit piles of gravel. Such piles rarely fall over because the energy of the impact is dissipated efficiently in partial slip (and, also, in gross slip, which is still local) [134]. Partial slip can be detrimental in engineering. Contacts, which have seen prolonged vibrations, may suddenly fail because partial slip has turned into what is called “fretting wear” [135].

**Figure 28:**
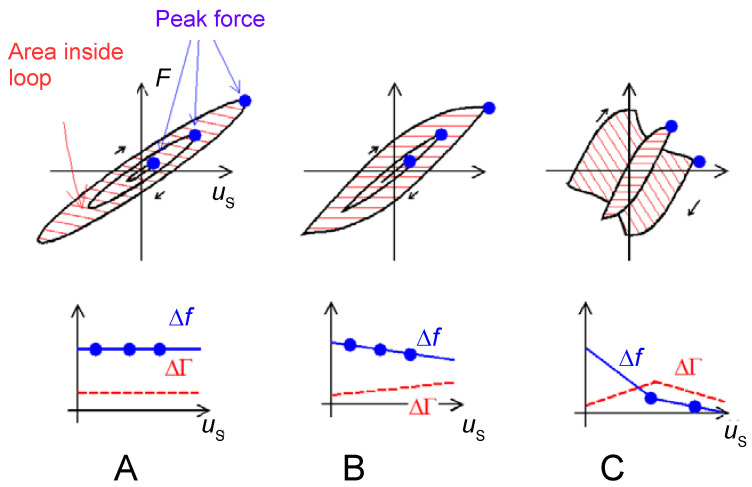
The top panels show friction loops for three different contacts. ΔΓ is strictly proportional to the area inside the loop, divided by $u_{S}^{2}$. Δ*f* is nearly proportional to the maximum force divided by the maximum displacement (blue dots). The left-hand side (**A**) shows the linear viscoelastic contact. Δ*f* and ΔΓ are constant. For partial slip (**B**) the ellipse-shaped loop turns into a lens-shaped loop. The area inside the loop increases with amplitude as $u_{S}^{3}$ and ΔΓ increases with amplitude, in consequence. The sketch in (**B**) is based on a quantitative model. The right-hand side (**C**) sketches the transition to gross slip. The diagram is motivated by experimental results [141], not by a quantitative model. ΔΓ decreases at large amplitude because the friction force in steady sliding weakly depends on velocity. Dividing by $u_{S}^{2}$ causes ΔΓ to decrease with $u_{S}$.

Partial slip was modelled in the 1950s by Mindlin [131], building on earlier work by Cattaneo [136]. The calculation leads to a friction loop as shown in Figure 28B (a lens-shaped loop, rather than an ellipse). The target is to turn this function into a prediction for Δ*f* and ΔΓ as a function of oscillation amplitude. The oscillation amplitude will be called $u_{S}$ in the following (no hat, because its complex nature is unessential). One may guess that Δ*f* and ΔΓ will decrease and increase with $u_{S}$, respectively, because the force-displacement relation in Figure 27C bends downward and because energy is dissipated in sliding. The Cattaneo-Mindlin model will confirm that. Further, it will predict these changes to be proportional to amplitude. 

Some background is needed. If the relation between stress and displacement is nonlinear, the area average (always inherent to the small-load approximation) must be complemented by a time average, following:

(71) $$\Delta f\left(u_{S}\right)+i\Delta \Gamma \left(u_{S}\right)=\frac{n_{P}}{A_{\mathrm{eff}}}\frac{1}{{2n\pi }^{2}Z_{q}}\frac{2}{u_{S}}{\langle F\left(t\right)\exp\left(i\omega t\right)\rangle }_{\mathrm{time}}$$

The derivation of Equation (71) makes use of the two-timing approximation [137]. As in Equation (69), stress was replaced by the transverse force acting onto the contact, *F*(*t*), normalized to area. *F*(*t*) is assumed to be periodic with the frequency of excitation, but not necessarily time-harmonic. If *F*(*t*) is time-harmonic, Equation (71) reduces to Equation (70). In the calculation of *F*(*t*) from the force-displacement relation, it is assumed that the motion of the substrate is close to time-harmonic (of the form cos(ω*t*)). This type of displacement control (rather than force control or mixed control) is inherent to the small-load approximation.

Relations similar to Equation (71) are exploited in scanning force microscopy [138]. In that context, the function *F*(*z*) (with *z* the distance between the tip and the surface) can be explicitly reconstructed from the resonance properties of the cantilever as a function *z*. Such an explicit reconstruction of a force-displacement relation is not possible for partial slip. Partial slip is hysteretic; hysteresis violates the conditions needed for the explicit reconstruction.

Again, the force-displacement relation cannot be explicitly derived from $\Delta \tilde{f}(u_{S})$ but certain models can be formulated and can be used to predict the functions Δ*f*($u_{S}$) and ΔΓ($u_{S}$), which can be checked against experiment. Figure 28 sketches three such models. For the viscoelastic contact (Figure 28A), Δ*f* and ΔΓ are independent of amplitude. Figure 28B shows the prediction from the Cattaneo-Mindlin model for partial slip [131]. (There are other models of partial slip, one of them described in [139].) Figure 28C depicts the transition to gross slip [140]. When gross slip sets in, ΔΓ decreases at large amplitudes and Δ*f* levels off to a small value.

The following discussion is concerned with partial slip (Figure 28B). In order to calculate Δ*f* and ΔΓ from Equation (71), the function *F*(*t*) must be derived from the functions *F*_→_($u_{N}$,$u_{S}$,ω) and *F*_←_($u_{N}$,$u_{S}$,ω) as predicted by the Cattaneo-Mindlin model. (For their algebraic form, see, for instance, [132].) The parameter $u_{N}$ is the displacement normalized to the peak displacement, $u_{S}$. The subscripts → and ← denote increasing and decreasing *u*_N_. Because these two forces are different, the force-displacement relation forms a loop. 

The calculation leads to [141]:

(72) $$\Delta f\left(u_{S},\omega \right)=\alpha \;\int_{-1}^{1}\left(F_{\to }\left(u_{N}{,u}_{S},\omega \right){+F_{\leftarrow }}_{}\left(u_{N}{,u}_{S},\omega \right)\right)\frac{u_{N}}{\sqrt{{1-u}_{N}^{2}}}{du}_{N}\;\Delta \Gamma \left(u_{S},\omega \right)\;=\alpha \;\int_{-1}^{1}\left(F_{\to }\left(u_{N}{,u}_{S},\omega \right)\;{-F_{\leftarrow }}_{}\left(u_{N}{,u}_{S},\omega \right)\right)\;{du}_{N}\;\alpha =\frac{n_{P}}{A_{\mathrm{eff}}}\cdot \frac{1}{{2n\pi }^{2}Z_{q}}\cdot \frac{1}{u_{S}}\cdot \frac{1}{\pi }\;$$

The term $u_{N}{\left({1-u}_{N}^{2}\right)}^{1/2}$ in line 1 can be viewed as statistical weight. Δ*f* is proportional to a weighted average of |*F*_→_ + *F*_←_|, where the weight function has a sharp peak at the turning point. Following this argument, Δ*f* is roughly proportional to the force-displacement ratio at maximum displacement (blue dots in the top row in Figure 28). Following line 2, ΔΓ is proportional to the area inside the friction loop, divided by $u_{S}^{2}$ (One divides by $u_{S}^{2}$ because d$u_{N}$ in Equation (72) is equal to d(*u*/$u_{S}$).) The latter result is exact, there are no approximations involved. 

For partial slip following Cattaneo and Mindlin, the calculation leads to [141]:

(73) $$\Delta f\left(u_{S}\right)\;\approx \;\frac{n_{P}}{{2n\pi }^{2}A_{\mathrm{eff}}Z_{q}}{\kappa_{P}}^{\prime }\left(1-\frac{1}{3}\frac{{\kappa_{P}}^{\prime }}{{\mu F}_{N}}u_{S}\right)\;\Delta \Gamma \left(u_{S}\right)\approx \frac{n_{P}}{{2n\pi }^{2}A_{\mathrm{eff}}Z_{q}}{\kappa_{P}}^{\prime \prime }\left(1+\frac{4}{9\pi }\frac{{\kappa_{P}}^{\prime }}{{\mu F}_{N}}u_{S}\right)\;$$

${\tilde{\kappa }}_{P}$ is the contact stiffness in the low-amplitude limit. *F*_N_ is the normal force. The model’s one free parameter is µ (the “friction coefficient”), which is the ratio of the critical tangential stress for sliding to the normal stress. µ should not be naively identified with the friction coefficient in macroscopic sliding. It turns out, though, that µ is of order unity, similar to the conventional friction coefficient [119]. 

Equation (73) was found to apply in some experiments [141,142]. Others rather show a quadratic dependence, which actually was predicted by another model of partial slip, put forward by Savkoor [139]. Still other experiments (mostly on granular media) show an increase of Δf with $u_{S}$, which can be explained with shear stiffening. 

There is a caveat: Equation (73) results from an integration over the friction loop. All models making use of Equation (71) involve such an integration. The shape of the friction loop cannot be inferred from the dependence of Δ*f* and ΔΓ on amplitude. Also, the force-displacement relation leading to Equation (73) was derived assuming a quasistatic situation. The dynamics at MHz frequencies might be different. Even a response, which is linear on the sub-μs time scale and therefore leads to an elliptical friction loop (Figure 28A), can produce an amplitude dependence of Δ*f* and ΔΓ if the width and angle of the ellipse depend on amplitude. 

### 5.3. Structured Samples, Numerical Calculations

**Figure 29:**
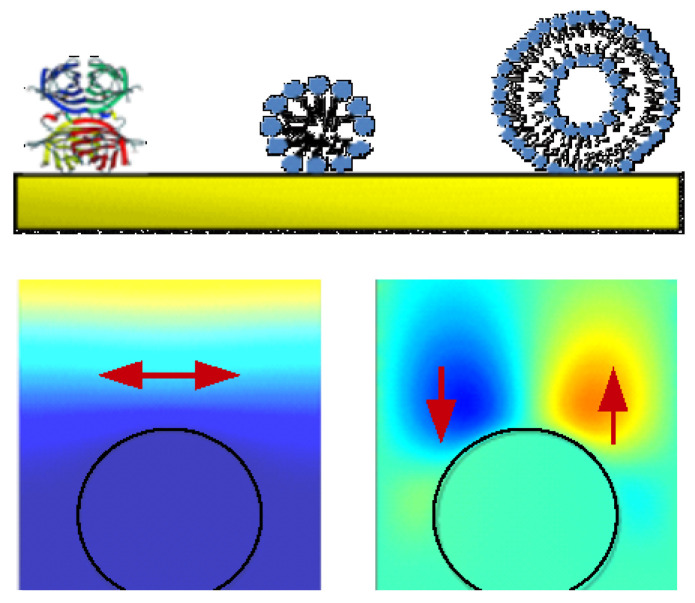
Top: Typical objects to be modeled numerically are adsorbed particles of various kinds (proteins, micelles, vesicles, not drawn to scale). Bottom: Results from a finite element method (FEM) simulation in 2D. Left: tangential velocity. Right: normal velocity. The truncated cylinder is rigid. The simulation outputs the periodic stress at the resonator surface (not shown), which can be converted to $\Delta \tilde{f}$ with Equation (25).

Many samples of interest in soft matter physics have some in-plane structure (Figure 29). This includes proteins [143], dendrimers [144], biological cells [145], and colloidal particles [146,147]. Adsorbed vesicles, which may or may not rupture and flatten out into supported lipid bilayers (SLBs), have been studied in much detail [148,149,150]. 

Predicting shifts in frequency and bandwidth induced by such samples from the structure and the viscoelastic parameters requires a numerical model. As long as the area average in Equation (25) can be applied, the shifts in frequency and bandwidth can be computed numerically. The input to the small-load approximation is the area-averaged amplitude of the periodic stress at the resonator surface. The stress field at the surface can be extracted from a solution of the equations of continuum viscoelasticity. 

The medium obeys the Stokes equation expressed in the frequency domain, which is:

(74) $$i\omega \hat{v}(r)=\hat{v}(r)\nabla^{2}\hat{v}(r)-\frac{1}{\rho }\nabla \hat{p}(r)$$

where $\tilde{v}=\tilde{\eta }/\rho$ is the kinematic viscosity. The pressure, $\hat{p}$, follows from the modulus of compression, $\tilde{K}$, and the divergence of the velocity field as:

(75) $$\hat{p}(r)=\frac{-\tilde{K}(r)}{i^{\;}\omega }(\nabla \cdot \hat{v}(r))$$

The density was assumed as constant in Equation (74), but may also depend on position. The complex dynamic viscosity, $\tilde{\eta }(r)$, can take any value. Elastic objects have η’’ ≫ η’. Rigid objects are represented as objects with large $|\tilde{\eta }|\cdot$ or, equivalently, with small ${\tilde{\eta }}^{-1}$. Such objects are hardly deformed by the shear stress. For rigid objects, the question of viscous or elastic response does not occur, because it does not matter whether a negligible deformation occurs in-phase or out-of-phase to the stress. This argument reiterates the previous statement that the nontrivial samples to the QCM are the soft samples. 

The Stokes equation is a linearization of the Navier-Stokes equation. The Reynolds number is assumed to be so small that the nonlinear term $\left(\rho (\hat{v}\cdot \nabla )\hat{v}\right)$ in the Navier-Stokes equation can be neglected. Actually, effects of finite Reynolds number *can* be seen in QCM experiments at high amplitude. Because these effects are weak, they can be modeled, based on the algorithms described below. Call the solution to the linear problem ${\hat{v}}^{\left(0\right)}$. The nonlinear term then generates a Bernoulli pressure of the form $\rho \left({\hat{v}}^{\left(0\right)}\cdot \nabla \right)v^{\left(0\right)}$. This pressure vanishes for pure shear flow because it contains the dot-product of the velocity and its gradients. It does not vanish for structured samples. It occurs at ω = 0 (steady streaming) and at 2ω (2nd harmonic generation) because of the relation $\cos^{2}\left(\omega t\right)\;=\;1/2\left(1\;+\;\cos\left(2\omega t\right)\right)$. The Bernoulli pressure drives a weak 2nd-order flow, which can be computed from ${\hat{v}}^{\left(0\right)}$.

**Figure 30:**
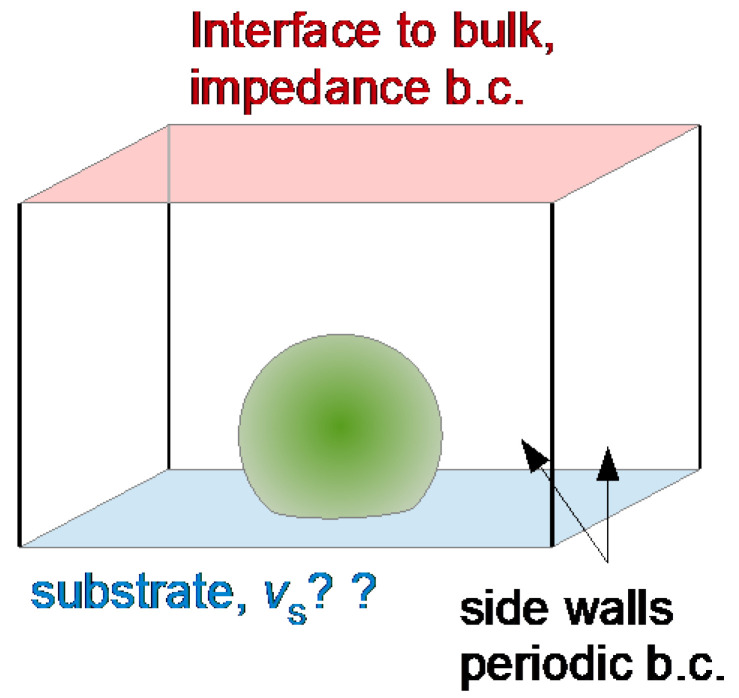
An example of a simulation box. An impedance boundary condition should be applied at the top in order to let the simulation volume be small.

Figure 30 sketches a simulation volume. It is a few tens of nanometers wide and contains a few adsorbed particles (if this is the problem of interest). Periodic boundary conditions (“b.c.”) apply at the side walls. A Dirichlet boundary condition applies at the bottom $\left(\hat{v}(z=0)=\left({\hat{v}}_{S},0,0\right)\right)$ The boundary condition at the top should be an impedance boundary condition (also: “Robin boundary condition” [151]), meaning that the ratio of the velocity gradient to the velocity should be such that the stress-velocity ratio inside the boundary is equal to the wave impedance outside the boundary.

There is ongoing research turning these concepts into practical and efficient algorithms. Computational techniques applicable to this problem include the finite element method (FEM) [152,153], the finite volume method (FVM) [154], and the Lattice-Boltzmann method (LBM) [155,156]. 

If the sample is much thinner than the wavelength of sound, it may look to the QCM like a film. The QCM then does not actually recognize the structure. One may infer an apparent mass, ${\tilde{m}}_{\mathrm{app}}$, and an apparent compliance (${J_{\mathrm{app}}}^{\prime },\;{J_{\mathrm{app}}}^{\prime \prime }$) from plots of Δ*f*/*n* and ΔΓ/*n* versus *n* (as in Figure 21). The problem to the experimentalist is to interpret these parameters. For instance, the apparent mass is different from the true mass because a certain amount of liquid, which is trapped in the space between the particles, takes part in the resonator’s motion [157,158,159]. Likewise, the apparent compliance has a contribution from the flow of the liquid around the particles.

The numerical simulation calculates Δ*f* and ΔΓ from a known structure. The task to the experimentalist usually is the reverse problem, namely inferring the structure from experimentally determined values {Δ*f*/*n*, ΔΓ/*n*} is another. The reverse problem is underdetermined, in general. The formalism can only run backwards, if the structure is known to a significant extent with few free parameters, so that the experimental values of {Δ*f*/*n*, ΔΓ/*n*} can be exploited to determine these parameters. Such a situation is (for example) encountered, when rigid spheres of known size adsorb to the QCM surface, following random sequential adsorption [144]. If coverage can be estimated independently, the only free parameter of this problem is the stiffness of the sphere-plate contacts. Arguably, contact stiffness involves a total of four free parameters, which are the real and the imaginary parts of the shear stiffness, ${\tilde{\kappa }}_{\mathrm{Sh}}$, and the bending stiffness, ${\tilde{\kappa }}_{b}$ (related to translation and rotation of the sphere). For spheres in air, the problem is amenable to an analytical treatment (Section 6.2). In liquids, hydrodynamic comes into play. For this problem, a calibration curve relating contact stiffness to the sets of {Δ*f*/*n*, ΔΓ/*n*} can be obtained from simulation. These calibration curves can be used to analyze experiments. A related problem is the fractional trapped mass as a function of coverage. The trapped mass may also be estimated from calibration curves obtained with simulations on structures, which are well-defined, on the one hand, but still reasonably close to experiment, on the other. 

### 5.4. Roughness

Roughness in QCM experiments is always a reason to worry. Gold surfaces prepared by physical vapor deposition (PVD) have an rms roughness of about 1 nm. Spin-cast polymer films usually are considered as smooth. One might model roughness effects numerically as in Section 5.3, but there is a wide range of possible geometries.

We limit the discussion to small-scale, shallow roughness as modelled analytically in [160,161]. The vertical scale of roughness is assumed to be smaller than the lateral scale, which is realistic for gold surfaces prepared by PVD. The lateral scale is assumed to be smaller than δ, which again often is realistic. The model has two free parameters, which are the vertical scale, $h_{r}$, and the lateral scale, $l_{r}$. One might also use the vertical scale, $h_{r}$, and the aspect ratio, $h_{r}$/$l_{r}$. The aspect ratio is assumed to be smaller than unity (“shallow roughness”). 

There is an interesting experimental statement in the literature, which is that the bandwidth is less affected by small-scale roughness than the frequency [162]. This finding is corroborated by the analytical treatment of shallow roughness following [161]. These authors Fourier-decompose the height profiles into sinusoidal corrugation waves with wave vector *q*. They solve the hydrodynamics problem for the different Fourier components, separately, and calculate Δ*f* and ΔΓ. The total frequency shift follows from integration over all wave vectors, where the weight function is the square of the respective amplitude. A Gaussian distribution is assumed, the center of which is much larger than the inverse penetration depth (“small-scale roughness”). The following equations are obtained:

(76) $$\begin{array}{l} \frac{\Delta f}{f_{0}}=\frac{1}{{\pi Z}_{q}}\sqrt{\frac{\omega \rho \eta }{2}}\left(1+3\sqrt{\pi }\frac{h_{r}}{l_{r}}\;\frac{h_{r}}{\delta }-2{\left(\frac{h_{r}}{\delta }\right)}^{2}\right)\\ \frac{\Delta \Gamma }{f_{0}}=\frac{1}{{\pi Z}_{q}}\sqrt{\frac{\omega \rho \eta }{2}}\left(1+2{\left(\frac{h_{r}}{\delta }\right)}^{2}\right)\;\; \end{array}$$

Equation (76) can be reorganized as:

(77) $$\frac{\Delta f+i\Delta \Gamma }{f_{0}}=\frac{i}{{\pi Z}_{q}}\sqrt{i\omega \rho \eta }\left[1\;-\;2i{\left(\frac{h_{r}}{\delta }\right)}^{2}\right]\;\;\frac{1}{{\pi Z}_{q}}{\omega \rho h}_{r}\frac{3\sqrt{\pi }}{2}\frac{h_{r}}{l_{r}}\;$$

The term in square brackets modifies the Gordon-Kanazawa term and it does so in proportion to $h_{r}^{2}$. This term affects both frequency and bandwidth. The second term only affects the frequency shift. It can be thought of as a Sauerbrey-like term, covering trapped mass. In principle, this terms scale as $h_{r}^{2}$, as well. However, if the aspect ratio is constant, one factor of $h_{r}$ is absorbed in the aspect ratio (given as $h_{r}{/l}_{r}$), which leaves the other $h_{r}$ as a linear term. Roughness is often created in a way, which leaves the aspect ratio constant. The amount of trapped mass then depends linearly on the cluster size. The authors of [162] did experiments of that kind. Because effects in bandwidth are proportional to $h_{r}^{2}$, they are hardly seen at small roughness.

## 6. Coupled Resonances

### 6.1. The Sphere with Moderate Mass

One example of a coupled resonance (the film resonance) was already discussed in Section 4.5.4. Here, we start from another example, which is the sphere attached to the resonator. (Particle adsorption to a QCM was discussed as early as 1971 in [163].) Contrasting to Section 5.1, the sphere has moderate size. It is neither clamped in space by inertia (elastic loading), nor is it a nanoparticle in the sense, that it would be rigidly attached to the resonator and constitute a Sauerbrey load.

**Figure 31:**
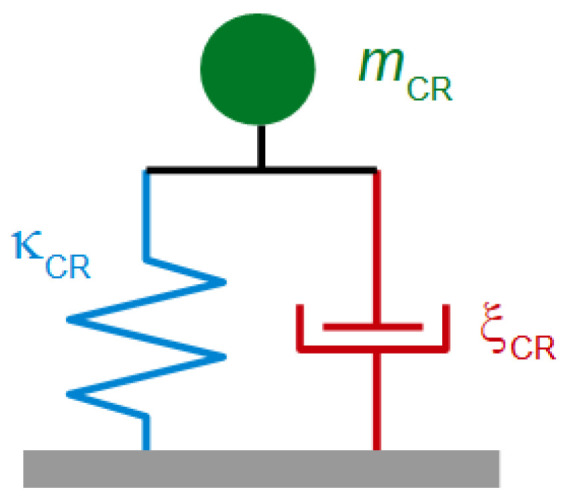
A lumped-element representation of a load giving rise to a coupled resonance. This mechanical circuit implicitly invokes the small-load approximation insofar, as the resonator has been drawn as a plate. It might also have been drawn as two large spheres, coupled to each other by another spring (Figure 1B). Had it been drawn as in Figure 1B, the solution to the full set of dynamical equations would have predicted anticrossing (as for the film resonance, Section 4.5.4). Anticrossing is not further discussed in the following. Coupled resonances caused by particles will usually be too broad to let anticrossing be visible.

Figure 31 shows a lumped-element circuit. The link between the particle and the resonator is depicted as a spring with stiffness $\kappa_{\mathrm{CR}}$. A dashpot with drag coefficient $\xi_{\mathrm{CR}}$ was placed in parallel to the spring, accounting for dissipative processes. The subscript *CR* denotes the coupled resonance. Written that way, the circuit suggests that the spring constant and the drag coefficient were independent of frequency. They may well depend on frequency. The spring constant may also be written as a frequency-dependent complex parameter ${\tilde{\kappa }}_{\mathrm{CR}}(\omega )={\kappa_{\mathrm{CR}}}^{\prime }(\omega )+i{\kappa_{\mathrm{CR}}}^{\prime \prime }(\omega ).\;{\tilde{\kappa }}_{\mathrm{CR}}(\omega )$ then is a response function similar to $\tilde{G}(\omega ).\;{\tilde{\kappa }}_{\mathrm{CR}}(\omega )$ has different units, though (force/displacement rather than stress/strain). 

The elements in Figure 31 represent mechanical impedances, which are ratios of force to velocity (rather than stress to velocity). Mechanical impedances and the electromechanical analogy [164] are discussed in Box 3. In mechanics, the total impedance of two elements arranged in parallel is the sum of the two individual impedances, following the “mechanical Kirchhoff rules”. Application of these rules to the circuit in Figure 31 leads to:

(78) $$\begin{array}{cc} \frac{\hat{F}}{{\hat{v}}_{S}} & ={\left[\frac{1}{i\omega m_{\mathrm{CR}}}+\frac{1}{\frac{\kappa_{\mathrm{CR}}}{i\omega }+i\xi_{\mathrm{CR}}}\right]}^{-1}={\left[\frac{1}{i\omega m_{\mathrm{CR}}}+\frac{1}{\frac{{\tilde{\kappa }}_{\mathrm{CR}}}{i\omega }}\right]}^{-1}={\left[\frac{1}{i\omega m_{\mathrm{CR}}}+\frac{i\omega }{{\tilde{\kappa }}_{\mathrm{CR}}}\right]}^{-1}\\ & =\frac{i\omega m_{\mathrm{CR}}{\tilde{\kappa }}_{\mathrm{CR}}}{{\tilde{\kappa }}_{\mathrm{CR}}-\omega^{2}m_{\mathrm{CR}}}=i\omega m_{\mathrm{CR}}\frac{{\tilde{\omega }}_{\mathrm{CR}}^{2}}{{\tilde{\omega }}_{\mathrm{CR}}^{2}-\omega^{2}} \end{array}$$

where $\hat{F}$ is the transverse force exerted by the sphere onto the resonator surface. Inserting Equation (78) into Equation (25) leads to:

(79) $$\frac{\Delta f+i\Delta \Gamma }{f_{0}^{}}=-\frac{n_{P}}{A_{\mathrm{eff}}}\frac{\omega m_{\mathrm{CR}}}{\pi Z_{q}}\left[\frac{{\tilde{\omega }}_{\mathrm{CR}}^{2}}{{\tilde{\omega }}_{\mathrm{CR}}^{2}-\omega^{2}}\right]$$

where $n_{P}$ is the number of particles. 

**Figure 32:**
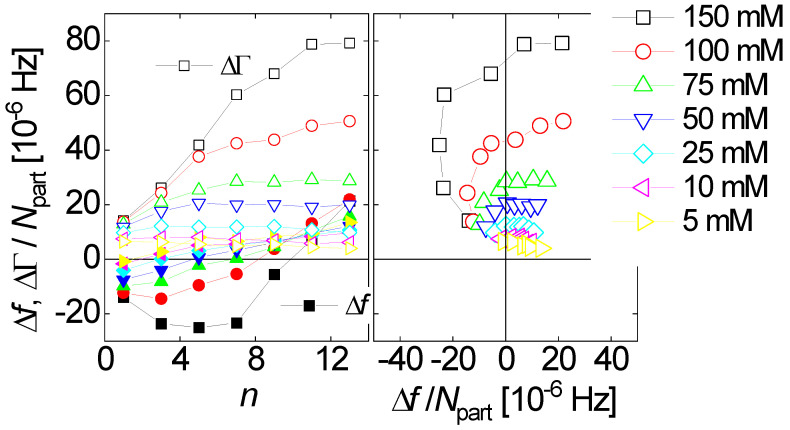
Shifts of frequency and bandwidth caused by the deposition of micron-sized silica spheres. The polar diagram on the right displays spirals, characteristic for the coupled resonance. The ion strength as indicated in the legend tunes the stiffness of the contact, where large ion strength leads to stiff contacts. Adapted from [167].

A side remark: One might be tempted to write ${\tilde{\omega }}_{\mathrm{CR}}^{2}-\omega^{2}$ as $\left({\tilde{\omega }}_{\mathrm{CR}}+\omega \right)\left(\omega_{\mathrm{CR}}-\omega \right)$ and $\left({\tilde{\omega }}_{\mathrm{CR}}+\omega \right)\approx 2{\tilde{\omega }}_{\mathrm{CR}}$ as in the mathematics leading to Equation (7). This is problematic because the coupled resonance is not usually a narrow resonance. For the same reason, polar diagrams of the complex frequency shift as in Figure 32 usually show spirals (as opposed to circles, Figure 3B).

**Figure 33:**
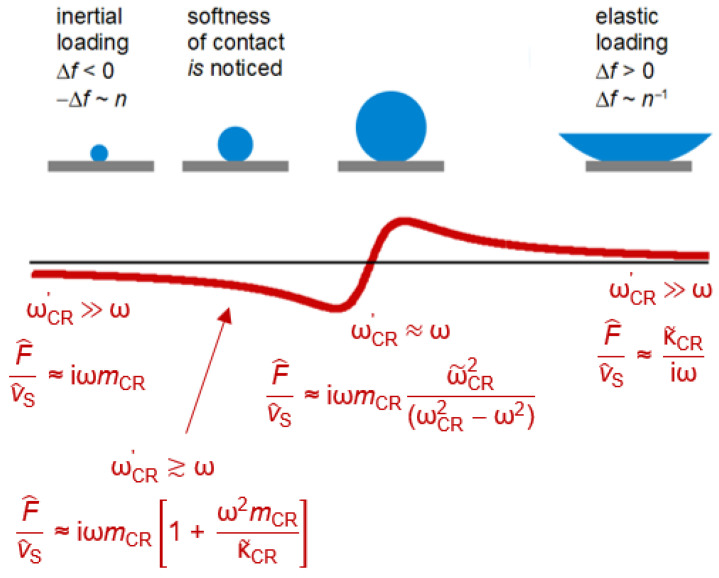
The frequency shift induced by a coupled resonance, following Equation (79). At low and high ω, the Sauerbrey limit and the elastic-load limit are recovered. The overtone scaling in these limits is −Δ*f*/*n* ≈ const and +Δ*f*·*n* ≈ const. In a range next to the Sauerbrey regime, the softness of the contact can be inferred from a non-trivial the overtone dependence of Δ*f*/*n* + iΔΓ/*n*.

Equation (79) contains the Sauerbrey case and the elastic-load case in the limits of $\omega \;\ll {{\;\omega }_{\mathrm{CR}}}^{\prime }$ and $\omega \;\gg {{\;\omega }_{\mathrm{CR}}}^{\prime }$, respectively (Figure 33). When $\omega \;\ll {{\;\omega }_{\mathrm{CR}}}^{\prime }$, the right-hand side in Equation (79) is about $-n_{P}{\omega m}_{\mathrm{CR}}{/(A}_{\mathrm{eff}}{\pi Z}_{q})$, which is equivalent to the Sauerbrey equation. When $\omega \;\gg {{\;\omega }_{\mathrm{CR}}}^{\prime }$, the right-hand side becomes $n_{P}/\left(A_{\mathrm{eff}}\pi Z_{q}\right)\left({\tilde{\kappa }}_{R}/\omega \right)$, which is the elastic load limit (Equation (69)).

There actually is a frequency range, in which the response is close to Sauerbrey-like, but in which the dynamics of the sphere is still seen as a small deviation from Sauerbrey behavior (compare to Figure 16 and Section 4.5.3). If ω is less but not much less than ${\omega_{\mathrm{CR}}}^{\prime }$ $\Delta \tilde{f}/n$ deviates from Sauerbrey-type behavior. This becomes evident when expanding Equation (79) to 3rd order in ω as:

(80) $$\frac{\Delta f+i\Delta \Gamma }{f_{0}^{}}=-\frac{n_{P}}{A_{\mathrm{eff}}}\frac{\omega m_{\mathrm{CR}}}{\pi Z_{q}}\left[1+\frac{\omega^{2}}{{\tilde{\omega }}_{\mathrm{CR}}^{2}}\right]=-\frac{n_{P}}{A_{\mathrm{eff}}}\frac{\omega m_{\mathrm{CR}}}{\pi Z_{q}}\left[1+\omega^{2}\frac{m_{\mathrm{CR}}}{{\tilde{\kappa }}_{\mathrm{CR}}}\right]$$

The parameters $m_{\mathrm{CR}}$ and ${\tilde{\kappa }}_{\mathrm{CR}}$ will need interpretation because the motion of the particle combines translation and rotation. $m_{\mathrm{CR}}$ and ${\tilde{\kappa }}_{\mathrm{CR}}$ are a modal mass and a modal stiffness. Section 6.2 elaborates on that problem.

The deviations from Sauerbrey behavior are proportional to the softness of the contact (the inverse contact stiffness). In this regard, Equation (80) is the analog to Equation (45), where the latter equation describes a soft film, rather than a soft link to a particle. 

When the frequency of the coupled resonance is in the range covered by the QCM, Δ*f* crosses from negative to positive [165,166]. An example is shown in Figure 32, adapted from [167]. The resonators were in contact with silica spheres with a radius of 2.5 µm. The spheres were attracted to the surface by gravity and by van-der-Waals forces. Both the spheres and the resonator surface carried negative charge, resulting in an electrostatic repulsion, which competes with the attractive forces. Variation of ion strength as indicated in the legend tuned this repulsive force [168]. Adding salt has two consequences. Firstly, the frequency of zero crossing increases. This can be understood as the consequence of increased contact stiffness, which in turn is the consequence of reduced electrostatic repulsion. Also, the radius of the semi-circle (more precisely, the semi-spiral) decreases. A closer look at Equation (79) shows that this radius is proportional to the ratio ${\kappa_{\mathrm{CR}}}^{\prime }{/\xi }_{\mathrm{CR}}$. Why electrostatic screening decreases this ratio is nontrivial.

Box 3 **The electromechanical analogy.**

Differing from electricity, mechanical impedances are additive when two elements are arranged in parallel. Inverse impedances are additive, when the elements are arranged in series. These are the mechanical Kirchhoff rules. In electricity, the reverse rules apply. Current (the analog of velocity) is additive, when two resistors are placed in parallel. For dashpots arranged in parallel, the forces (the analogs of voltage) are additive.

ElectricalMechanicalVoltage *U*Force *F*Current *I*Velocity *v*Resistor

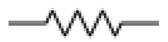

${\tilde{Z}}_{\mathrm{el}}=R$
Dashpot

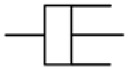

${\tilde{Z}}_{m}=\xi$
Capacitor

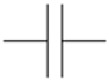

${\tilde{Z}}_{\mathrm{el}}=\frac{1}{i\omega C}$
Spring

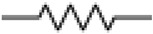

${\tilde{Z}}_{m}=\frac{\kappa }{i\omega }$
Inductor

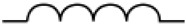

${\tilde{Z}}_{\mathrm{el}}=i\omega L$
Mass

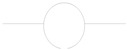

${\tilde{Z}}_{m}=i\omega m$
Elements in parallel
$\frac{1}{{\tilde{Z}}_{\mathrm{el},\;\mathrm{tot}}}=\frac{1}{{\tilde{Z}}_{\mathrm{el},\;A}}+\frac{1}{{\tilde{Z}}_{\mathrm{el},\;B}}$
Elements in parallel
${\tilde{Z}}_{m,\;\mathrm{tot}}={\tilde{Z}}_{m,\;A}+{\tilde{Z}}_{m,\;B}$
Elements in series
${\tilde{Z}}_{\mathrm{el},\;\mathrm{tot}}={\tilde{Z}}_{\mathrm{el},\;A}+{\tilde{Z}}_{\mathrm{el},\;B}$
Elements in series
$\frac{1}{{\tilde{Z}}_{m,\;\mathrm{tot}}}=\frac{1}{{\tilde{Z}}_{m,\;A}}+\frac{1}{{\tilde{Z}}_{m,\;B}}$
Ground*U* = 0Open end*F* = 0Open end*I* = 0Wall*V* = 0

### 6.2. Influence of Rotation on the Frequency Shift

Incorporating rotation into the formalism is worth the effort. Similar work was reported by Tarnapolsky et al. in [169]. Differing from the text below, these authors were concerned with large spheres (bacteria) in a liquid environment. The text below addresses small spheres (${\omega_{\mathrm{CR}}}^{\prime }\;≳\;\omega$) and avoids hydrodynamics by sticking to a dry environment. 

The sphere has two degrees of freedom, which are translation (with velocity ${\hat{u}}_{P}$) and rotation about the sphere center (with rate $\;\hat{\Omega }$). Inertia is balanced against the force and the torque exerted by the contact, ${\hat{F}}_{C}$ and ${\hat{M}}_{C}$, by the relations:

(81) $${i\omega m}_{P}{\hat{u}}_{P}\;=\;{\hat{F}}_{C}\;{i\omega m}_{P}\frac{2}{5}R^{2}\hat{\Omega }\;=\;{\hat{M}}_{C}\;$$

**Figure 34:**
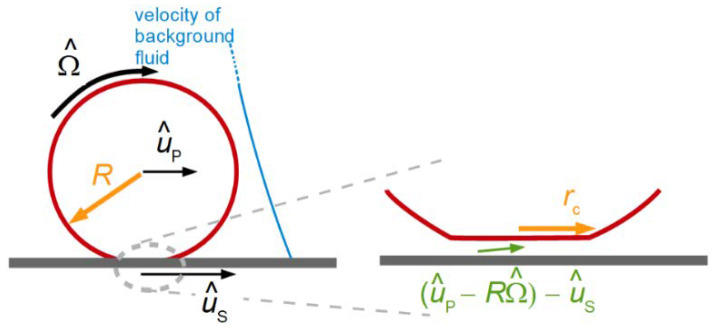
The sphere undergoes both translation and rotation. The transverse force at the contact is proportional to the displacement at the contact, which is different from the displacement of the sphere center.

The inertial terms are ${i\omega m}_{P}{\hat{u}}_{P}$ for translation and ${i\omega m}_{P}\left(2/5\right)R^{2}\hat{\Omega }$ for rotation. The subscript *P* denotes the particle. $m_{P}\left(2/5\right)R^{2}$ is the moment of inertia of a solid sphere rotating about its center. The transverse restoring force exerted by the contact is ${\hat{F}}_{C}\;=\;-{\tilde{\kappa }}_{\mathrm{Sh}}/(i\omega )\left(\left({\hat{u}}_{P}-R\hat{\Omega }\right){\hat{u}}_{S}\right)$. ${\tilde{\kappa }}_{\mathrm{Sh}}$ is the shear stiffness of the contact. Following Mindlin theory, ${\tilde{\kappa }}_{\mathrm{Sh}}$ is given as $2\tilde{G}{*r}_{C}$ where $\tilde{G}*$ is an effective modulus and $r_{C}$ is the contact radius. The term $\left({\hat{u}}_{P}-\;R\hat{\Omega }\right)-{\hat{u}}_{S}$ is the difference between the velocity of the sphere and the velocity of the substrate at the point of contact (Figure 34). 

The torque has two components. The first component is ${\hat{F}}_{C}R$. The second component follows from the contact’s bending stiffness. Following [169], we write the torque as ${\hat{M}}_{C}\;=-{\tilde{\kappa }}_{b}/\left(i\omega \right)R^{2}\hat{\Omega }$, where ${\tilde{\kappa }}_{b}$ is the bending stiffness. Defined this way, ${\tilde{\kappa }}_{b}$ has the same units as ${\tilde{\kappa }}_{\mathrm{Sh}}$. Defined this way, ${\tilde{\kappa }}_{b}$ is *not* a ratio of torque to angle (as elsewhere, the ratio of torque to angle here is ${\tilde{\kappa }}_{b}R^{2}$). Following Dominik and Tielens [170], the bending stiffness is:

(82) $${\tilde{\kappa }}_{b}\;=\;2{\tilde{\kappa }}_{\mathrm{Sh}}\frac{r_{C}^{2}}{R^{2}}\;$$

With these relations, the force and the torque are:

(83) $$\begin{array}{c} {\hat{F}}_{C}=-\frac{{\tilde{\kappa }}_{\mathrm{Sh}}}{i\omega }\left(\left({\hat{u}}_{P}-R\hat{\Omega }\right)-{\hat{u}}_{S}\right)\\ {\hat{M}}_{C}=-\frac{2{\tilde{\kappa }}_{\mathrm{Sh}}r_{C}^{2}}{i\omega }\hat{\Omega }-\frac{{\tilde{\kappa }}_{\mathrm{Sh}}R}{i\omega }\left(\left({\hat{u}}_{P}-R\hat{\Omega }\right)-{\hat{u}}_{S}\right) \end{array}$$

Inserting Equation (83) into (81) and reorganizing terms, the following equation system results:

(84) $$\begin{array}{c} \left(i\omega m_{P}+\frac{{\tilde{\kappa }}_{\mathrm{Sh}}}{i\omega }\right){\hat{u}}_{P}+\left(-\frac{{\tilde{\kappa }}_{\mathrm{Sh}}}{i\omega }R\right)\hat{\Omega }=\frac{{\tilde{\kappa }}_{\mathrm{Sh}}}{i\omega }{\hat{u}}_{S}\\ \left(\frac{{\tilde{\kappa }}_{\mathrm{Sh}}R}{i\omega }\right){\hat{u}}_{P}+\left(i\omega m_{P}\frac{2}{5}R^{2}+\frac{2{\tilde{\kappa }}_{\mathrm{Sh}}r_{C}^{2}}{i\omega }-\frac{{\tilde{\kappa }}_{\mathrm{Sh}}R}{i\omega }\right)\hat{\Omega }=\frac{{\tilde{\kappa }}_{\mathrm{Sh}}R}{i\omega }{\hat{u}}_{S} \end{array}$$

The terms in brackets in Equation (84) form a matrix, which must be inverted to obtain the vector (${\hat{u}}_{P}$,$\hat{\Omega }$) from the source term (${\tilde{\kappa }}_{\mathrm{Sh}}/\left(i\omega \right){\hat{u}}_{S},\;{\tilde{\kappa }}_{\mathrm{Sh}}R/\left(i\omega \right){\hat{u}}_{S}$). 

A resonance occurs, when (${\hat{u}}_{P}$, $\hat{\Omega }$) is nonzero even in the absence of a source term. The resonance frequencies are found by setting determinant of the coefficient matrix to zero. There are two resonance frequencies, because there are two dynamic variables. They are given as:

(85) $${\tilde{\omega }}_{\mathrm{CR},1,2}\;=\;\sqrt{\frac{{\tilde{\kappa }}_{\mathrm{Sh}}}{m_{P}}}\sqrt{A\;\pm \;B}\;A\;=\;\frac{7}{4}\;+\;\frac{5}{2}\frac{r_{C}^{2}}{R^{2}}\;\;\;\;\;\;B^{2}\;=\frac{49}{4}\;+\;15\frac{r_{C}^{2}}{R^{2}}\;+\;25\frac{r_{C}^{4}}{R^{4}}\sqrt{\frac{49}{4}\;+\;15\frac{r_{C}^{2}}{R^{2}}\;+\;25\frac{r_{C}^{4}}{R^{4}}}\;\;$$

Both modes combine translation and rotation, but one is predominantly translational, while the other is predominantly rotational. Note that the two resonance frequencies are not vastly different unless $r_{C}\;\ll \;R$. A search for two coupled resonances in experimental QCM data is difficult because the experiment must involve an ensemble of spheres and because there will be a distribution in the parameters ${\tilde{\kappa }}_{\mathrm{Sh}}$ and $r_{C}$. The heterogeneous line broadening caused by the distribution of parameters often lets the two resonances merge. 

The above remarks concerned situations, in which the frequency of the resonator is close to one of the frequencies of the coupled resonances. In general, the particles will be driven off-resonance. Setting ${\hat{u}}_{S}$ on the right-hand side in Equation (84) to unity fixes ${\hat{u}}_{P}$ and $\hat{\Omega }$ (to off-resonance values). The force, ${\hat{F}}_{C}$, follows from Equation (83). Inserting ${\hat{F}}_{C}$ into the small-load approximation yields Δ*f* and ΔΓ. Plotting Δ*f* and ΔΓ versus frequency, yields two resonances. This graph is not shown here because it looks very similar to Figure 36, which was obtained with a numerical simulation. 

In the following, we are concerned with small spheres, which are still large enough to show some dynamics. These spheres are almost Sauerbrey loads (are almost rigidly attached to the plate). Similar to Equation (80), we expand $\Delta \tilde{f}$ to 3rd order in ω. In Equation (80), the coefficient to $\omega^{2}$ was named $m_{\mathrm{CR}}/{\tilde{\kappa }}_{\mathrm{CR}}$ and it was left open, what these parameters mean. This gap can now be closed. The 3rd order expansion in ω of $\Delta \tilde{f}$ calculated from ${\hat{F}}_{C}$ leads to:

(86) $$\frac{\Delta f\;+\;i\Delta \Gamma }{f_{0}}\approx \frac{n_{P}}{A_{\mathrm{eff}}}\frac{{\omega m}_{\mathrm{CR}}}{{\pi Z}_{q}}\left[{1\;+\;\omega }^{2}\frac{m_{P}}{{\tilde{\kappa }}_{\mathrm{Sh}}}\left(1\;+\;\frac{R^{2}}{{2r}_{C}^{2}}\right)\right]\;\;$$

Clearly, $m_{\mathrm{CR}}$ is equal to $m_{P}$ (in this limit). With regard to contact stiffness, Equation (86) shows that ${\tilde{\kappa }}_{\mathrm{CR}}^{-1}$ from Equation (80) follows ${\tilde{\kappa }}_{\mathrm{CR}}^{-1}\;=\;{\tilde{\kappa }}_{\mathrm{Sh}}^{-1}\;+\;{\tilde{\kappa }}_{b}^{-1}$ (as also noted in [169]). 

**Figure 35:**
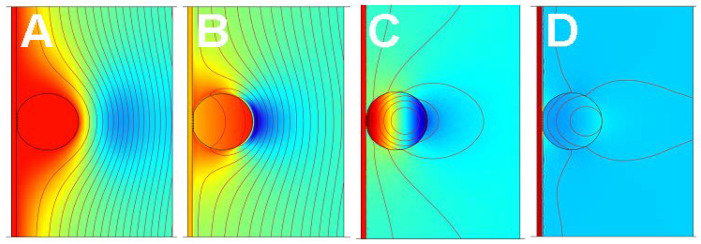
Velocity fields as determined with an FEM simulation in two dimensions. A cylinder attached to a plate gives rise to two coupled resonances. The diameter of the cylinder was 4 µm. The link to the substrate is 600 nm wide and 100 nm thick. Its shear modulus is 1 GPa. The colors encode the local tangential velocity. The frequencies are 1 MHz, 4.2 MHz, 54 MHz, and 90 MHz in panels (**A**–**D**), respectively. In panels (**A**,**C**), the motion of the sphere is locked to the substrate and to the bulk, respectively. The sphere forms an inertial load in (**A**) and an elastic load in (**D**). In panels (**B**,**C**), the amplitude of motion of the sphere is large. In (**B**), the sphere rotates about the contact, in (**C**) it rotates about its center. Adapted from [171].

**Figure 36:**
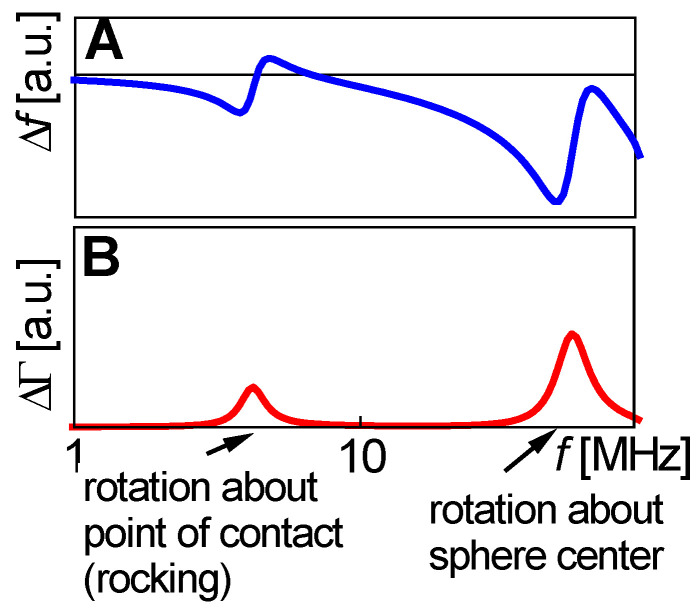
Shifts of frequency in panel (**A**) and bandwidth in panel (**B**) as computed with an FEM simulation for the configuration shown in Figure 35. There are two separate coupled resonances. These are the consequence of two dynamical variables, which are translation and rotation. Translation and rotation are always coupled, but there are two special linear combinations of translation and rotation, which both produce a resonance. Only the transverse force at the point of contact shifts Δ*f* and ΔΓ, a torque does not take an influence. The relative importance of the force compared to the torque is larger for the high-frequency mode. The sphere rotating about the point of contact mostly exerts a torque. This low-frequency mode therefore appears as less pronounced when probed with the QCM. Adapted from [171].

Figure 35 and Figure 36 illustrate the situation at hand of an FEM simulation. The simulation modeled a cylinder (rather than a sphere) because the simulation occurred in two dimensions. Also, the environment was a liquid. In agreement with Equation (85), two coupled resonances are found. For the higher frequency, the axis of rotation is close to the center of the sphere. For the lower frequency, the sphere rotates about the point of contact. Such a rotation is equivalent to a superposition of a rotation about the sphere center and a translation. 

Figure 36 shows Δ*f* and ΔΓ versus frequency as derived from the FEM calculation. Because this is a simulation, the frequency is not limited to the odd integers of the fundamental. One observes two coupled resonances, one stronger than the other. 

Figure 36 looks similar to an absorption spectrum form IR spectroscopy, which is no coincidence [171]. Molecules can vibrate in different ways, just like the sphere on the plate may combine translation and rotation in different ways. When probed with IR waves, some vibrational modes (like the carbonyl stretching mode) show prominently in the spectrum, while others are weaker. Some vibrations are not seen in IR light at all. These are “forbidden”, meaning that the vibration is not linked to an oscillating dipole. The vibrations of homonuclear diatomic molecules are forbidden. They are only seen in Raman spectroscopy. They are not seen in IR spectroscopy because the vibration “does not couple” to the electromagnetic wave. Some vibrations are “weakly allowed”. They appear to be not coupled to IR light, at first glance, but there is a loophole. The overtone vibrations in IR spectroscopy are forbidden in the frame of the harmonic resonator. The selection rule is Δ*v* = ±1 (with *v* the vibration quantum number). Anharmonicity weakens the selection rule. The overtones are seen, albeit with a small integrated line strength. 

The coupled resonance to the left in Figure 36 is weakly allowed in that sense. The vibration mostly is a rotation about the point of contact. The restoring force mostly amounts to a torque. Torques are not seen by the QCM. The QCM only sees transverse forces. If the axis of rotation would intersect the point of contact, exactly, this coupled resonance would be “forbidden”. The vibration would not couple to the substrate’s transverse motion. Looking at the mode a little closer (Figure 35B), one finds that the axis of rotation is slightly displaced from the contact. For that reason, the vibration does exert a small transverse force and is seen by the QCM, although with a small line strength. (Coupling across the liquid phase may also play a role.)

### 6.3. Other Types of Coupled Resonances

This text elaborates in some depth on coupled resonances caused by films and coupled resonances caused by particles. More generally, a “coupled resonance” can denote any load, which lets $\Delta \tilde{f}$ be described by Equation (79). Two other noteworthy examples are the following:

- Standing compressional waves (Section 8.1) give rise to coupled resonances, when the distance to the opposite cell wall is an integer multiple of half the wavelength. At these distances, the compressional wave is a standing wave and the damping is large. This phenomenon can be exploited to check for the magnitude of compressional wave effects. The experiment is simple. One lets the water level in an open cell slowly decrease by evaporation overnight. Figure 7 in [172] shows data of this kind. In this example, the compressional wave effects were much stronger on the fundamental than at 15 MHz. This is a general rule and one of the reasons, why data from the fundamental often are discarded from the analysis.
- The vibration of interest may couple to other modes of vibration of the crystal, where the exact mechanism of coupling is unclear and where even the nature of the other mode is unclear. These so-called “activity dips”, which sometimes occur when ramping temperature up or down, can be a problem in time and frequency control [173]. An activity dip lets the bandwidth increase at a certain temperature and lets the frequency go through a corresponding antisymmetric pattern. Activity dips are not discussed further here, even though they are occasionally seen in sensing, mostly during temperature sweeps.

## 7. Piezoelectric Stiffening

Piezoelectric stiffening is an advantage in time and frequency control. It is closer to a problem in sensing. Piezoelectric stiffening also is instructing from a fundamental point of view. 

When a bare piezoelectric plate is sheared, an electrical polarization is created in addition to the strain. The electrical energy contained in the polarization contributes to the overall energy of the sheared plate, thereby increasing the plate’s stiffness. When the crystal surfaces are plated with electrodes and when these electrodes are short-circuited, the piezoelectrically induced polarization is compensated by a corresponding charge in the electrodes. Shearing the crystal then becomes easier because no electrical work is done. The spring constant decreases and so does the resonance frequency because of $\omega_{0}\approx {\left(\kappa_{R}{/m}_{R}\right)}^{1/2}.\;$ Situations between those two limits are created by connecting the electrodes across a capacitor or some other electrical impedance.

The situation is reminiscent of the difference between the two heat capacities of a gas, *C_p_* and *C_V_*. *C_p_* and *C_V_* are determined at constant pressure and constant volume, respectively. *C_p_* is larger than *C_V_* because the volume expansion, which occurs when pressure is kept constant, amounts to a mechanical work. The open-circuit stiffness of the piezoelectric plate and the stiffness with short-circuited electrodes correspond to boundary conditions of constant charge (total charge in the bulk and at the surface) and of constant electrical potential, respectively. If the voltage between the two electrodes is zero, the creation of charge at the surfaces is not linked to an electrical work.

One can include a voltage-tunable capacitor (a varicap diode) into the circuitry and tune the resonator’s frequency this way. This device is the voltage-controlled crystal oscillator (VCXO [52] or VCO). An approximative relation connects the frequency shift to the external capacitance, $C_{\mathrm{ext}}$, as:

(87) $$\frac{{\Delta f}_{\mathrm{PE}}}{f}-\;\frac{{\Delta f}_{\mathrm{PE},\infty }}{f}\;=\;\frac{1}{2}\frac{C_{1}}{C_{0}{\;+\;C}_{\mathrm{ext}}}\;$$

$C_{0}$ is the resonator’s electrical capacitance (the “parallel capacitance”) and $C_{1}$ is the motional capacitance (Section 8.4). The subscript *PE* stands for piezoelectric stiffening, ∞ stands for infinite $C_{\mathrm{ext}}$. An infinite external capacitance amounts to short-circuit electrodes (constant potential). Note that $C_{\mathrm{ext}}=\infty$ is not a practical reference state because the current is then entirely absorbed in $C_{\mathrm{ext}}$ and does not cause a vibration of the crystal. 

$C_{0}$ and $C_{1}$ are related as:

(88) $$\frac{C_{1}}{C_{0}}=\frac{8}{{\left(n\pi \right)}^{2}}k_{t,\mathrm{eff}}^{2}\;\;\;\mathrm{with}\;\;k_{t,\mathrm{ideal}}^{2}=\frac{{\;e}_{26}}{\varepsilon_{q}\varepsilon_{0}G_{q}}\;$$

$k_{t}$ is the electromechanical coupling coefficient, $e_{26}$ = 9.65 × 10^−2^ C/m^2^ is the piezoelectric stress coefficient, $\varepsilon_{q}$ = 4.54 is the dielectric constant, and $G_{q}$ = 29 × 10^9^ Pa is the shear modulus. For AT-cut quartz, $k_{t}^{2}$ is about 0.8%. Piezoelectric coupling is not particularly strong for quartz. The tuning range of quartz resonators is smaller than the tuning range of resonators made from competing materials such as langasite [174]. Equation (88) distinguishes between an “ideal” coupling coefficient (derived from the material constants) and an “effective” coupling coefficient (derived $C_{0}$ and $C_{1}$). The two are different because the geometry (electrode shape, energy trapping, …) takes an influence. Inserting numbers into Equation (87), one finds the pulling range to be around 1 kHz. 

In a sensing context, all kinds of stray capacitances (more generally, all kinds of electrical boundary conditions) enter $C_{\mathrm{ext}}$. For sensing, piezoelectric stiffening mostly is an annoyance. In particular, the cables must not move while the experiment is running. If they do, this will affect the capacitance seen by the crystal. A second problem are electric fields permeating the sample from the surface of the resonator. One avoids that by making the front electrode larger than the back electrode and by grounding the front electrode well. This problem also affects electrode-less resonators [175].

For the sake of formal consistency, we formulate a modified version of Equation (88), which lets the sample’s electrical impedance look like any other load entering the small-load approximation. This equation is:

(89) $$\frac{\Delta {\tilde{f}}_{\mathrm{PE}}}{f_{0}}=\frac{i}{{\pi Z}_{q}}\left[\frac{{4e}_{26}^{2}A_{\mathrm{eff}}}{d_{q}^{2}}\right]\left({\left({i\omega C}_{0}+{\tilde{Z}}_{\mathrm{ext}}^{-1}\right)}^{-1}-{\left({i\omega C}_{0}+{\tilde{Z}}_{\mathrm{ext},\mathrm{ref}}^{-1}\right)}^{-1}\right)$$

where ${\tilde{Z}}_{\mathrm{ext}}$ is the sample’s electrical impedance. The term in square brackets converts between an electrical and an acoustic impedance (Section 8.4). The hypothetical reference state with short-circuited electrodes was replaced by a more realistic reference state with some external electrical impedance, ${\tilde{Z}}_{\mathrm{ext},\mathrm{ref}}$. For more details see Chapter 5 in [5]. 

In principle, piezoelectric stiffening might provide for a scheme to measure the sample’s electrical impedance in addition to its shear-wave impedance. That has turned out to be difficult [176,177]. In practice, one will usually determine electrical impedances with electrical equipment (as in electrochemical impedance spectroscopy, EIS, or, more generally, electrical impedance spectroscopy).

## 8. Beyond the Parallel-Plate Model

### 8.1. Energy Trapping, Compressional Waves

**Figure 37:**
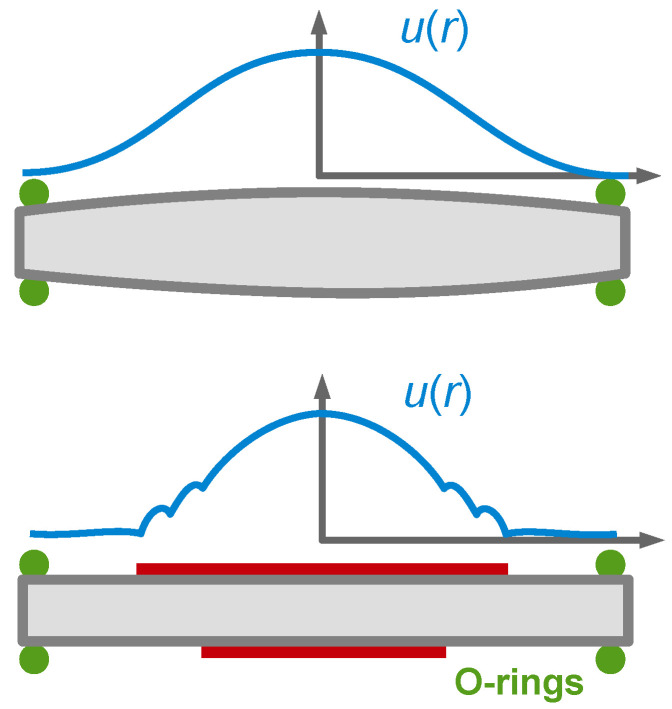
Energy trapping allows to mount resonators at the edge with little damping. When the resonator is thicker in the center than at the edge, the shear vibration is confined to the center. Ideally, the amplitude distribution is smooth (**top**). Experiments often evidence small-scale variability (**bottom**, [172,186]). $u^{2}\left(r\right)$ is the weight function to be applied in area averaging following Equation (26).

The limitations of the laterally infinite parallel plate as a model for the QCM come in two forms. Firstly, the edges take an influence on the mode of vibration for AT-cut quartz. Secondly and more importantly, the practical resonators do not actually have parallel surfaces, at least in acoustic terms (Figure 37). In order to mount the resonator between O-rings with little damping, the resonator’s vibration amplitude at the edge must be as small as possible. That is achieved by making the resonator slightly thicker in the center than at the edge [178,179]. The mechanism is called energy trapping. The resonator may be viewed as an acoustic lens. The concave surfaces focus the acoustic energy to the center. The situation has been analyzed with analytical theory in considerable depth [180,181].

Resonators designed for use on the fundamental indeed have concave surfaces. The resonators used in sensing mostly achieve energy trapping with keyhole-shaped electrodes. Often the back electrode is smaller and thicker than the front electrode, so that the back electrode defines the amplitude distribution. If convex surfaces are employed, the amplitude distribution is similar to a Gaussian (circular or elliptical) [182]. With key-hole electrodes, there is small-scale variability (bottom in Figure 37). The displacement pattern can be visualized in a few different ways, none of them being simple [172,183,184,185]. These images reveal rather complex patterns with a considerable amount of irregularity [186,187]. In particular, the patterns may deviate from what symmetry would dictate in the absence of crystal defects.

Energy trapping has two important consequences, which are an increased resonance frequency and flexural contributions to the mode of vibration. 

Why would energy trapping increase the frequency? The apparent stiffness of a vibrating body depends not only on the material’s elastic modulus but also on the steepness of the gradients in displacement. For instance, the effective spring constant of the parallel plate is given as:

(90) $$\kappa_{R}\;=\;\frac{A_{\mathrm{eff}}G_{q}}{d_{q}}\frac{{\left(n\pi \right)}^{2}}{2}\;=\;\frac{{\left(n\pi \right)}^{2}}{2}\kappa_{q,\mathrm{stat}}$$

High overtones have a large effective spring constant because the shear gradients are strong. The more deformation is contained in a mode, the higher is the energy in the elastic deformation. 

**Figure 38:**
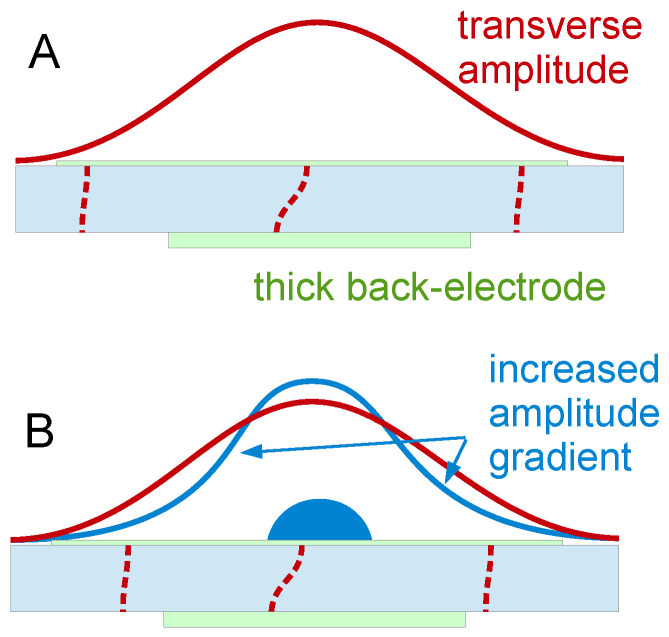
Application of a load to the center of the plate, only (as shown in panel (**B**)), improves energy trapping, thereby increasing the resonance frequency with respect to the unloaded resonator in panel (shown in (**A**)).

Increased energy trapping increases the steepness of the in-plane gradients, thereby increasing frequency. This effect is noticed when comparing the resonance frequencies of the different overtones. These do not occur at the exact integer multiples of the fundamental because of energy trapping (and, also, because of piezoelectric stiffening, but the latter influence is smaller) [188]. The influence, which energy trapping takes on the resonance frequency, is problematic when a sample contacts the resonator at the center, only, because the sample then improves the energy trapping, thereby increasing frequency (Figure 38). This effect is superimposed onto the sample’s load impedance (see the discussion below Equation (34)).

**Figure 39:**
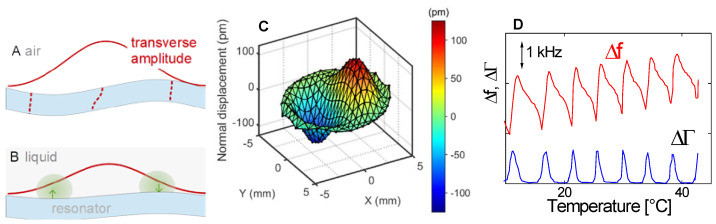
(**A**,**B**) When a parallel plate experiences a hypothetical pure thickness-shear deformation with in-plane gradients, volume is not conserved at those places, where the in-plane gradient is large. The pure thickness-shear mode therefore is not realized: The plate bends. In liquids, the bending is reduced because the liquid is compressed as well. (**C**) A map of the vertical displacement as determined with laser doppler velocimetry (LDV). Adapted from [172]. (**D**) Δ*f* and ΔΓ determined in a temperature ramp. The opposite wall of the liquid cell was parallel to the resonator surface, which generated standing compressional waves with λ depending on temperature. Adapted from [189].

A second consequence of energy trapping are flexural contributions to the displacement pattern. Because the amplitude of shear varies between the center and the edge, the resonator bends (Figure 39A–C). On the high overtones, bending is reduced for the reasons discussed in [172]. Because bending is particularly strong on the fundamental, data from the fundamental often are discarded from the analysis. Bending is reduced in a liquid environment because the liquid itself is compressed in the regions with in-plane gradients of the transverse displacement. It exerts a pressure onto the plate, reducing the flexural displacement (green in Figure 39B). 

Flexural motion launches compressional waves and standing compressional waves can cause coupled resonances. Figure 39D shows an example (from [189], see also [190,191]). The crystal and the wall of the liquid cell opposite to the crystal form a cavity for compressional ultrasound. The cavity resonates when the distance between the two surfaces is equal to *n*λ_comp_/2 with *n* an integer. The wavelength and the dimensions of the cell vary with temperature, which causes the nightmare shown in Figure 39D. In order to not let that happen, one may design the cell such that the resonator surface is inclined relative to the opposite wall. That does not remove the compressional waves, it only avoids the coupled resonances. This recommendation was actually formulated as early as 1987 by Eggers and Funk in the same paper, in which they propose to analyze QCM data in terms of the shift of the complex resonance frequency [33]. Eggers and Funk placed a “spoiler” (an irregular piece of Teflon) in front of the crystal in order to deflect the compressional waves. When working with open cells, one may place a paper clip onto the air-water interface (held there by surface tension). Few researchers have reported on temperature sweeps employing liquid cells. It is always difficult to say why certain experiments were not done or not reported, but one may guess that compressional waves were a problem. 

Effects of compressional-waves can never be eliminated in measurements of viscosity because one compares frequencies measured in air to frequencies measured in liquid. With regard to adsorption experiments in liquid, one can hope that the effects of compressional waves remain constant during experiment and therefore disappear from the frequency shift. 

A historical note: Soft matter was studied with acoustic resonators in the 1930s to 1950s, already. The frequencies mostly were in the kHz range. Torsional resonators and reflection devices [75] were used, mostly. Thickness-shear resonators were available at that time as clocks. Mason and McSkimin did use these when they expanded their frequency range to beyond 1 MHz, but they used them in an indirect way. In 1949 they glued AT-cut crystals to the ends of cylindrical rods of fused quartz. The crystals were excited by a radio pulse, thereby launching a transverse acoustic wave. The wave travelled down the cylinder, was reflected at the other end (at the interface to the liquid under study, see the remarks around Equation (37)) and returned to the crystal, which transduced the reflected wave’s shear stress to a voltage. The liquid’s viscosity was inferred from the reflected amplitude. In the introduction, the authors discuss using the plates as such: *“Consideration was given to the use of a thickness vibrating shear crystal of the AT or BT type, but it was found that the shear motion was too closely coupled to other modes of motion, such as flexure modes, to give reliable results. Hence another method had to be used.”* In hindsight, these problems turned out to be less severe than the authors had believed.

With some diligence, crystals not employing energy trapping can be mounted such that they are only weakly damped by the O-rings. Such crystals have electrodes covering the entire area rather than key-hole electrodes. However, such resonators immersed in liquids still display compressional-wave effects. This is evidenced with open cells, the water level of which slowly decreases due to evaporation [172]. Coupled resonances as shown in Figure 39D are still seen. Evidently, the edges of the resonator alone give rise flexure modes because of the anisotropy of the elastic constants.

Experiments in the dry can occur even with no electrodes at all. An electrodeless resonator blank can be placed on a rough metal surface. The other electrode can be placed above the resonator, leaving an air gap. The rough surface supports the blank across small asperities, which hardly dampen the resonance [81]. The problem with this arrangement is that the resonance frequency depends on the width of the air gap. The upper electrode must be mounted rigidly. Once this problem is solved, the experiment is rather clean and well-defined.

### 8.2. Anharmonic Sidebands

**Figure 40:**
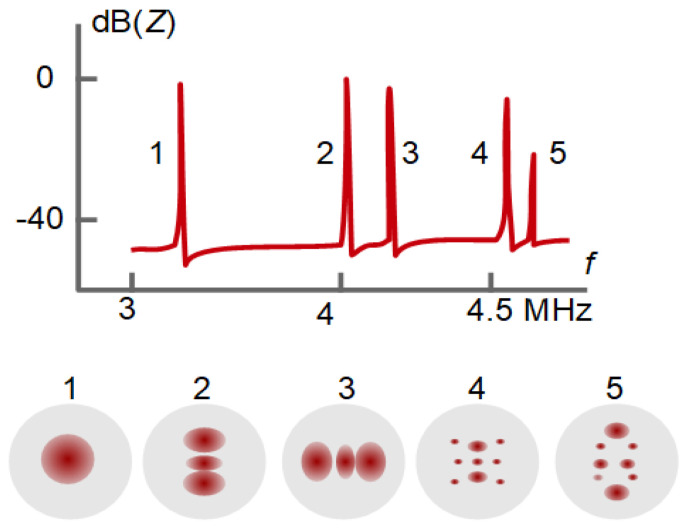
Vibrational patterns of the 1-00-mode (“1”) and a few anharmonic sidebands. The top shows $\left|{\tilde{Z}}_{\mathrm{el}}\right|$ versus frequency. The mode shape was determined with a variant of optical reflectometry. Adapted from [183].

Anharmonic sidebands are solutions to the acoustic boundary value problem, which contain nodal planes perpendicular to the surface. Figure 40 shows examples. The maps of the displacement amplitude were produced by Sauerbrey, based on a somewhat intricate optical method [192]. Anharmonic sidebands have been exploited for sensing [193], but the examples are scarce. The important modes for sensing do not have nodal planes perpendicular to the plate surface. They might be called *n*-00-modes, where *n* is the number of nodal planes parallel to the surface and the two other indices count the number of radial and elliptical nodal planes. 

**Figure 41:**
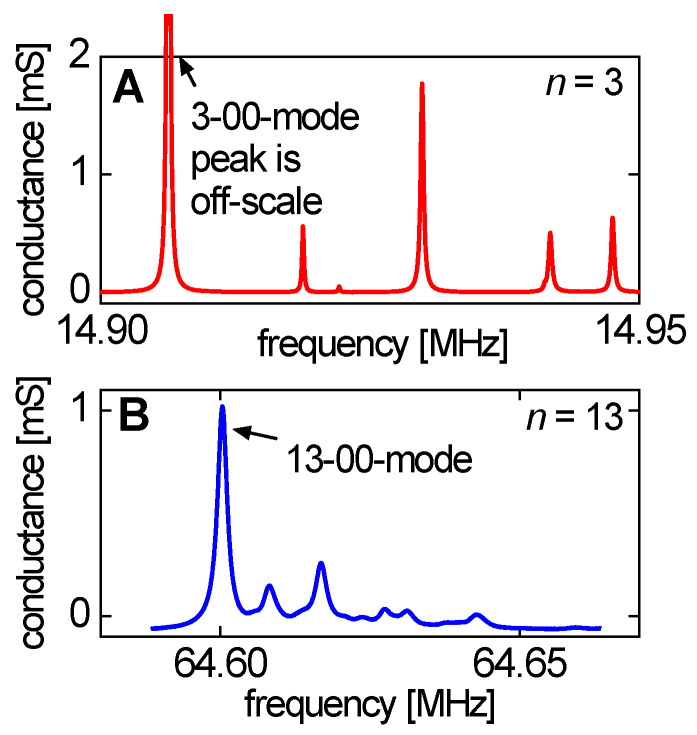
If the anharmonic sidebands are not well separated from the *n*-00-mode, there will be coupling. The frequency and the bandwidth of the *n*-00-mode then vary unsystematically in experiment. In (**A**), the anharmonic side bands *are* well separated from the 3-00-mode. The 13-00-mode shown in (**B**) is of little use for sensing.

Anharmonic sidebands are a problem if they overlap with the *n*-00-modes. They then couple to these modes with detrimental consequences. By and large, the 13-00-mode, shown in Figure 41 is useless for sensing. A resonance curve can certainly be fitted to the admittance trace, but Δ*f* and ΔΓ as derived from these fits are bound to vary erratically in experiment. Overlap with anharmonic sidebands is much less of a problem in air than in liquid because of the smaller bandwidth. In air, overtone orders up to 19 can be evaluated.

### 8.3. Towards 3D-Modelling: The Small-Load Approximation in Tensor Form

The following section borrows from quantum mechanics. In quantum mechanics, a small perturbation (for instance caused by a magnetic field or by neighboring molecules) is sometimes superimposed onto a stronger, unperturbed Hamiltonian. If the perturbation is small, the Schrödinger equation does not have to be solved again from scratch. One starts from the solution to the unperturbed Schrödinger equation and computes small corrections to the orbitals and energies. Importantly, the 1st-order shift in energy does not require knowledge of the 1st-order shifts of the corresponding orbital. It can be computed from the unperturbed orbital.

Let the total Hamiltonian consist of an unperturbed operator, ${\underline{H}}_{0}$, and a small perturbation, ${\underline{H}}_{1}$:

(91) $$\underline{H}={\underline{H}}_{0}+{\underline{H}}_{1}$$

Let the solution to the unperturbed Hamiltonian, $\psi^{\left(0\right)}$, be unique (no degeneracy). Perturbation theory predicts the 1st-order correction of the energy eigenvalue, *E*^(1)^, as

(92) $$E^{\left(1\right)}=\frac{\int_{\mathrm{Volume}\;}\psi^{(0)*}{\underline{H}}_{1}\psi^{\left(0\right)}dV}{\int_{\mathrm{Volume}\;}\psi^{(0)*}\psi^{\left(0\right)}dV}$$

The superscript (0) denotes the solution to the unperturbed Hamiltonian. “∗” denotes complex conjugation. If the wave function is normalized, the denominator is unity (and is not actually needed). It was included for the sake of generality. (The 0th-order solution will be non-normalized further down.) Again, the 1st-order correction to the energy can be computed from the *un*perturbed wave function.

In the acoustics problem, the Hamilton operator is replaced by the $\omega^{2}$-operator, $\underline{\omega^{2}}$. Standing elastic waves in the unloaded crystal follow the relation:

(93) $${\left(\omega^{2}\right)}^{\left(0\right)}{\hat{u}}_{i}^{\left(0\right)}\;=\;\underline{\omega^{2}}\;{\hat{u}}_{i}^{\left(0\right)}\;=\;\frac{1}{\rho_{q}}\underset{j}{\sum }\frac{{d\hat{\sigma }}_{ij}^{\left(0\right)}}{{dr}_{j}}\;=\;\frac{1}{\rho_{q}}\underset{jkl}{\sum }\frac{d}{{dr}_{j}}\left(c_{ijkl}\frac{1}{2}\left(\frac{d{\hat{u}}_{l}^{\left(0\right)}}{{dr}_{k}}\;+\;\frac{d{\hat{u}}_{k}^{\left(0\right)}}{{dr}_{l}}\right)\right)\;$$

The indices *i*, *j*, *k*, and *l* run over *x*, *y*, and *z*. As before, superscript (0) denotes the solution to the unperturbed problem. Equation (93) is an eigensystem, similar to the time-independent Schrödinger equation. The wave function is replaced by the displacement field, $\hat{u}$ (a vector, rather than a scalar). ${\hat{\sigma }}_{ij}^{\left(0\right)}{/dr}_{j}$ is the force density. ${\hat{\sigma }}_{ij}$ is the stress tensor, given as:

(94) $${\hat{\sigma }}_{ij}\;=\;\underset{kl}{\sum }c_{ijkl}{\hat{\epsilon }}_{kl}\;=\;\underset{kl}{\sum }c_{ijkl}\frac{1}{2}\left(\frac{d{\hat{u}}_{l}}{{dr}_{k}}\;+\;\frac{d{\hat{u}}_{k}}{{dr}_{l}}\right)\;$$

where ${\hat{\epsilon }}_{kl}$ is the deformation tensor and *c_ijkl_* is the stiffness tensor. Because Equation (94) holds generally, the superscript (0) was omitted. 

The stiffness tensor of the unperturbed problem must be real (no internal friction) because perturbation theory requires the unperturbed operator to be self-adjoint. An unperturbed operator including dissipation would violate self-adjointness. Self-adjointness is not required, however, for the perturbation operator. Internal dissipation can therefore be included in the formalism as a perturbation. This perturbation is separate from the perturbation by the sample (Equation (95)). Because the unperturbed operator is real, the eigenvalue to the unperturbed problem, ${-\left(\omega^{2}\right)}^{\left(0\right)}$, and the 0th-order displacement field, ${\hat{u}}^{\left(0\right)}$, are real, as well. A similar situation was encountered below Equation (21), when $Z_{q}$, $c_{q}$, and $f_{\mathrm{ref}}$ were expressed as real parameters.

When a sample exerts a periodic stress onto the surface, this stress amounts to a perturbation. The eigensystem with surface traction included is:

(95) $${\left(-\tilde{\omega^{2}}\right)}_{\mathrm{tot}}{\hat{u}}_{i}\;=\;\left({\left(\underline{\omega^{2}}\right)}_{0}+{\left(\underline{\omega^{2}}\right)}_{1}\right){\hat{u}}_{i}\;=\frac{1}{\rho_{q}}\underset{jkl}{\sum }\left(\frac{d}{{dr}_{j}}\left(c_{ijkl}\frac{1}{2}\left(\frac{d{\hat{u}}_{l}}{{dr}_{k}}\;+\;\frac{d{\hat{u}}_{k}}{{dr}_{l}}\right)\right){\;+\;n}_{j}{\tilde{Z}}_{L,ijk}i\omega {\hat{u}}_{k}\delta \left(r-S\right)\right)$$

The i in iω is the imaginary unit (not an index). δ(r−S) is the Dirac δ-function, where **S** is a position on the resonator surface. *n_j_* are the components of the surface normal. The eigenvalue, ${\left(-\tilde{\omega^{2}}\right)}_{\mathrm{tot}}$, is complex because ${\tilde{Z}}_{L,ijk}$ is complex. ${\tilde{Z}}_{L,ijk}$ is the load impedance in tensor form, defined by:

(96) $$-{\hat{\sigma }}_{S,ij}\;=\;\underset{k}{\sum }{\tilde{Z}}_{L,ijk}{\hat{v}}_{S,k}\;=\;\underset{k}{\sum }{\tilde{Z}}_{L,ijk}i\omega {\hat{u}}_{S,k}\;.$$

As in Equation (20), there is a minus sign in front of the stress because the stress is exerted into the direction of –*z*. 

The displacement field in Equation (95), $\hat{u}\neq {\hat{u}}^{\left(0\right)}$, is the solution to the *total* $\omega^{2}$-operator. One might write $\hat{u}\approx {\hat{u}}^{\left(0\right)}+{\hat{u}}^{\left(1\right)}$ with ${\hat{u}}^{\left(1\right)}$ the 1st-order correction, but the 1st-order correction to the displacement field does not concern us any further. We are only interested in the shift of the eigenvalue. The eigenvalue is approximated as the sum of a 0th-order and a 1st-order term:

(97) $${\left(-\tilde{\omega^{2}}\right)}_{\mathrm{tot}}\;\approx \;{\left(-\omega^{2}\right)}^{\left(0\right)}\;+\;{\left(-\tilde{\omega^{2}}\right)}^{\left(1\right)}\;.$$

Following perturbation theory, the 1st-order term can be computed from the 0th-order displacement field ${\hat{u}}^{\left(0\right)}$ as:

(98) $$\begin{array}{ll} {\left(\tilde{\omega^{2}}\right)}^{\left(1\right)} & \approx \;\frac{i\omega }{\rho_{q}}\frac{\sum_{ijk}\int_{\mathrm{Volume}}{\hat{u}}_{S,i}^{\left(0\right)}n_{j}{\tilde{Z}}_{L,ijk}{\hat{u}}_{S,k}^{\left(0\right)}\delta \left(r-S\right)d^{3}r}{\sum_{i}\int_{\mathrm{Volume}}{\hat{u}}_{i}^{\left(0\right)}{\hat{u}}_{i}^{\left(0\right)}d^{3}r}\\ & =\frac{i\omega }{\rho_{q}}\frac{\sum_{ijk}\int_{\mathrm{Surface}}{\hat{u}}_{S,i}^{\left(0\right)}n_{j}{\tilde{Z}}_{L,ijk}{\hat{u}}_{S,k}^{\left(0\right)}d^{2}r_{S}}{\sum_{i}\int_{\mathrm{Volume}}{\hat{u}}_{i}^{\left(0\right)}{\hat{u}}_{i}^{\left(0\right)}d^{3}r}\\ & =\frac{i\omega }{\rho_{q}}\frac{\int_{\mathrm{Surface}}\left({\hat{u}}_{S}^{\left(0\right)}⊗n\right)\;:\;{\tilde{Z}}_{L}\cdot {\hat{u}}_{S}^{\left(0\right)}d^{2}r_{S}}{\int_{\mathrm{Volume}}{\hat{u}}^{\left(0\right)}\cdot {\hat{u}}^{\left(0\right)}d^{3}r}\; \end{array}$$

The denominator is needed because ${\hat{u}}^{\left(0\right)}$ is not normalized. (It is not even dimensionless.) In the numerator, the volume integral has turned into a surface integral because of the δ-function. Complex conjugation as in Equation (92) is not needed because ${\hat{u}}^{\left(0\right)}$ is real. In line 3, the center-dot is the vector product (contraction over one index, “dot product”). “:” denotes contraction over two indices and “⊗” is the outer product. 

This concludes the perturbation calculation. We are left with the task to compute $\Delta \tilde{f}\;$ from ${\left(\tilde{\omega^{2}}\right)}^{\left(1\right)}$ and ${\left(\omega^{2}\right)}^{\left(0\right)}$. This calculation proceeds as:

(99) $$2\pi \Delta \tilde{f}\;=\;\sqrt{{\left(\omega^{2}\right)}^{\left(0\right)}\;+\;{\left(\tilde{\omega^{2}}\right)}^{\left(1\right)}}-\omega^{\left(0\right)}\;=\omega^{\left(0\right)}\left(\sqrt{1\;+\;\frac{{\left(\tilde{\omega^{2}}\right)}^{\left(1\right)}}{{\left(\omega^{2}\right)}^{\left(0\right)}}}\;-\;1\right)\;\approx {\;\omega }^{\left(0\right)}\left(1\;+\;\frac{1}{2}\frac{{\left(\tilde{\omega^{2}}\right)}^{\left(1\right)}}{{\left(\omega^{2}\right)}^{\left(0\right)}}\;-\;1\right)\;=\;\frac{1}{2}\frac{{\left(\tilde{\omega^{2}}\right)}^{\left(1\right)}}{\omega^{\left(0\right)}}\;\;$$

Taylor expansion ((1 + ε)^1/2^ ≈ 1 + ε/2 for ε ≪ 1) was applied. Combining Equation (99) with Equation (98) and using ω ≈ $\omega^{\left(0\right)}$ leads to:

(100) $$\Delta f\;+\;i\Delta \Gamma \;\approx \;\frac{i}{{4\pi \rho }_{q}}\frac{\sum_{ijk}\int_{\mathrm{Surface}}{\hat{u}}_{S,i}^{\left(0\right)}n_{j}{\tilde{Z}}_{L,ijk}{\hat{u}}_{S,k}^{\left(0\right)}d^{2}r_{S}}{\sum_{i}\int_{\mathrm{Volume}}{\hat{u}}_{i}^{\left(0\right)}{\hat{u}}_{i}^{\left(0\right)}d^{3}r}\;$$

This is the small-load approximation in tensor form. It was first written down in slightly different form by Pechhold in [194].

We briefly convince ourselves that Equation (100) reduces to Equation (25) for the parallel plate. For the parallel plate, the displacement occurs along *x* and is of the form ${\hat{u}}_{S}\cos\left(k_{q}z\right)$. The integral in the denominator turns into ${\hat{u}}_{S}^{2}A_{\mathrm{eff}}d_{q}/2$. The only nonzero component of ${\tilde{Z}}_{L,ijk}$ is ${\tilde{Z}}_{L,xzx}$, called ${\tilde{Z}}_{L}$ in Equation (25). The surface normal is along *z*. The displacement in the numerator is ${\hat{u}}_{S}$. For the parallel plate, Equation (100) turns into:

(101) $$\Delta f\;+\;i\Delta \Gamma \;\approx \;\frac{i}{{4\pi \rho }_{q}}\frac{{\hat{u}}_{S}^{2}A_{\mathrm{eff}}\langle {\tilde{Z}}_{L}\rangle }{{\hat{u}}_{S}^{2}A_{\mathrm{eff}}d_{q}/2}\;\;$$

With $\rho_{q}d_{q}{\;=\;m}_{q}{\;=\;Z}_{q}/\left({2f}_{0}\right)$ this relation is equivalent to Equation (25):

Limitations of the formalism are:

- Piezoelectric stiffening is not included. That can be done (in tensor form). It is simply a matter of not letting oneself be intimidated by large equation systems.
- Some perturbations may actually be large perturbations. Among these are the compressional waves, because the plate’s stiffness under bending (not shear) may be too small to let the normal pressure exerted by compressional waves be a small perturbation [127].
- The above mathematics covers the 1st-order perturbation, only. 3rd-order perturbation is sometimes needed (Box 2).
- Calculating the vibration pattern of the unloaded crystal with electrodes is a challenge. If such a calculation is not feasible, the mode of vibration can still be determined experimentally with laser Doppler vibrometry (LDV, Figure 39C).

Among the benefits linked to Equation (100) are:

- Equation (100) is general. It also applies to other resonators (such as torsional resonators [60,61,65] or nanoresonators [195]).
- It clarifies, why the statistical weight in area averaging in Equation (26) is the square of the local amplitude of oscillation.
- It explains why the Sauerbrey relation is slightly incorrect on the low overtones, even for rigid films in dry environments. The problem is linked to the modal mass (Section 8.6).
- It allows to quantitatively estimate the effects of increased energy trapping discussed around Figure 38. In particular, it explains why the freezing of a liquid drop on the resonator surface (water, hexadecane, …) lets the resonance frequency increase [196].

The limitations acknowledged: Equation (100) is the starting point for full-fledged 3D-modelling. 

### 8.4. The 4-Element Circuit and the Electromechanical Analogy 

An equivalent circuit (also “lumped-element circuit”) contains discrete elements, networked together in some way. This involves two separate approximations. Firstly, the real-world device may or may not consist of discrete elements. Printed circuit boards (PCBs) often contain discrete elements, linked by conductive tracks with small resistance. Even then, stray capacitances (often needed for a faithful representation with an equivalent circuit) have no corresponding elements on the PCB. In real-word acoustics and mechanics, discrete elements are rare. Two masses linked together with a spring are an example (Figure 1B). The bell is a counter example (Figure 1C). A second approximation concerns discrete elements in the real world, which are represented with two impedances in the equivalent circuit. A real-world inductor has some ohmic resistance and is therefore depicted as an inductor in series with an ohmic resistor. In mechanics, a contact often dissipates energy and is therefore depicted as a spring in parallel to a dashpot (Figure 31).

**Figure 42:**
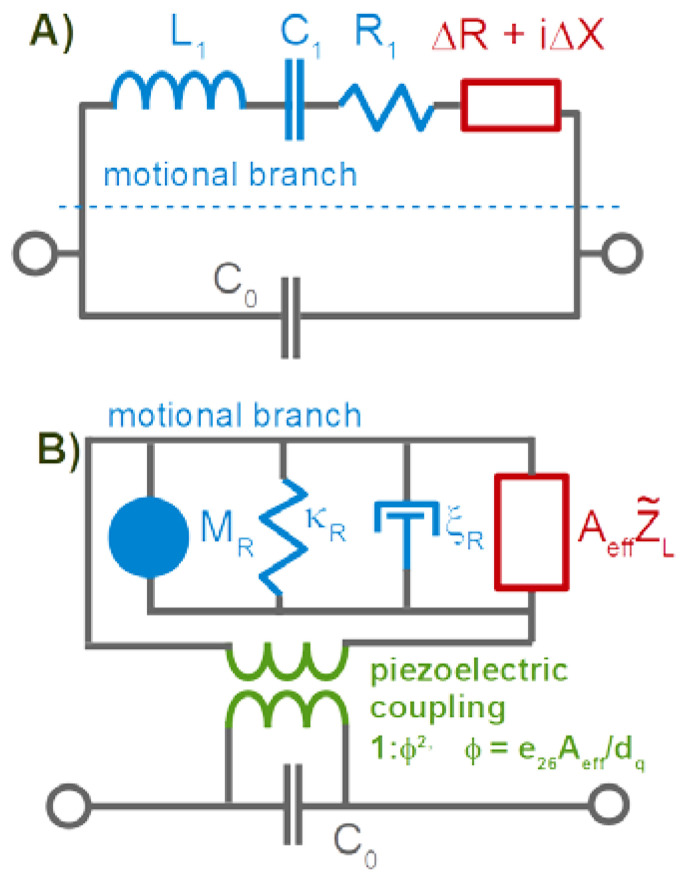
(**A**) The Butterworth-van-Dyke equivalent circuit (also: “4-element circuit”). This version of the 4-element circuit actually contains 5 elements because the load (Δ*R* + iΔ*X*) was included. (**B**) A version of the circuit, where the motional branch is drawn with mechanical elements.

Figure 42A shows an electrical equivalent circuit of a quartz resonator (or some other piezoelectrically driven resonator). When drawn without the load (in red on the right-hand side), this is the Butterworth-van-Dyke circuit (BvD circuit, also: “4-element circuit”). The upper branch (the motional branch) contains an inductance, a capacitance, and a resistance. This circuit exploits the electromechanical analogy, which maps the mass, the spring, and the dashpot onto the inductance, the capacitance, and the resistance. The motional branch can be modeled with electrical impedances because piezoelectricity acts as an impedance converter. While the discrete impedances certainly are an idealization, Figure 42A reproduces the resonator’s overall impedance close to the resonances well. Note that the values of $L_{1}$, $R_{1}$, and $C_{1}$ differ between overtones. 

There is a more general circuit (the Mason circuit [92]), which covers the entire frequency range (on-resonance and off-resonance, all overtones, parallel-plate model). In the Mason circuit, the impedances of the elements are not just inductances, resistances, or capacitances. For instance, one of them is i$A_{\mathrm{eff}}Z_{q}\tan\left({\tilde{k}}_{q}d_{q}/2\right)$. The Mason circuit also covers piezoelectric stiffening. (The 4-element circuit from Figure 42A does not.) Models of the QCM loaded with planar films can entirely be based on the Mason circuit [54,197]. The Mason circuit is interesting in a few ways, but working one’s way from the diagram to the frequency shift is laborious [198].

The capacitance at the bottom in Figure 42 is a genuinely electrical element. It is the “parallel capacitance”, formed by the electrodes on both sides of the plate. $C_{0}$ is larger than $C_{1}$ by about a factor of 1000 (Equation (88)). When the (electrical) Kirchhoff rules are applied to Figure 42A, an electrical admittance as shown in Figure 3 results.

It is instructive to also draw the corresponding mechanical circuit. A mechanical circuit of that kind was already shown in the introduction (Figure 1). The mechanical circuit contains a spring, a mass, and a dashpot. These mechanical impedances (such as iω$m_{R}$ for the mass and $\kappa_{R}$/(iω) for the spring) have dimensions of force to velocity. The acoustic impedances, on the other hand, are ratios of stress to velocity. Within the parallel-plate model, one converts between the two by multiplication with the area, $A_{\mathrm{eff}}$. (The 4-element circuit is not necessarily outside the parallel-plate model. It is general.)

Figure 42B makes the mechanical nature of the elements in the upper branch explicit. Importantly, the elements must be arranged in parallel (see Box 3). Following the mechanical Kirchhoff rules, impedances are additive, when the elements are arranged in parallel, while inverse impedances are additive, when the elements are arranged in series. 

**Figure 43:**
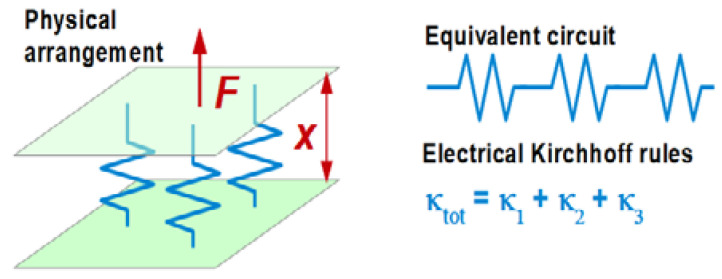
Electrical engineers sometimes draw mechanical equivalent circuits, such that the *electrical* Kirchhoff rules apply [52]. This is *not* the convention followed in Figure 42.

A side remark: Not everyone draws mechanical elements as in Figure 42B. If drawn as in Figure 43 on the right-hand side, the electrical Kirchhoff rules apply. The difficulty here is to always remember that the elements in this circuit are arranged differently from the elements in the real world. That would not problem in the context of Figure 42B because the discrete elements do not exist in the real world. It would be a problem in Figure 31.

The values of the circuit elements in Figure 42B are:

(102) $$m_{R}\;=\;\frac{A_{\mathrm{eff}}m_{q}}{2}{,\;\kappa }_{R}\;=\;\frac{A_{\mathrm{eff}}G_{q}}{d_{q}}\frac{{\left(n\pi \right)}^{2}}{2}{,\;\xi }_{R}\;=\;\sqrt{\kappa_{R}m_{R}}{\tan\delta }_{q}\;$$

Within the parallel-plate model, $\delta_{q}$ is the plate’s loss angle, ${G_{q}}^{\prime \prime }/{G_{q}}^{\prime }$. For practical resonators, the energy dissipated in the electrodes also contributes to $\xi_{R}$. 

Figure 42B represents piezoelectric coupling as a transformer. A key parameter of a transformer is the turns ratio, ϕ = $n_{2}$/$n_{1}$. $n_{1}$ and $n_{2}$ are the numbers of turns. Voltage, *U*, and current, *I*, are transformed as:

(103) $$U_{2}\;=\;\frac{n_{2}}{n_{1}}U_{1},\;\;\;\;\;\;I_{2}\;=\;\frac{n_{1}}{n_{2}}I_{1}$$

Following Equation (103), an impedance on one side of the transformer takes a different value when seen from the other side. The converted impedance is:

(104) $$Z_{2}\;=\;\frac{n_{2}^{2}}{n_{1}^{2}}Z_{1}\;=\;\phi^{2}Z_{1}\;$$

The transformer in Figure 42B is an impedance converter in this sense. It converts between an electrical and a mechanical impedance (${\tilde{Z}}_{\mathrm{mech}}=\phi^{2}{\tilde{Z}}_{\mathrm{el}}$, with $\phi {=e}_{26}A_{\mathrm{eff}}/d_{q}$). 

The following equations derive the small-load approximation from Figure 42B. In the absence of a load, the resonance condition is

(105) $${i\omega m}_{R}\;+\;\frac{\kappa_{R}}{i\omega }{\;+\;\xi }_{R}\;=\;0\;$$

Neglecting the dashpot, the resonance frequency is

(106) $$\omega_{0}\;=\;\sqrt{\frac{\kappa_{R}}{m_{R}}}\;=\;\sqrt{\frac{A_{\mathrm{eff}}G_{q}{\left(n\pi \right)}^{2}/\left({2d}_{q}\right)}{\left(A_{\mathrm{eff}}m_{q}\right)/2}}\;=\;\frac{n\pi }{d_{q}}\sqrt{\frac{G_{q}}{\rho_{q}}}\;=\;n\pi \frac{c_{q}}{d_{q}}$$

In the presence of the load, the resonance condition is

(107) $${i\omega m}_{R}+\frac{\kappa_{R}}{i\omega }{+\xi }_{R}{+A}_{\mathrm{eff}}{\tilde{Z}}_{L}=0\;$$

Before solving this equation for ω, we turn it into a more familiar form, which is:

(108) $$\omega^{2}\;+\;\omega \left(-\left(\frac{{\;i\xi }_{R}}{m_{R}}\;+\;\frac{{iA}_{\mathrm{eff}}{\tilde{Z}}_{L}}{m_{R}}\right)\right)\;+\;\left(\;\frac{\kappa_{R}}{m_{R}}\right)\;=\;0\;$$

Again neglecting the dashpot, the shifted resonance frequency is:

(109) $${\tilde{\omega }}_{0,L}\;=\;\frac{{iA}_{\mathrm{eff}}{\tilde{Z}}_{L}}{{2m}_{R}}\;\pm \;\sqrt{{\left(\frac{{iA}_{\mathrm{eff}}{\tilde{Z}}_{L}}{{2m}_{R}}\right)}^{2}\;+\;\frac{\kappa_{R}}{m_{R}}\;}\approx \;\frac{{iA}_{\mathrm{eff}}{\tilde{Z}}_{L}}{{2m}_{R}}\;+\;\sqrt{\frac{\kappa_{R}}{m_{R}}}\;$$

In the second step, the meaningful solution (out of the two solutions of the quadratic equation in ω) was selected. The quadratic term under the root was neglected because the load impedance is small. With $m_{q}{\;=\;\rho }_{q}d_{q}$ and $Z_{q}\;=\;{\left(\rho_{q}G_{q}\right)}^{1/2}$, Equation (109) turns into:

(110) $$\frac{\Delta \omega }{\omega_{0}}\;=\;\frac{\omega_{0,L}\;-{\;\omega }_{0}}{\omega_{0}}\;=\;\frac{{iA}_{\mathrm{eff}}{\tilde{Z}}_{L}}{{2m}_{R}}\sqrt{\frac{m_{R}}{\kappa_{R}}}\;=\;i\frac{A_{\mathrm{eff}}{\tilde{Z}}_{L}}{{2m}_{R}}\frac{d_{q}}{c_{q}n\pi }\;=\;\frac{i{\tilde{Z}}_{L}}{n\pi }\frac{1}{2\left(Z_{\mathrm{eff}}m_{q}/2\right)}\frac{d_{q}}{c_{q}}\;=\;\frac{1}{n}\frac{i}{{\pi Z}_{q}}{\tilde{Z}}_{L}\;.$$

This is the small-load approximation. 

### 8.5. Amplitude of Oscillation, Effective Area

The effective turns ratio, ϕ, of the transformer in Figure 42B leads to a relation between the current into the electrodes and the velocity at the resonator surface. On resonance, the velocity, ${\hat{v}}_{S}$, and the current, $\hat{I}$, are related as [199]:

(111) $${\hat{v}}_{S}\;=\;i\omega {\hat{u}}_{S}\;=\;\frac{1}{2\phi }\hat{I}\approx \frac{d_{q}}{{2e}_{26}A_{\mathrm{eff}}}\frac{{\hat{U}}_{\mathrm{ext}}}{{\tilde{Z}}_{\mathrm{tot}}}\;\;$$

A factor of 1/2 enters because the current is proportional to the difference in velocity between the front and the back (which is 2${\hat{v}}_{S}$). The relation between the current into the electrodes, $\hat{I}$, and the nominal external voltage, ${\hat{U}}_{\mathrm{ext}}$, may be nontrivial. Further complicating the situation, vector network analyzers often control the power (in units of dBm), rather than voltage or current. With 5 MHz resonators on the fundamental, an area of $A_{\mathrm{eff}}$ = 10 mm^2^, a voltage of 100 mV, and a motional resistance of $R_{1}$ = 500 Ω (typical for experiments in liquids), an amplitude of oscillation of 0.1 nm results. The shear angle is ${\hat{u}}_{S}/\delta$, which is below 10^−3^. A more detailed calculation of the amplitude would have to account for energy trapping. The current through the motional branch may also be affected by the analyzer’s output resistance and by the current through the parallel capacitance, $C_{0}$. For that reason, “≈” was written on the right-hand side in Equation (111). 

If the resonator is under voltage control, one may also remember the relation:

(112) $${\hat{u}}_{S}\approx \frac{4}{{\left(n\pi \right)}^{2}}d_{26}Q\;{\hat{U}}_{\mathrm{ext}}\;=\;1.25\;\frac{\mathrm{pm}}{V}\;\frac{Q}{n^{2}}{\hat{U}}_{\mathrm{ext}}\;$$

$d_{26}$ = 3.1 × 10^−12^ m/V is the piezoelectric strain coefficient of AT-cut quartz. Equation (112) was confirmed experimentally in [200]. 

The transformer’s effective turns ratio, $\phi {\;=\;e}_{26}A_{\mathrm{eff}}{/d}_{q}$, also leads to an equation for the plate’s effective area, which is:

(113) $$A_{\mathrm{eff}}\;\approx \;\frac{n\pi }{32\cdot Z_{q}d_{26}^{2}f_{0}^{\;2}}\frac{1}{{QR}_{1}}\;$$

For the proof see Section 7.4 in [5]. The same approximations as in Equation (111) apply.

### 8.6. Modal Mass, Sauerbrey Equation for Plates with Energy Trapping

Flexural admixtures not only affect measurements in liquids (because of compressional waves), but also measurements of the mass of a film in the dry. Start from the classical formula for the resonance frequency of a harmonic oscillator, $\omega_{0}\;\approx \;{\left(\kappa_{R}/m_{R}\right)}^{1/2}$. This formula holds for discrete objects. Applied to elastic bodies vibrating in some vibration mode, equivalent parameters must be defined. The definition of $\kappa_{R}$ is unessential here. Write the total mass as $m_{R}+A_{\mathrm{eff}}m_{f}$ with $m_{R}$ the “modal mass” and $m_{f}$ the film’s mass per unit area. Assuming $A_{\mathrm{eff}}m_{f}\ll m_{R}$ and applying the Taylor expansions (1 + ε)^1/2^ ≈ 1 + ε/2 and (1 + ε)^–1^ ≈ 1 – ε leads to:

(114) $$\begin{array}{ll} \Delta f & =\frac{\Delta \omega }{2\pi }=\frac{1}{2\pi }\left(\sqrt{\frac{\kappa_{R}}{m_{R}{+A}_{\mathrm{eff}}m_{f}}}-\sqrt{\frac{\kappa_{R}}{m_{R}}}\right)\\ & =\frac{1}{2\pi }\sqrt{\frac{\kappa_{R}}{m_{R}}}\left(\frac{1}{\sqrt{{1+A}_{\mathrm{eff}}m_{f}{/m}_{R}}}-1\right)\\ & \approx \;\frac{1}{2\pi }\sqrt{\frac{\kappa_{R}}{m_{R}}}\left(\frac{1}{{1+A}_{\mathrm{eff}}m_{f}{/(2m}_{R})}-1\right)\\ & \approx \frac{1}{2\pi }\sqrt{\frac{\kappa_{R}}{m_{R}}}\left({1-A}_{\mathrm{eff}}m_{f}{/(2m}_{R})-1\right){=-f}_{\mathrm{ref}}\left(\frac{A_{\mathrm{eff}}m_{f}}{{2m}_{R}}\right)\; \end{array}$$

At first glance, Equation (114) appears to differ by a factor of 1/2 from the Sauerbrey result, which is:

(115) $$\frac{\Delta f}{f_{\mathrm{ref}}}\;=\;-\frac{m_{f}}{m_{q}}\;=\;-\frac{A_{\mathrm{eff}}m_{f}}{A_{\mathrm{eff}}m_{q}}\;$$

$m_{q}$ is the mass per unit area of the resonator plate. The comparison shows that $m_{R}$ is only half the mass of the resonator plate, $A_{\mathrm{eff}}m_{q}$. This is so because a plate in thickness-shear motion contains nodal planes. The kinetic energy is 1/2$A_{\mathrm{eff}}m_{q}\langle v_{S}^{2}\rangle {}_{t,V}$. Averaging occurs over time, *t*, and volume, *V*. Volume averaging produces a factor of 1/2 because $\langle \cos^{2}\left(x\right)\rangle =1/2$, which implies $m_{R}$ = $A_{\mathrm{eff}}$*m*_q_/2. 

Importantly, this factor of 1/2 turns into some other numerical factor when flexural modes are present because the flexural motion does not vanish at the nodal planes of the shear motion. The modal mass increases and –Δ*f*/*n* decreases in consequence. This effect slightly modifies the Sauerbrey equation. 

**Figure 44:**
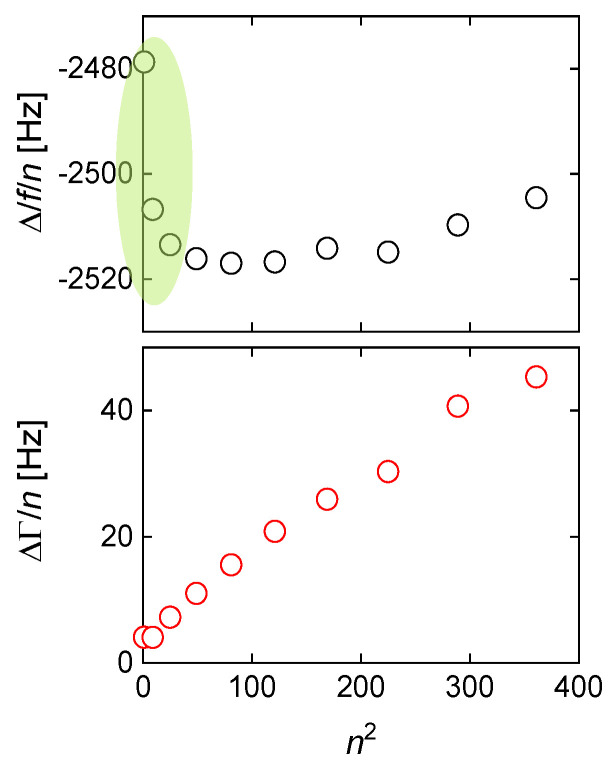
Overtone-normalized shifts of frequency and bandwidth obtained after depositing a polymer film with a thickness of about 500 nm onto the resonator plate. The area in green denotes values of –Δ*f*/*n* which deviate from the prediction by Equation (46). The deviations go back to flexural motion. Adapted from [80].

Figure 44 shows an experimental example. The sample was a polymer film in air. Because this film is viscoelastic, −Δ*f*/*n* decreases in proportion to $n^{2}$ at large *n* (see Equation (46)). From the slope, one can infer ${J_{f}}^{\prime }$. If interpreted with Equation (45), the positive slope would indicate negative shear compliance, but Equation (46) solves that problem. Regardless of the slope: The low overtones must be excluded from this analysis, because the respective vibration modes have an increased modal mass.

The above argument started from $\omega_{0}\approx \;{\left(\kappa_{R}{/m}_{R}\right)}^{1/2}$. The argument can also start from the tensor form of the small-load approximation. The denominator in Equation (100) is proportional to the modal mass.

## 9. Combined Instruments

Because the QCM is simple, it can be easily combined with other instruments. For instance, it is quite trivial to push a QCM sensor against the prism of an ATR-IR spectrometer and obtain an IR spectrum from the sample on the resonator with monolayer sensitivity (ex-situ, evidently). One can rather easily do AFM, XPS, and Raman spectroscopy on a QCM, either in-situ or ex-situ. The examples are numerous. Two problems are worth a mention:

- When combining a QCM with an AFM [201], the QCM does not usually respond to the contact with the AFM tip because the contact is too small. That can be understood from Equation (70) together from the Mindlin result for the transverse contact stiffness, which is $\kappa_{P}$ = 2G* $r_{C}$ with G* an effective modulus and $r_{C}$ the contact radius [117] (see Section 5). Inserting values (G* ≈ 10 GPa, $r_{C}$ ≈ 5 nm) leads to a frequency shift below 0.1 Hz (at 5 MHz). An AFM tip tapping onto the resonator amounts to a nanoscopic object perturbing the motion of a macroscopic object. It does so, in principle, but the effects usually disappear in the noise. In the reverse direction (the QCM perturbing the motion of the tip), there is a strong influence [202]. However, experiments of this kind can also be done with any other actuator. Nanoscale dynamical-mechanical studies based on an AFM tip in contact with a vibrating substrate are commonplace ([203] and others).
- In-situ combination with dielectric spectroscopy [204,205,206,207] or electrical cell-substrate impedance spectroscopy (ECIS [208]) is possible. A difficulty arises, when the sample requires an oxidic substrate, such as SiO_x_, because the commercially available SiO_x_ coatings may be too thick. The electric field then does not reach to the sample. More technically, the coating’s capacitance, *C*_SiOx_, is so small, that its impedance dominates the sample’s overall electrical impedance. The properties of the sample are then masked by the term 1/(iω*C*_SiOx_). Thin dielectric coatings are needed.

The text proceeds with two particularly well studied combinations, which are the electrochemical QCM and the combination with optical reflectometry.

### 9.1. The Electrochemical QCM (EQCM) 

Among the combinations with other techniques, the combination with electrochemistry is most advanced. For early works see [16,17,18,19,209]. More recent reviews are provided in [210,211,212].

Electrogravimetry can be rather complicated and we do not elaborate on the details. Matters are transparent as long as one sticks to electrodeposition [213] or electroetching [214] with layer thicknesses of many nanometers. The QCM then operates in the gravimetric regime. The charge passed through the electrode surface may be converted to a mass, using Faraday’s law [103,215]. If this mass equals the mass as inferred from the Sauerbrey equation, the “current efficiency” is unity. If this current efficiency is less than unity, one suspects side reactions or soft layers. For soft layers, the bandwidth and the overtone dependence of −Δ*f*/*n* can be analyzed to derive the layer’s viscoelastic constants (Equation (52), [104]). If the current efficiency is larger than unity, roughness may be a reason [216].

Unfortunately, there are numerous sources of artifacts at the low end of the QCM’s sensitivity range. Among these are the viscoelasticity of the double layer [48,217], nanobubbles [218], slip [219], roughness [216], piezoelectric stiffening [176,220], and static stress, which bends the plate [53]. 

The difficulties admitted, there is additional information available for interpretation. Firstly, there is the electrical current as a function of time (or the voltage, in case the experiment occurs under current control). If the experiment occurs outside the gravimetric range, charge and frequency shift are not simply related by Faraday’s law, but they should still be related in some other way. Also, kinetics comes to help. The fast QCM discussed in Section 3.5 resolves the kinetics with a time resolution down to 100 µs. The response times determined this way give clues to the nature of the processes involved. Finally, the frequency resolution can be improved by running the experiment repetitively and accumulating data. If a certain process occurs in response to a change in electrode potential on the time scale of, for instance, one second, accumulation overnight improves the resolution in frequency by a factor of about (40000)^1/2^ (by *N*^1/2^ with *N* the number repeats). The frequency resolution readily drops to below 1 mHz. A caveat: For the conventional QCM, dirt, scratches, or even small bubbles are not necessarily detrimental because the QCM averages over the entire active area (~10 mm^2^). A minute response (of a few mHz) to a jump in electrode potential, however, may entirely result from such local heterogeneities. 

The Paris group has exploited the combination of electrochemical impedance spectroscopy (EIS) with the QCM in numerous papers [103] and others. They call the technique “AC electrogravimetry”. The electrode potential is modulated sinusoidally. The QCM is driven by an oscillator circuit, the output of which is fed into a frequency-to-voltage converter. The respective voltage oscillates with the modulation frequency. It is displayed together with the electrical current, often in the same diagram, often showing similar features.

The molecular details of electrochemistry can be enormously complex. The EQCM (similar to EIS) only measures integral quantities (such as mass transfer, area-averaged softness, and area-averaged roughness) and their kinetics. It can still aid the structural investigations (examples in [221,222]) and provide for constraints to the models.

### 9.2. Combination with Optical Reflectometry

With regard to label-free biosensing, SPR spectroscopy [15] outperforms the QCM insofar, as SPR has a lower limit of detection and smaller baseline drifts. Irrespective of the competition, there are interesting conceptual similarities between the QCM and optical reflectometry. Also, there are benefits in the combination of the two [93,151,223].

**Figure 45:**
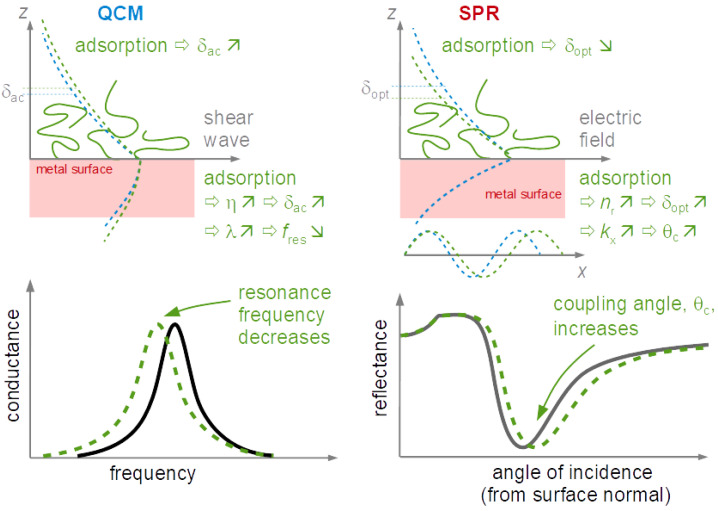
Both the QCM and SPR spectroscopy exploit the shift of a resonance condition. The peak in the conductance curve is the analog of a dip in the reflectance curve. The resonance frequency is replaced by the coupling angle (that is, the angle of incidence with minimum reflectance). The coupling angle is related to the plasmon’s *k*_x_-vector. The shift in the coupling angle is proportional to the adsorbed amount.

Similar to the QCM, SPR spectroscopy exploits a shift of a resonance condition, where the frequency is replaced by the *k*_x_-vector of the surface plasmon. The analogy is illustrated in Figure 45. 

If the adsorbed layer is thinner than the decay depth of the plasmon, $\delta_{\mathrm{opt}}$, (which happens to be similar to the decay depth of the shear wave of the QCM), the shift in $k_{x}$ is proportional to the 1st invariant of the refractive index profile, $J_{1}$, given as [224]:

(116) $$J_{1}\;=\;\int_{-\infty }^{\infty }\frac{\left(n_{\mathrm{sub}}^{2}\;-{\;n}_{r}^{2}\left(z\right)\right)\left(n_{r}^{2}\left(z\right)-n_{\mathrm{bulk}}^{2}\right)}{n_{r}^{2}\left(z\right)}dz$$

Subscripts *sub* and *bulk* denote the substrate and the bulk liquid, respectively. $n_{r}$ is the refractive index. $n_{r}^{2}$ is equal to the dielectric constant at optical frequencies, ε. While ε and $n_{r}^{2}$ are complex, in principle, they are treated as real parameters, here. In SPR spectroscopy, the substrate is a metal (often gold), which has a large, negative ε. The term $n_{\mathrm{sub}}^{2}-{\;n}_{r}^{2}\left(z\right)$ may be approximated as $n_{\mathrm{sub}}^{2}-{\;n}_{\mathrm{bulk}}^{2}$ (equal to $\varepsilon_{\mathrm{sub}}-{\;\varepsilon }_{\mathrm{bulk}}$) and be pulled out of the integral. Neglecting prefactors, the shift in $k_{x}$ induced by the adsorbate obeys [225]:

(117) $${\Delta k}_{x}\;\propto \;\;\frac{2\pi }{\lambda }\int_{0}^{\infty }\left[\frac{n_{r}^{2}\left(z\right)\;{\;n}_{\mathrm{bulk}}^{2}}{n_{r}^{2}\left(z\right)}\right]dz\;$$

**Figure 46:**
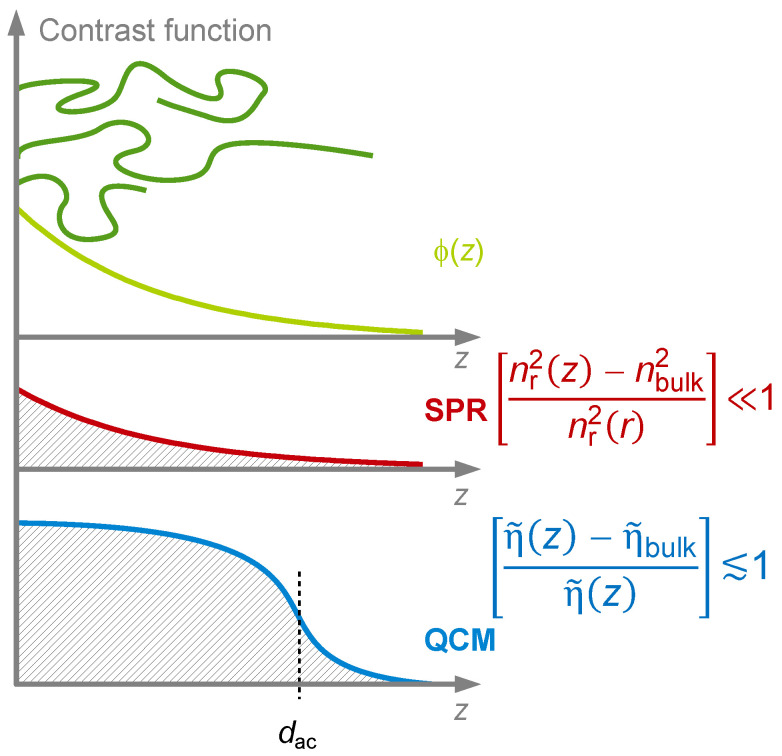
The contrast functions from Equation (58) and Equation (117) are similar, algebraically. The difference is in the numerical values. In optics, the refractive index varies by a few percent. The denominator is nearly constant. Because the change in $n_{r}^{2}$ is about proportional to concentration, the integral of the contrast function is roughly proportional to the adsorbed amount. In the case of the QCM, the viscosity of an adsorbate layer easily exceeds the viscosity of the bulk by a factor of 10 or more. The contrast function saturates to unity and the integral of the contrast function is about proportional the acoustic thickness, $d_{\mathrm{ac}}$. $d_{\mathrm{ac}}$ is the distance, at which the sample’s viscosity has decreased to about twice the viscosity of the bulk.

Both Δ*f* + iΔΓ (in QCM experiments, Equation (58)) and Δ*k*_x_ (in SPR spectroscopy, Equation (117)) depend on an integral of a response function. Algebraically, the response functions are similar. Differing from SPR spectroscopy, the response function to be used for the QCM saturates to unity. This happens whenever the viscosity in the layer is much larger than the viscosity of the bulk. The contrast function in optics, on the contrary, is mostly proportional to concentration, because the refractive index increment, d$n_{r}$/d*c*, is small. The SPR response is roughly proportional to the adsorbed amount, while the QCM probes the distance to the surface, at which the contrast function finally falls out of saturation ($d_{\mathrm{ac}}$ in Figure 46). When the layer expands, the SPR response changes by a small amount because the increase in thickness is compensated by a decrease in concentration. The QCM mostly notices an increase in acoustic thickness. These remarks apply to all variants of optical reflectometry. Because SPR and QCM see the adsorbed amount and the thickness, respectively, the degree of swelling may be inferred from the combination of SPR and QCM (more generally, from the combination of optical reflectometry with shear-wave acoustic reflectometry). 

SPR can be combined with the QCM in-situ, but that requires grating coupling on the surface of a resonator crystal, which is expensive [226,227]. Ellipsometry is an option [151] and others, but ellipsometry can be challenging in the details, especially when the beam passes through windows. Given that the electrodes often consist of gold, variants of optical reflectometry can be employed, which are simpler than full-fledged ellipsometry [228,229]. If it shall be SPR on the side of optical reflectometry, it can be Love wave sensors on the side of shear-wave acoustics [230]. Love wave sensors are a variant of the surface acoustic wave devices [231,232]. For reasons in the details, these are more easily combined with SPR than the QCM. The remarks above on saturation of the contrast function apply to Love waves and to shear waves as excited by the QCM in essentially same way [233]. 

A last remark: The comparison between data acquired with optical reflectometry and with shear-wave acoustic reflectometry can be based on separate experiments with separate instruments, as long as the sample preparation is sufficiently reproducible [234].

## Data Availability

Data sharing not applicable. No new data were created or analyzed in this study. Data sharing is not applicable to this article.

## Appendix A. Summary of Essential Equations

Small-load approximation	$\frac{\Delta \tilde{f}}{f_{0}}=\frac{i}{\pi Z_{q}}{\tilde{Z}}_{L}=\frac{i}{\pi Z_{q}}\frac{\langle -{\hat{\sigma }}_{S}\rangle_{\mathrm{area}}}{{\hat{v}}_{S}}$
Gordon-Kanazawa equation, semi-infinite viscoelastic medium	$\frac{\Delta \tilde{f}}{f_{0}}=\frac{i}{\pi Z_{q}}{\tilde{Z}}_{L}=\frac{-1+i}{\sqrt{2}\pi Z_{q}}\sqrt{\omega }\sqrt{\rho \tilde{\eta }}\;\;\;\;\;\;i\omega \rho \tilde{\eta }=\rho \tilde{G}=-{\left(\pi Z_{q}\frac{\Delta \tilde{f}}{f_{0}}\right)}^{2}$
Sauerbrey equation, rigid sample	$\frac{\Delta \tilde{f}}{f_{0}}\approx -\frac{2nf_{0}}{Z_{q}}m_{f}=-n\frac{\langle m_{f}\rangle {}_{\mathrm{area}}}{m_{q}}\;\;\;\;\;\;\;\;\frac{\Delta f}{f_{\mathrm{ref}}}=-\frac{m_{f}}{m_{q}}$
Viscoelastic film in air	$\frac{\Delta \tilde{f}}{f_{0}}=\frac{-1}{\pi Z_{q}}{\tilde{Z}}_{f}\tan\left({\tilde{k}}_{f}d_{f}\right)$
Thin film in air	$\frac{\Delta \tilde{f}}{f_{0}}=\frac{-\omega m_{f}}{\pi Z_{q}}\left[1+\frac{{\left(n\pi \right)}^{2}}{3}\left(\frac{{\tilde{J}}_{f}}{\rho_{f}}Z_{q}^{2}-1\right){\left(\frac{m_{f}}{m_{q}}\right)}^{2}\right]$
Viscoelastic film in liquid	$\frac{\Delta \tilde{f}}{f_{0}}=\frac{-{\tilde{Z}}_{f}}{\pi Z_{q}}\frac{{\tilde{Z}}_{f}\tan\left({\tilde{k}}_{f}d_{f}\right)-i{\tilde{Z}}_{\mathrm{bulk}}}{{\tilde{Z}}_{f}+i{\tilde{Z}}_{\mathrm{bulk}}\tan\left({\tilde{k}}_{f}d_{f}\right)}$
Thin film in liquid	$\begin{array}{c} \frac{\Delta \tilde{f}}{f_{0}}\approx \frac{-\omega m_{f}}{\pi Z_{q}}\left[1-n{\tilde{J}}_{f}(\omega )2\pi i\frac{1}{\rho_{f}}f_{0}\rho_{\mathrm{bulk}}\eta_{\mathrm{bulk}}\right]\\ \frac{\Delta \Gamma }{-\Delta f}\approx {J_{f}}^{\prime }\omega \eta_{\mathrm{bulk}} \end{array}$
Thin adsorbate in liquid	$\frac{\Delta \tilde{f}}{f_{0}}=\frac{-\omega }{{\pi Z}_{q}}\rho_{\mathrm{bulk}}\int_{0}^{\infty }\left[\frac{\rho \left(z\right)}{\rho_{\mathrm{bulk}}}-\frac{\eta_{\mathrm{bulk}}}{\tilde{\eta }\left(z\right)}\right]\;dz$
Point contacts	$\Delta \tilde{f}=\frac{1}{n}\frac{n_{P}}{A_{\mathrm{eff}}}\frac{1}{2\pi^{2}Z_{q}}{\tilde{\kappa }}_{P}$
Time averaging, large amplitudes, nonlinear response	$\frac{\Delta \tilde{f}}{f_{0}}=\frac{i}{\pi Z_{q}}\frac{2{\langle -{\hat{\sigma }}_{S}(t)\exp\left(i\omega t\right)\rangle }_{\mathrm{area},\mathrm{time}}}{{\hat{v}}_{S}}$
Small-load approximation in tensor form	$\Delta \tilde{f}\;\approx \;\frac{i}{{4\pi \rho }_{q}}\frac{\sum_{ijk}\int_{\mathrm{Surface}}{\hat{u}}_{S,i}^{\left(0\right)}\left(r_{S}\right)n_{j}{\tilde{Z}}_{L,ijk}\left(r_{S}\right){\hat{u}}_{S,k}^{\left(0\right)}\left(r_{S}\right)\;d^{2}r_{S}}{\sum_{i}\int_{\mathrm{Volume}}{\hat{u}}_{i}^{\left(0\right)}\left(r\right)\;{\hat{u}}_{i}^{\left(0\right)}\left(r\right)d^{3}r}$
Piezoelectric stiffening	$\frac{\Delta {\tilde{f}}_{\mathrm{PE}}}{f_{0}}=\frac{i}{\pi Z_{q}}\left[\frac{4e_{26}^{2}A_{\mathrm{eff}}}{d_{q}^{2}}\right]\left({\left(i\omega C_{0}+{\tilde{Z}}_{\mathrm{ext}}^{-1}\right)}^{-1}-{\left(i\omega C_{0}+{\tilde{Z}}_{\mathrm{ext},\mathrm{ref}}^{-1}\right)}^{-1}\right)$

## Appendix B. Glossary of Variables and Relations

**Variable**	**Definition**		
~	Tilde: a complex parameter		
^	Hat: a complex amplitude		
$A_{\mathrm{eff}}$	Effective area of the resonator	$A_{\mathrm{eff}}=\frac{n\pi }{{32Z}_{q}d_{26}^{2}f_{0}^{\;2}}\frac{G_{\max}}{Q}$	(A1)
$C_{0}$	Resonator’s electrical capacitance (parallel capacitance)	$C_{0}=\frac{\varepsilon_{q}\varepsilon_{0}A_{\mathrm{electrode}}}{d_{q}}$	(A2)
$C_{1}$	Motional capacitance	$C_{1}\;=\;\frac{8\phi^{2}}{{n\pi A}_{\mathrm{eff}}Z_{q}}\frac{1}{{2\pi nf}_{0}}\;\;\;\;\;\mathrm{with}\;\;\;\;\;\phi \;=\;\frac{A_{\mathrm{eff}}d_{26}}{d_{q}}$ $C_{1}\;=\;\frac{{8k}_{t,\mathrm{eff}}^{2}}{{\left(n\pi \right)}^{2}}C_{0}{\;\;\;\;\;\mathrm{with}\;\;\;\;\;k}_{t,\mathrm{eff}}^{2}\;\approx \;0.8\%$	(A3)
*D*	Dissipation factor	$D=\frac{1}{Q}=\frac{2\Gamma }{f_{\mathrm{res}}}$	(A4)
$\tilde{G}$	Shear modulus	$\begin{array}{c} \tilde{G}=G^{\prime }+iG^{\prime \prime }=1/\tilde{J}={\left(J^{\prime }-iJ^{\prime \prime }\right)}^{-1}\;=\;i\omega \tilde{\eta }\\ \tilde{G}=|G|{\left(\cos\left(\delta_{L}\right)+i\sin\left(\delta_{L}\right)\right)}_{} \end{array}$ ${\tan(\delta }_{L})\;=\;G^{\prime \prime }/G^{\prime }\;=\;J^{\prime \prime }/J^{\prime }$	(A5)
$G_{q}$	Shear modulus of AT-cut quartz	$G_{q}$ ≈ 29 × 10^9^ Pa	
$\tilde{J}$	Shear compliance	$\tilde{J}=1/\tilde{G}=\left(J^{\prime }-{iJ}^{\prime \prime }\right)$	(A6)
$L_{1}$	Motional inductance	$L_{1}=\frac{1}{4\phi^{2}}A_{\mathrm{eff}}\frac{m_{q}}{2}\;\;\;\;\;\;\phi =\frac{A_{\mathrm{eff}}e_{26}}{d_{q}}$	(A7)
*Q*	Quality factor	$Q=\frac{f_{\mathrm{res}}}{2\Gamma }$	(A8)
*R*_1_	Motional resistance, the inverse of *G*_max_ (Equation (13))		
$\tilde{Z}$	Shear-wave impedance	$\tilde{Z}=\rho \tilde{c}=\sqrt{\rho \tilde{G}}=\sqrt{i\omega \rho \tilde{\eta }}=\frac{i\tilde{\eta }\tilde{k}}{\omega }$	(A9)
${\tilde{Z}}_{L}$	Load impedance	${\tilde{Z}}_{L}=\frac{-{\hat{\sigma }}_{S}}{{\hat{v}}_{S}}$	(A10)
$Z_{q}$	Shear-wave impedance of AT-cut quartz	$Z_{q}$ = 8.8 × 10^6^ kg m^−2^ s^−1^	
${\tilde{c}}_{f}{,\;c}_{q},\;{\tilde{c}}_{\mathrm{bulk}}$	Speed of shear sound	$\tilde{c}=\sqrt{\tilde{G}/\rho }$	
$d_{26}$	Piezoelectric strain coefficient of AT-cut quartz	$d_{26}$ = 3.1 × 10^−12^ m/V	
$e_{26}$	Piezoelectric stress coefficient of AT-cut quartz	$e_{26}=\;d_{26}G_{q}$ = 9.65 × 10^−2^ C/m^2^	
$d_{f},\;d_{q}$	Thickness of film and resonator		
${\tilde{k}}_{f}{,\;k}_{q},\;{\tilde{k}}_{\mathrm{bulk}}$	Wave vector in the film, the resonator plate, and the bulk	${\tilde{k}}_{\mathrm{bulk}}=\frac{\omega }{{\tilde{c}}_{\mathrm{bulk}}}=\sqrt{\frac{{\omega \rho }_{\mathrm{bulk}}}{{i\eta }_{\mathrm{bulk}}}}=\sqrt{-2i}\frac{1}{\delta }=\left(1-i\right)\sqrt{\frac{{\omega \rho }_{\mathrm{bulk}}}{{2\eta }_{\mathrm{bulk}}}}$	(A11)
$k_{t}$	Electromechanical coupling coefficient	$k_{t,\mathrm{ideal}}^{2}=\frac{{\;e}_{26}}{\varepsilon_{q}\varepsilon_{0}G_{q}}$ $k_{t,\mathrm{eff}}^{2}=\frac{C_{1}}{C_{0}}\frac{{\left(n\pi \right)}^{2}}{8}$	(A12)
$f_{\mathrm{cen}}$	A frequency in the center of the QCM’s frequency range (Section 4.7)		
$f_{\mathrm{ref}}$	Resonance frequency in reference state		
$f_{\mathrm{res}}$	Resonance frequency	$f_{\mathrm{res}}{=nf}_{0}$	
$f_{0}$	Fundamental frequency	$f_{0}=\frac{Z_{q}}{{2m}_{q}}$	
$m_{f}\;{,\;m}_{q}$	Mass per unit area of the film and the resonator plate	$m_{q}{=\rho }_{q}d_{q}=\frac{Z_{q}\lambda }{2}=\frac{1}{2}\frac{Z_{q}c_{q}}{f_{0}}=\frac{Z_{q}}{{2f}_{0}}$	(A13)
$m_{R}$	Effective mass of a resonator	${\mathrm{parallel}\;\mathrm{plate}:\;m}_{R}=\frac{A_{\mathrm{eff}}m_{q}}{2}$	(A14)
*n*	Overtone order		
$n_{P}$	Number of particles in contact with the resonator		
$n_{r}$	Refractive index		
$u,\;\hat{u}$ ${\;u}_{S},\;{\hat{u}}_{S}$	Displacement, amplitude of displacement (^) subscript *S*: at the surface	$\hat{u}=\frac{\hat{v}}{i\omega }\;\;\;\;\;\;{\hat{v}}_{S}=\frac{d_{q}}{2e_{26}A_{\mathrm{eff}}}\hat{I}$	(A15)
$\;v,\;\hat{v}$, $v_{S},\;{\hat{v}}_{S}$	Velocity, amplitude of velocity (^)	$\hat{v}=i\omega \hat{u}$	(A16)
$\beta^{\prime },\;\beta^{\prime \prime }$ $\gamma^{\prime },\;\gamma^{\prime \prime }$	Power law exponents, Section 4.7	${J_{f}}^{\prime }\left(f\right)\;\approx {{\;J}_{f}}^{\prime }\left(f_{\mathrm{cen}}\right){\left(\frac{f}{f_{\mathrm{cen}}}\right)}^{\beta^{\prime }}$ ${G_{f}}^{\prime }\left(f\right)\;\approx {{\;G}_{f}}^{\prime }\left(f_{\mathrm{cen}}\right){\left(\frac{f}{f_{\mathrm{cen}}}\right)}^{\gamma^{\prime }}$ ${J_{f}}^{\prime \prime }\left(f\right)\;\approx {{\;J}_{f}}^{\prime \prime }\left(f_{cen}\right){\left(\frac{f}{f_{\mathrm{cen}}}\right)}^{\beta^{\prime \prime }}$ ${G_{f}}^{\prime \prime }\left(f\right)\;\approx {{\;G}_{f}}^{\prime \prime }\left(f_{\mathrm{cen}}\right){\left(\frac{f}{f_{\mathrm{cen}}}\right)}^{\gamma^{\prime \prime }}$	(A17)
$\tilde{\eta }$	Viscosity	$\tilde{\eta }=\frac{\tilde{G}}{i\omega }=\eta^{\prime }-i\eta^{\prime \prime }$	(A18)
Γ	Imaginary part of resonance frequency, half-band half width		
ΔR+iΔX	Load impedance in electrical units (in older publications)	$\Delta R+i\Delta X=\frac{A_{\mathrm{eff}}}{4\phi^{2}}{\tilde{Z}}_{L}=-i\frac{\pi }{16}\frac{Z_{q}^{3}}{A_{\mathrm{eff}}e_{26}^{2}\varepsilon_{q}^{2}f_{0}^{3}}(\Delta f+i\Delta \Gamma )$	(A19)
$\varepsilon_{q}$	Dielectric constant of AT-cut quartz	$\varepsilon_{q}$ = 4.54	
$\kappa_{R}$	Effective spring constant	$\kappa_{R}=\frac{A_{\mathrm{eff}}G_{q}}{d_{q}}\frac{{\left(n\pi \right)}^{2}}{2}=\frac{{\left(n\pi \right)}^{2}}{2}\kappa_{q,\mathrm{stat}}$	(A20)
$\rho_{q}$	Density of α-quartz	$\rho_{q}$ = 2.65 × 10^3^ kg/m^3^	
$\sigma ,\;\hat{\sigma }$, $\sigma_{S},\;{\hat{\sigma }}_{S}$	Tangential stress, amplitude thereof		
$\xi_{R}$	Effective drag coefficient		

## Appendix C. Python Code

### Appendix C.1. Calculation of Δf + iΔΓ Resulting from a Continuous Viscoelastic Profile

The code in Box A1 prescribes a function $\tilde{G}\left(z\right)$ and the derivative $d\tilde{G}(z)/dz$ (calculated analytically). The density is assumed as constant. The code solves the wave equation (Equation (64)), normalizes the solution to unit velocity at the surface, and evaluates the ratio of stress to strain at the surface (Equation (63)).

Two comments:

- The algorithm solve_ivp from scipy requires the 2nd-order differential equation from Equation (64) to be turned into a system of two 1st-order equations. This is achieved by defining the function ${\hat{u}}^{\prime }(z)\;=\;d\hat{u}(z)/dz$. The 1st-order equations are:
  (A21) $$\frac{d}{dz}\hat{u}\;=\;{\hat{u}}^{\prime }\;\frac{d}{dz}{\hat{u}}^{\prime }\;=\;-\frac{{\rho \omega }^{2}}{\tilde{G}}\hat{u}\;-\;\frac{1}{\tilde{G}}\frac{d\tilde{G}}{dz}{\hat{u}}^{\prime }$$
- One choses an “initial” condition at large *z* and integrates backwards. In this way, it is ensured that the solution decays to zero as *z*→∞. The initial condition consists of values for the velocity and the velocity gradient. The velocity can take any value. It later cancels during normalization. The velocity gradient is the velocity multiplied by i${\tilde{k}}_{0}$ where ${\tilde{k}}_{0}\;=\;{\left(i\omega \rho /\eta \right)}^{1/2}$ is the wave number in the bulk liquid.

Box A1 Calculation of Δ*f* + iΔΓ resulting from a continuous viscoelastic profile. **import** numpy **as** np **from** scipy**.**integrate **import** solve_ivp z_max **=** 1500e-9**;** z_infl **=** 300e-9**;** width **=** 100e-9**;** eta_Liq **=** 1e-3**;** rho **=** 1e3**;** Gp_surf **=** 1e5**;** Gpp_surf **=** 1e4**;** f0 **=** 5e6**;** om **=** 2 ***** np**.**pi ***** f0**;** Zq **=** 8.8e6**; **ik0 = 1j * (1 + 1j)/252e-9 **def** G**(**z_rev**):**# "rev" because the z-scale is reversed    z **=** z_max **-** z_rev    Gp  **=** Gp_surf  ***** 1.**/**2.***(**1. **-** np**.**tanh**((**z**-**z_infl**)/**width**))**    Gpp **=** Gpp_surf ***** 1.**/**2.***(**1. **-** np**.**tanh**((**z**-**z_infl**)/**width**))+**om*****eta_Liq    **return** Gp **+** 1j ***** Gpp **def**G_deriv_rev**(**z_rev**):**    z **=** z_max **-** z_rev    Gpderiv_rev **= **Gp_surf *****1.**/**2.***(-**1.**/**np**.**cosh**((**z**-**z_infl**)/**width**)/**width**)**    Gppderiv_rev**= **Gpp_surf*****1.**/**2.***(-**1.**/**np**.**cosh**((**z**-**z_infl**)/**width**)/**width**)**    **return -(**Gpderiv_rev **+** 1j*****Gppderiv_rev**)** # “-” : z-scale is reversed **def** second_deriv_of_u**(**z_rev**,** u_uprime**):**  # u_uprime : veloc., deriv.    u**,** uprime **=** u_uprime    **return [**uprime**,\**        -rho*****om******2**/**G**(**z_rev**)***u**-**G_deriv_rev**(**z_rev**)/**G**(**z_rev**)***uprime**]** GridPoints **=** np**.**arange**(**0**,**z_max**,**10e-9**)** u_ini **= [**1**+**0*****1j**,**ik0**]** #ini vals, solution is later normalized to 1 sol **=** solve_ivp**(**second_deriv_of_u**, [**0**,** z_max**],**u_ini**,**t_eval**=** GridPoints**)** u **=** sol**.**y **/** sol**.**y**[**0**,-**1**]** # normalize, so that velocity at surface is 1 ZL **=** G**(**z_max**)***u**[**1**,-**1**] / (**1j ***** om ***** u**[**0**,-**1**])** dfc **=** f0 ***** 1j **/(**np**.**pi ***** Zq**) *** ZL **print****(**’Df: {:.3f} Hz DG: {:.3f} Hz’**.**format**(**dfc**.**real**,**dfc**.**imag**))**

### Appendix C.2. Calculation of Δf + iΔΓ for a Film in Air, Starting from the Lu-Lewis Equation

The Python code in Box A2. computes Δ*f* + iΔΓ from the Lu-Lewis equation (Equation (40)). dfc_LuLewis might be recomputed numerous times and used as a model for fitting. The free parameters are $\rho_{f}$ (rho_f), ${G_{f}}^{\prime }$ (Gprime_f), tan($\delta_{f}$) (tandel_f), and $d_{f}$ (thickness_f).

Box A2 Calculation of Δ*f* + iΔΓ for a film in air, starting from the Lu-Lewis equation. **import** numpy **as** np**; from** scipy **import** optimize **def** Calc_dfc_SLA**(**rho_f**,**Gprime_f**,**tandel_f**,**thickness_f**):**    G_f **=** Gprime_f *** (**1 **+** 1j ***** tandel_f**);** Z_f **= (**rho_f ***** G_f**)****0.5**;**    c_f **= (**G_f**/**rho_f**)****0.5**;**           k_f **=** omegaref **/** c_f**;**    ZL **=** 1j ***** Z_f ***** np**.**tan**(**k_f*****thickness_f**)**    dfc_SLA **=** f0 ***** 1j **/ (**np**.**pi*****Zq**) *** ZL**;**    **return** dfc_SLA **def** Calc_dfc_LuLewis **(**rho_f**,**Gprime_f**,**tandelf**,**thickness_f**):**    G_f **=** Gprime_f *** (**1 **+** 1j ***** tandel_f**);**  Z_f **= (**rho_f ***** G_f**)****0.5**;**    c_f **= (**G_f**/**rho_f**)****0.5**;**     **def** fun**(**x**):**        dfcL **=** x[0] **+** 1j*****x[1]        k_f **= (**omegaref **+** 2*****np**.**pi*****dfcL**.**real**) /** c_f**;**        ZL **=** 1j ***** Z_f ***** np**.**tan**(**k_f*****thickness_f**)**        rhs_m_lhs **= -**1j*****Zq*****np**.**tan**(**np**.**pi*****dfcL**/**f0**) -** ZL        **return** rhs_m_lhs**.**real**,**rhs_m_lhs**.**imag     dfc_SLA **=** Calc_dfc_SLA**(**rho_f**,**Gprime_f**,**tandel_f**,**thickness_f**)**    sol **=** optimize**.**root**(**fun**, [**dfc_SLA**.**real**,**dfc_SLA**.**imag**])**    dfc_LuLewis **=** sol**.**x[0] **+** 1j***** sol**.**x[1]    **return** dfc_LuLewis f0 **=** 5e6**;** n **=** 1**;** fref **=** n*****f0**;** omegaref **=** 2*****np**.**pi*****fref**;** Zq **=** 8.8e6**;** rho_f **=** 1e3**;** thickness_f **=** 100e-9**;** Gprime_f **=** 1e7**;** tandel_f **=** 0.3**;** dfc_LuLewis **=** Calc_dfc_LuLewis** (**rho_f**,**Gprime_f**,**tandel_f**,**thickness_f**)** **print(**’Df = ’ **+** str**(**np**.**round**(**dfc_LuLewis**.**real**,**3**)))** **print(**’DG = ’ **+** str**(**np**.**round**(**dfc_LuLewis**.**imag**,**9**)))**

### Appendix C.3. Fit of a Box Profile to Experimental Data

The code in Box A3 reads in data from a file. The file is assumed to contain overtone-normalized frequency shifts and dissipation factors. Adjustments may be needed with regard to data format. The algorithm fits Equation (49) to the data. It accounts for viscoelastic dispersion. Following the remarks around Figure 24, $\beta^{\prime \prime }$ is fixed. The free parameters are $d_{f}$ (d_f), |*J*| (J_abs), tan($\delta_{L}$) (loss_tan), and $\beta^{\prime }$ (PL_exp_p).

Box A3 Fit of a box profile to experimental data. GuessPars **= [**3e-9**,**1e-6**,**0.7**,-**0.5**]** #[thickness, |J|,tan(del),PL exp J’] PL_exp_pp **= -**0.5**;** #Power law exponent for J’’ better be fixed Correct_for_Drift **=** ‘yes’**;** filename **=**‘test.txt’ 
**def** read_dfcs ()**:**
f **=** open(filename**,**’r’)**;** lines **=** f**.**readlines()**;** f**.**close()**;** times **=** np**.**zeros(len(lines)**-**1)**;** dfc_expt_in**=**np**.**zeros((len(lines)**-**1**,**len(ovt_ord_in))**,**dtype**=**complex)**;** **for** i **in** range(len(lines)**-**1)**:** columns **=** lines**[**i**+**1**].**split()**;** times**[**i**] =** np**.**float(columns [0]**.**replace(‘,’**,**’.’))**;** **for** i_ovt **in** range (len(ovt_ord_in))**:** dfc_expt_in**[**i**,**i_ovt**]=\**            np**.**float(columns**[**2 *****i_ovt**+**1**]**) ***** ovt_ord_in**[**i_ovt**]+**\ 1j*****2.5*****np**.**float(columns**[**2*****i_ovt**+**2**]**) ***** ovt_ord_in**[**i_ovt**];** **if** Correct_for_Drift **==** ‘yes’ **:** **for** j **in** range(1**,** len(times)) **:** **for** i_ovt **in** range(len(ovt_ord_in)) **:** dfc_expt_in**[**j**,**i_ovt**] -=** (1. **+** i_ovt)**/**len(ovt_ord_in)*****\ (dfc_expt_in**[**j**,**i_ovt**] -** dfc_expt_in**[**j**-**1**,**i_ovt**]**) dfc_expt **=** dfc_expt_in**[:,**1**:-**1**]**  # omit 5 MHz, ignore 55 MHz **return** times**,**dfc_expt **def**Calc_dfc_from_Box_Profile(ovt_order**,** Pars)**:** d_f **=** Pars[0]**;** J_abs **=** Pars[1]**;** loss_tan **=** Pars[2]**;** PL_exp_p **=** Pars[3]**;** f **=** ovt_order ***** f_fund**;** om **=** 2 ***** np**.**pi ***** f**;** J_f **=**            J_abs ***** np**.**cos(loss_tan) *****  (f**/**f_Jref)******PL_exp_p **+** \ **-**1j ***** J_abs ***** np**.**sin(loss_tan) *****  (f**/**f_Jref)******PL_exp_pp c_f **=** 1**/**(rho ***** J_f)******0.5**;** k_f **=** om/c_f**;** Z_f **=** rho ***** c_f Z_Liq **=** (1j ***** om ***** rho ***** eta_Liq)******0.5 dfc_tot **=** f_fund ***** 1j/(np**.**pi*****Zq) ***** (1j ***** Z_f) ***** \ (Z_f ***** np**.**tan(k_f ***** d_f) **-** 1j ***** Z_Liq)/\ (Z_f **+** 1j ***** Z_Liq ***** np**.**tan(k_f ***** d_f)) dfc **=** dfc_tot **-** f_fund ***** 1j/(np**.**pi*****Zq) *****  Z_Liq **return** dfc **def** fit_model(X_arr**,** Y_arr)**:** y_real**=**np**.**hstack((np**.**real(Y_arr)**,**np**.**imag(Y_arr)))#Compl. fit func. **def** func(Pars)**:** ymodel **=** Calc_dfc_from_Box_Profile(X_arr**,**Pars) ymodel_real **=** np**.**hstack((np**.**real(ymodel)**,** np**.**imag(ymodel))) **return** y_real **-** ymodel_real FitPars**,** ier **=** leastsq(func**,** GuessPars) Y_fit **=** Calc_dfc_from_Box_Profile(X_arr**,**FitPars) dev_sqr **=** np**.**sum(np**.**abs(Y_arr **-** Y_fit)******2) **return** FitPars**,**Y_fit**,**dev_sqr 
**def** Analyze ()**:**
times**,**dfc_expt **=** read_dfcs()**;** dfc_fit**=**np**.**zeros*****np**.**zeros((len(times)**,**len(ovt_ord))**,**dtype**=**complex)**;** d_f_fit            **=** np**.**nan ***** np**.**zeros(len(times)) J_abs_fit      **=** np**.**nan ***** np**.**zeros(len(times)) dev_sqr_fit    **=** np**.**nan ***** np**.**zeros(len(times)) loss_tan_fit    **=** np**.**nan ***** np**.**zeros(len(times)) PL_exp_p_fit **=** np**.**nan ***** np**.**zeros(len(times)) **for** i **in** range(len(times))**:** FitPars**,**Y_fit**,**dev_sqr**=**fit_model(ovt_ord**,**dfc_expt**[**i**]**) d_f_fit**[**i**]            =** FitPars[0]**;** J_abs_fit**[**i**]      =** FitPars[1] loss_tan_fit**[**i**]    =** FitPars[2]**;** PL_exp_p_fit**[**i**] =** FitPars[3] dev_sqr_fit**[**i**]  =** dev_sqr**;** dfc_fit**[**i**]             =** Y_fit**;** **import** numpy **as** np**; from** scipy**.**optimize **import** leastsq eta_Liq **=** 1e-3**;** rho **=** 1e3**;** f_fund **=** 5e6**;** f_Jref **=** 30e6**;** Zq **=** 8.8e6**;** ovt_ord_in **=** np**.**array(**[**1.**,**3.**,**5.**,**7.**,**9.**,**11.**,**13**]**) # data from 5-65 MHz ovt_ord **=** ovt_ord_in**[**1**:-**1**]**# ignore the 5-MHz and 55-MHz overtone Analyze()**;**

## Appendix D. Justification of a Complex ${\tilde{k}}_{q}$ in the Lu-Lewis Equation

The original form of the Lu-Lewis equation was based on a real-valued wave vector in the resonator, $k_{q}$. In order to account for lossy samples, $k_{q}$ was turned in to an effective complex ${\tilde{k}}_{q}\;$ in Equation (20). The imaginary part is not linked to the resonator’s intrinsic losses. If the sample is lossy, the displacement at *z* = *d*_q_ is of the form:

(A22) $$\begin{array}{cc} \hat{u}{(d}_{q},t) & \;=\;{\hat{u}}_{S}\cos\left({k_{q}}^{\prime }d_{q}\right)\exp\left(i{\tilde{\omega }}_{\mathrm{res}}t\right)\\ & \;=\;{\hat{u}}_{S}\cos\left({k_{q}}^{\prime }d_{q}\right)\left(1\;+\;i\phi \right)\exp\left({{i\omega }_{\mathrm{res}}}^{\prime }t\right)\\ & \;=\;{\hat{u}}_{S}\left(\cos\left({k_{q}}^{\prime }d_{q}\right)\;+\;i\cos h\left({k_{q}}^{\prime \prime }d_{q}\right)\right)\exp\left({{i\omega }_{\mathrm{res}}}^{\prime }t\right)\\ & \;=\;{\hat{u}}_{S}\cos\left({\tilde{k}}_{q}d_{q}\right)\exp\left({{i\omega }_{\mathrm{res}}}^{\prime }t\right) \end{array}$$

A phase contained in the complex resonance frequency ${\tilde{\omega }}_{\mathrm{res}}$ was lumped into $k_{q}$. The phase is caused by the lossy sample. Again, ${\tilde{k}}_{q}$ is an effective wave number, introduced to keep the algebra simple. Because the apparent ${k_{q}}^{\prime \prime }$ is small, the errors can be tolerated. (One is not forced to do that. The calculation also runs along with complex ${\tilde{\omega }}_{\mathrm{res}}$.)
